# International incidence of psychotic disorders, 2002–17: a systematic review and meta-analysis

**DOI:** 10.1016/S2468-2667(19)30056-8

**Published:** 2019-05-01

**Authors:** Hannah E Jongsma, Caitlin Turner, James B Kirkbride, Peter B Jones

**Affiliations:** a Department of Psychiatry, University of Cambridge, Cambridge, UK; b Institute of Public Health, University of Cambridge, Cambridge, UK; c PsyLife Group, Division of Psychiatry, University College London, London, UK; d CAMEO, Cambridgeshire and Peterborough NHS Foundation Trust, Cambridge, UK

## Abstract

**Background:**

The last comprehensive systematic review of the incidence of psychotic disorders was published in 2004. New epidemiological data from different settings now permit a broader understanding of global variation. We examined the variation in psychosis by demographic characteristics and study method.

**Methods:**

For this systematic review and meta-analysis, we searched PubMed, Embase, Web of Science, PsycINFO, and bibliographies, and directly contacted first authors. We sought to obtain citations of original research published between Jan 1, 2002, and Dec 31, 2017, on incidence of non-organic adult-onset psychotic disorder. We included papers that were published or in grey literature and had no language restrictions. Data were extracted from published reports, where possible, by sex, age, and ethnic group. Quality of yield was assessed. Data were assessed using univariable random-effects meta-analysis and meta-regression. We registered our systematic review on PROSPERO, number CRD42018086800.

**Findings:**

From 56 721 records identified, 177 met inclusion criteria. The pooled incidence of all psychotic disorders was 26·6 per 100 000 person-years (95% CI 22·0–31·7). Heterogeneity was high (*I*^2^≥98·5%). Men were at higher risk of all psychotic disorders (incidence rate ratio 1·44 [1·27–1·62]) and non-affective disorders (1·60 [1·44–1·77]) than women, but not affective psychotic disorders (0·87 [0·75–1·00]). Ethnic minorities were also at excess risk of all psychotic disorders (1·75 [1·53–2·00]), including non-affective disorders (1·71 [1·40–2·09]). Meta-regression revealed that population registers reported higher rates of non-affective disorders (9·64 [2·72–31·82]), schizophrenia (2·51 [1·24–5·21]), and bipolar disorder (4·53 [2·41–8·51]) than first contact study designs.

**Interpretation:**

We found marked variation in incidence of psychotic disorders by personal characteristics and place. Some geographical variation could be partially explained by differences in case ascertainment methods.

**Funding:**

None.

## Introduction

Psychotic disorders are associated with substantial premature mortality,^1^, ^2^ morbidity,^3^ and a large social and financial burden.^4^ Yet, research into their distribution and determinants has only in the past decade extended beyond North America^5^ and northern Europe^6^, ^7^, ^8^ to southern Europe,^9^, ^10^, ^11^, ^12^ South America,^13^ Africa,^14^, ^15^ and other low-income and middle-income countries (LMICs).^15^, ^16^ These new data might provide new clues to the determinants of the heterogeneity in the incidence of psychotic disorders between and within different populations reported in previous studies,^17^, ^18^ aiding both service planning and our understanding of cause; both are crucial for planning effective public mental health responses. The most recent comprehensive systematic review and meta-analysis^17^ was published in 2004 and was restricted to schizophrenia. Further meta-analyses have limitations in terms of single country coverage,^18^ search scope, yield and assessment of heterogeneity,^19^ specific population group coverage^20^, ^21^ or coverage of a particular risk factor,^22^, ^23^, ^24^, ^25^, ^26^ or were also restricted to schizophrenia.^27^

Together, these reviews showed that estimates of the incidence of psychotic disorders vary across replicable demographic, geographical, and social characteristics. Men and young people appear to have an excess risk,^27^, ^28^ as do migrants and their descendants.^20^, ^29^, ^30^ Settings at higher latitude and more urban settings also yield higher incidences.^26^, ^31^ Socioeconomic deprivation, inequality, and instability are also associated with increased incidence.^14^, ^32^, ^33^, ^34^ Earlier meta-analyses^17^, ^18^, ^35^ found no evidence of variation in incidence by study quality or other methodological features. Research suggests^36^, ^37^ that higher incidences are derived from population registers (which cover all health-care contacts within an entire health system) than from first-contact studies (which rely on individuals making contact with appropriate services). These comparisons notwithstanding, methodological heterogeneity as an explanation for variation in incidences has not been investigated widely.

We sought to synthesise the accumulating research on the incidence of adult-onset psychotic disorders (including affective psychotic disorders) and investigate whether sociodemographic factors or methodological heterogeneity accounted for any observed variation. Consistent with available evidence, we hypothesised that incidences would be higher in men, younger people, and those from ethnic minority groups, and in register-based studies.

**Research in context**

**Evidence before this study**

We searched PubMed and Web of Science (appendix p 4) for international systematic reviews and meta-analyses of the incidence of non-organic psychotic disorders in the general population, published since the last major review of the evidence (published in 2004). Our search yielded 156 results, of which 14 were meta-analyses. However, these commonly examined a single risk factor for psychotic disorders, such as migrant status, or synthesised evidence of incidence in a particular segment of the population, such as the elderly. Only one meta-analysis met all inclusion criteria and summarised incidence in the general population, but this study provided no assessment of heterogeneity.

**Added value of this study**

To the best of our knowledge, this study is the first comprehensive systematic review and meta-analysis of the incidence of non-organic adult-onset psychotic disorders done in 16 years and provides an update on the epidemiological landscape. For the first time, we also formally assessed if incidence of psychotic disorders varies by study type. Incidence varied substantially between settings: a 10 times variation in incidence was observed across diagnostic categories. We also found that studies with routine registers reported higher incidences of disorder than studies with a service-based design.

**Implications of all the available evidence**

Variance in the incidence of psychotic disorders worldwide arises from both replicable social, demographic, and environmental determinants, and from methodological heterogeneity. Although most studies continue to be done in a handful of countries, future studies across more diverse settings will benefit from standardised methods to facilitate comparable estimates of incidence across the globe.

## Methods

### Search strategy and selection criteria

This systematic review and meta-analysis followed PRISMA guidelines^38^ (appendix pp 2–3), including preregistering our protocol with PROSPERO (CRD42018086800) before extraction of data. Our method is based on a previous systematic review.^18^

We systematically searched PubMed, PsycINFO, Web of Science, and Embase, adapting a previously used search strategy^18^ based on Cochrane Systematic Reviewing guidelines.^39^ This strategy used terms covering psychotic disorders and incidence and was adapted for each database (appendix p 4). We searched bibliographies of included citations and directly contacted authors to request data, where appropriate. We restricted our review to studies published between Jan 1, 2002, and Dec 31, 2017. We had no restriction on language of publication, study design, or publication status, although grey literature was only identified via published conference proceedings, author correspondence, and bibliographical searches.

Citations were considered eligible if they contained incidence data or data from which incidence could be derived (numerator and denominator); included patients (aged 18–64 years) diagnosed with a first episode of any psychotic disorder; were published between Jan 1, 2002, and Dec 31, 2017, and were published in the scientific or grey literature, online, or in print.

Two authors (HEJ, CT) carried out searches and screened the titles found to assess whether they met eligibility criteria, with definite or possible titles forwarded to duplicate independent abstract review and, if appropriate, full text review. Uncertainties about inclusion were resolved in agreement with two senior authors with experience in epidemiological research and systematic reviewing (JBK, PBJ). The study protocol is available online.

### Data analysis

Two authors (HEJ, CT) extracted data. Study-level data about study characteristics, rate-level data about incidences, and meta-level data on time period, study quality, study design, and diagnostic criteria (see below) were included.

The primary outcome was incidence per 100 000 person-years of all psychotic disorders (International Classification of Disease tenth edition [ICD-10] = equivalent, F20–33), non-affective disorders (F20–29), schizophrenia (F20), affective disorders (F30–33), bipolar disorder with psychosis (F30–31), psychotic depression (F32–33), or substance-induced psychosis (F1X.5). Included studies used a range of diagnostic classifications, including ICD-8, ICD-9, and ICD–10, and the Diagnostic and Statistical Manual of Mental disorders (DSM) versions 3-R and 4, and we assumed sufficient commonalities to pool citations (appendix p 5).

Where possible, we extracted summary-level incidence data on the exposures age, sex, ethnicity, and migrant status. Meta-level data on study design, study quality, and time period were recorded. Study design was divided into first-contact studies (which count the number of people attending the relevant service, and include first presentation, first diagnosis, first GP record, first admission, and first treatment), cohort studies, case-register studies (with a dedicated national patient register), and studies with a general population register covering an entire health system. Time period was defined as the median year of the case ascertainment period. Where incidences were not directly reported, we derived them from ancillary information wherever possible. Where citations reported overlapping data from the same study or population, we used set criteria to establish inclusion (appendix p 4).

The full spreadsheet containing all study-level, rate-level, and meta-level data is available online.

Two independent raters (HEJ, JBK or CT) assessed study quality according to seven previously published criteria:^18^ designation of a defined catchment area, accurate reporting and reliable source of denominator data, population-based case finding, standardised research diagnosis used, masking (of the clinician) to demographic variables, inclusion criteria stated, and inclusion of a leakage study (appendix p 4).

We first did a narrative synthesis of the yield. Based on previous meta-analyses,^17^, ^18^ we anticipated high levels of heterogeneity and therefore specified use of random-effects meta-analysis and meta-regression a priori to quantify this heterogeneity. When five or more incidences could be pooled, we did random-effects meta-analyses using the DerSimonian and Laird method,^40^ grouping citations by study design. We transformed incidence rates to their natural logarithm and entered into meta-analyses with corresponding standard errors (SE)s. If no SE could be derived, we retained studies for narrative synthesis only. For assessments of differences in incidence by sex and ethnicity, we estimated incidence rate ratios (IRRs), transformed them to their natural logarithm, and entered them into meta-analyses with their corresponding SEs.

We assessed statistical heterogeneity using the *Q* test and quantified using the *I*^2^ statistic, which identifies the proportion of the observed variance that reflects real differences in effect size. We examined evidence of small study effects (including publication bias) by visual inspection of funnel plots and formal testing using Egger's test for which at least 10 estimates were available.^41^ We did random-effects meta-regression to explore whether heterogeneity was associated with study quality, study design, or time period.

We did meta-analyses in Stata (version 13)^42^ using the metan and admetan commands. We did meta-regressions using the metareg package, and we did funnel plots and Egger's tests using the metafunnel and metabias packages.

We chose to display pooled estimates to prevent ad-hoc summaries of data but considering the high expected heterogeneity, the emphasis in interpretation of results is on the variation in incidences.

### Role of the funding source

There was no funding source for this study.

## Results

**Figure 1:**
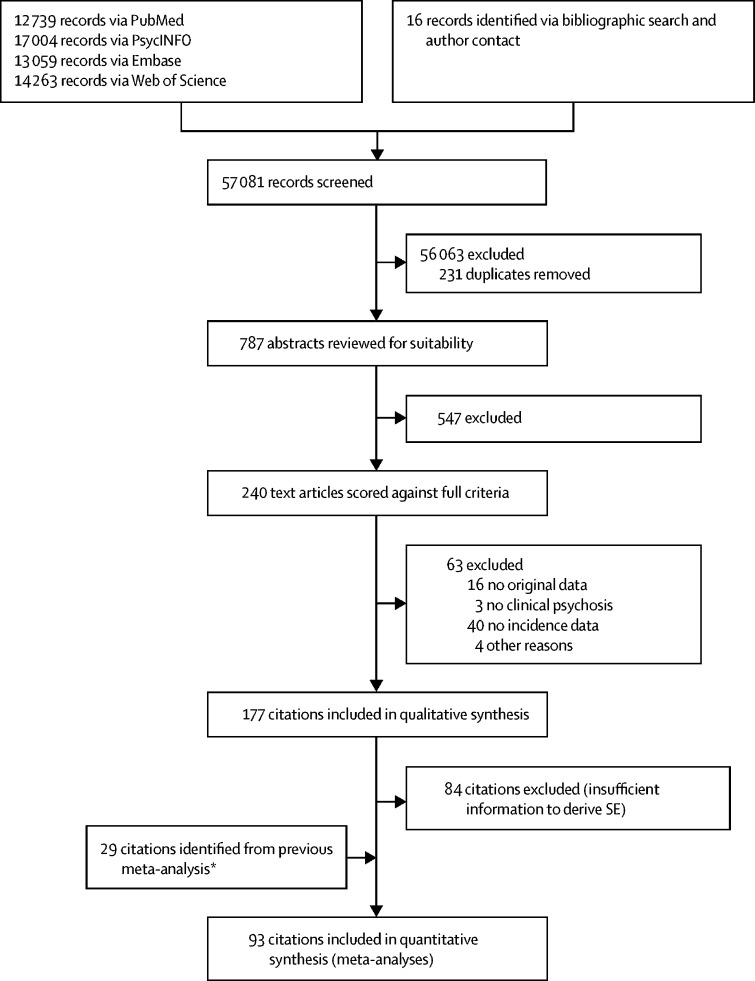
PRISMA flowchart *Citations derived from Kirkbride and colleagues,^18^ which cover England only from 2002–09.

We retrieved 56 721 records of which 177 met inclusion criteria (figure 1; table); 93 (53%) of 177 had sufficient data available for meta-analysis and meta-regression. Most studies (140 [79%] of 177) were done in Europe, with 14 (8%) done in North America. Few studies were done in Asia (11 [6%]), the Middle East (seven [4%]), Australia, Latin or South America (four [2%] each), or Africa (two [1%]). Two citations covered more than one continent.^15^, ^55^ Citations examining psychosis in young people (26 [15%]), comorbid groups (12 [7%]), the army (seven [4%]), a prison population (one [<1%]), and post-partum psychosis (five [3%]) are synthesised in the appendix (pp 8–12) because they are not representative of the general population. The most frequently studied diagnostic outcome was schizophrenia (86 [49%]), followed by all non-affective disorders (66 [37%]) and all psychotic disorders (59 [33%]). Any affective psychotic disorder as an outcome was less frequently studied (32 [18%]), although we identified 40 (22%) citations of bipolar disorder with psychosis and 15 (8%) citations of psychotic depression. Six (3%) citations examined substance-induced psychosis.

**Table:** Study characteristics of included citations

	**Country**	**Period**	**Type**	**Diagnostic confirmation**	**Diagnostic classification**	**Diagnostic outcomes**	**Number of cases**
Tsuchiya et al 2002^61^*	Denmark	1980–97	First admission	..	ICD-8, ICD-10	Schz	Unknown
Hanoeman et al 2002^62^*	Surinam	1992–93	First admission	Medical records	DSM-3-R	Schz, schzp	73
Selten et al 2002^63^†	Netherlands	1970–92	Case register	None	ICD-8, ICD-9	Schz	Unknown
Baldwin et al 2002^64^‡	Ireland	1995–2000	First contact	SCID or medical records	DSM-4	FEP	69
Scully et al 2002^65^*	Ireland	1995–2000	First contact	SCID or medical records	..	FEP	69
Boydell et al 2003^66^*	England	1965–97	Case register	Case notes plus OPCRIT	Combination	Schz	623; 385
Smith et al 2003^67^‡	Canada	1907–13	First admission	Clinical records	DSM-4	Schz, schzp, bpd	831
Singh et al 2003^68^	England	2000	First contact	Interview, questionnaire, case notes	..	FEP	295
Selten et al 2003^69^	Netherlands	1990–96	Case register	Discharge summary	ICD-9	Bpd, pd	14 749
Cantor-Graae et al 2003^70^‡	Denmark	1970–98	Population register	None	ICD-8, ICD-10	Schz	10 244
Baldwin et al 2003^71^‡	Ireland	1995–2002	First contact	SCID or clinical records	DSM-4	FEP, non-aff, schiz, aff, bp, pd, other	146
Proctor et al 2004^72^	England	1998–2001	Case register	Chart diagnosis	ICD-10	FEP, non-aff, schz, aff, bp, pd, sip, other	227
Sipos et al 2004^73^	Sweden	1989–2001	First admission	None	ICD-9, ICD-10	Non-aff, schz	1950
Chien et al 2004^74^‡	Taiwan	1997–2001	First contact	None	ICD-9	Schz	419
Boydell et al 2004^75^*	England	1988–97	Combination	Case records using OCCPI	RDC	Schz	222
Veen et al 2004^76^	Netherlands	1997–99	First contact	Diagnostic meeting	DSM-4	FEP, non-aff, aff, oth	181
Singh et al 2004^77^	England	1992–94	First contact	Interview, SCAN or SANS and OCCPI or OPCRIT	ICD-10	FEP, non-aff, schz, aff, sip, oth	168
Sailas et al 2005^78^§	Finland	1984–94	Cohort	None	Other	FEP	71
Harris et al 2005^79^*†	Australia	..	First presentation	Consensus	DSM-4, ICD-10	FEP, schz, aff	94
Sundquist et al 2005^80^‡	Sweden	1997–99	Population register	None	ICD-9, ICD-10	FEP, pd	6163
Nager et al 2005^81^¶	Sweden	1986–97	Cohort	None	ICD-9, ICD-10	FEP	339
Laursen et al 2005^82^‡	Denmark	1952–87	Population register	None	ICD-8, ICD-10	Schz, schza, bp	18 147
Selten et al 2005^83^	Surinam	2002–03	First contact	CASH interview, panel discussion	DSM-4	FEP	64
Nixon et al 2005^84^	England	1881–1994	Combination	Case notes	RDC	Schz	41
Qin et al 2005^85^‖	Denmark	1950–87	Population register	None	ICD-8, ICD-10	Non-aff, schz	795
Allardyce et al 2005^86^	Scotland	1989–93	First admission	Case record	ICD-9	FEP	5838
Cantor-Graae et al 2005^87^	Sweden	1999–2001	First contact	Clinical, case records, additional data	DSM-4	FEP, non-aff	150
Baldwin et al 2005^43^	Ireland	1995–2003	First contact	SCID or clinical diagnosis	DSM-4	FEP, non-aff, schz, aff, bpd, pd, oth	194
Kennedy et al 2005a^88^	England	1965–99	Combination	Case notes plus OPCRIT	DSM-4	Bpd	246
Kennedy et al 2005b^89^*	England	1965–99	Combination	Case notes plus OPCRIT	DSM-4, ICD-10	Bpd	246; 235
Lloyd et al 2005^90^‡	England	1997–99	First contact	Interview (SCAN, SANS, modified PPHS), consensus diagnosis	ICD-10	Bpd	75
Leão et al 2006^8^†	Sweden	1992–99	Population register	None	ICD-9, ICD-10	Non-aff	Unknown
Bray et al 2006^91^†	Canada	1975–85	First contact	None	ICD-10	Schz	1962
Payne et al 2006^92^	Canada	1993–95	First admission	Clinical records	..	Non-aff	146
Drukker et al 2006^93^	Netherlands	1993–2002	Case register	None	DSM-4	Schz	98
Turner et al 2006^94^**	England	1999–2002	First admission	Case notes, ratified by psychiatrist	ICD-10	Non-aff, schz	62
Mahmmood et al 2006^95^	England	2005–05	First contact	Unknown	..	FEP	303
Westman et al 2006^96^‡	Sweden	1997–98	Population register	None	ICD-9, ICD-10	FEP	10 800
Munk-Olsen et al 2006^97^¶	Denmark	1955–90	Population register	None	ICD-8, ICD-10	Non-aff, schz	166
Smith et al 2006^44^	Canada	1902–13	First admission	Clinical records	DSM-4	Schz, schza, schp, oth	807
Amminger et al 2006^98^†	Australia	1997–2000	First treatment	Youth assessment team, random sample SCID or RPMIP	DSM-4	FEP	1019
Veling et al 2006^7^	Netherlands	1997–2005	First contact	Diagnostic meeting	DSM-4	Non-aff	181
Morgan et al 2006^99^‡	England	1997–99	First contact	Interview (SCAN), case notes, consensus meeting	ICD-10	FEP, schz	592
Fearon et al 2006^100^*	England	1997–99	First contact	Interview (PSE SCAN), case notes (IGC SCAN)	ICD-10	FEP, schz, bpd, pd, oth	568
Gould et al 2006^101^	England	1997–99	First presentation	WHO screening for psychosis plus OPCRIT	ICD-10	FEP	111
Kirkbride et al 2006^6^	England	1997–99	First contact	SCAN, consensus panel	DSM-4	FEP, non-aff, schz, aff, sip	568
Zipursky et al 2006^102^	England	1997–99	First contact	SCAN, consensus panel	DSM-4	FEP, schz	Unknown
Li et al 2007^103^	Sweden	1984–2004	Population register	None	ICD-9, ICD-10	FEP	40 228
Schimmelmann et al 2007^104^*	Australia	1998–2000	First admission	SCID and clinical diagnosis comparison	DSM-4	FEP	636
Laursen et al 2007^105^	Denmark	1995–87	Population register	None	ICD9, ICD-10	Schz, bpd	17 787
Ajdacic-Gross et al 2007^106^†	Switzerland	1977–2005	Case register	None	ICD-8, ICD-9	FEP, schz	7230
Andersen et al 2007^107^	Norway	1887–2005	First admission	Case records	ICD-10	Schz, aff	64
Harlow et al 2007^108^	Sweden	1987–2001	Cohort	None	ICD-8, ICD-9	Non-aff, schz, schza, bpd	2134
Juvonen et al 2007^109^‖	Finland	1950–59	Population register	Case notes (2 experts)	DSM-4	Schz	807
Cantor-Graae et al 2007a^110^‡	Denmark	1986–2006	Population register	None	ICD-8, ICD-10	Schz	4609
Cantor-Graae et al 2007b^111^‡	Denmark	1970–2001	Population register	None	ICD-8, ICD-10	Schz	10 779
Leão et al 2007^112^*†	Sweden	1995–98	Population register	None	ICD-9, ICD-10	Non-aff, aff	Unknown
Kikbride et al 2007a^113^	England	1997–99	First contact	SCAN, consensus panel	ICD-10	FEP, non-aff, aff	295
Menezes et al 2007^13^	Brazil	2002–2004	First contact	SCID-I or case notes	DSM-4	FEP, non-aff, aff	367
Kirkbride et al 2007b^114^‡	England	1997–99	First contact	SCAN, consensus panel	ICD-10	Non-aff, schz, oth	218
Stain et al 2008^115^†	Australia	2001–2005	First contact	Case notes	Other	Oth	308
Boonstra et al 2008^116^	Netherlands	2002	First contact	Clinical diagnosis	DSM-4	Non-aff	75
Crebbin et al 2008^117^*	England	1998–2005	Case register	Chart diagnosis	ICD-10	FEP, schz, pd	540
Farquhar et al 2008^45^	Wales	1875–2005	First admission	Case records	ICD-10	Schz, schza, aff, bpd, pd, oth	579
Pelayo-Teran et al 2008^10^	Spain	2001–05	First contact	SCID-I	DSM-4	Non-aff	174
Castagnini et al 2008^118^*	Denmark	1996	Case register	None	ICD-8	Schz, bpd, oth	11 126
Burns et al 2008^14^	South Africa	2005	First presentation	Case records	DSM-4	FEP	160
Weiser et al 2008^119^	Israel	..	Population register	None	ICD-9, ICD-10	Schz	1686
Veling et al 2008^120^	Netherlands	1997–2005	First contact	Diagnostic meeting	DSM-4	FEP, non-aff, bpd, pd, oth	466
Kirkbride et al 2008a^121^‡	England	1997–99	First contact	SCAN, consensus panel	ICD-10	Schz	148
Kirkbride et al 2008b^122^	England	1996–2000	First contact	SCAN, consensus panel	DSM-4	FEP, schz, non-aff, oth	484
Coid et al 200^123^	England	1996–2000	First contact	SCAN, consensus panel	DSM-4	FEP, non-aff, schz, aff, oth	484
Grant et al 200^124^††	USA	2004–05	Cohort	Not stated	DSM-4	Bpd	263
Crebbin et al 2009^125^*	England	1998–2005	Case register	Chart diagnosis	ICD-10	Schz, sip	430
Bih et al 2009^126^*††	Taiwan	1996–2003	Cohort	None	ICD-9	Bpd	532
Corcoran et al 2009^127^†	Israel	1964–97	Cohort	None	ICD-10	Non-aff	637
Osby et al 2009^128^*	Sweden	1997–2005	Case register	None	ICD-10	Bpd	4117
Valdimarsdottir et al 2009^129^¶	Sweden	1983–2000	Cohort	None	ICD-8, ICD-9	FEP	4557
Harlap et al 2009^130^	Israel	1964–76	Cohort	None	ICD-10	Schz	637
Reay et al 2009^131^	England	1998–2005	First contact	Chart diagnosis	ICD-10	FEP, non-aff, schz, aff, bpd, pd	540
Norredam et al 2009^132^	Denmark	1994–2003	Cohort	None	ICD-10	Non-aff	1127
Bogren et al 2009^133^	Sweden	1947–97	First contact	Key informants, case files	DSM-4	Non-aff, schz, schza, aff, bpd	61
Kirkbride et al 2009^134^‡	England	1978–99	Combination	SCAN, consensus agreement	ICD-9, ICD-10	FEP, non-aff, schz, aff, bpd, pd, sip, oth	347
Coid et al 2009^123^	..	..	..	..	..	..	..
Cheng et al 2010^135^†	England	2002–2007	First contact	Unsure	ICD-10	FEP	285
Bogren et al 2010^46^	Sweden	1947–97	First presentation	Key informants, case files	DSM-4	Non-aff, aff, bpd	108
Zammit et al 2010^136^‡	Sweden	1972, 1977	First admission	None	ICD-8, ICD-9	Non-aff, schz	881
Tseng et al 2010^137^	Taiwan	1996–2001	First hospitalisation	None	ICD-9	Schz	Unknown
Zandi et al 2010^138^	Netherlands	2002–04	First contact	CASH or CASH-CS, medical files, consensus diagnosis	DSM-4	FEP, schz	77
Norredam et al 2010^139^‡	Denmark	1994–2003	Cohort	None	ICD-10	Non-aff	791
Goodman et al 2011^47^**	USA	..	First contact	Not stated	ICD-9	FEP	8
Cowan et al 2011^140^**	USA	2000–09	First hospitalisation	None	ICD-9	Non-aff	2722
Harris et al 2011^141^*	Wales	1875–2005	First admission	Panel assessment of case notes	ICD-10	Pd	800
Jorgensen et al 2011^142^	Sweden	2005	Case register	Random sample checked by psychiatrist	ICD-10	Non-aff, schz	416
Cheng et al 2011^143^†	England	2002–07	First contact	Multidisciplinary diagnostic meeting	ICD-10	FEP	285
Kleinhaus et al 2011^144^†	Israel	1964–76	Cohort	None	ICD-10	Non-aff	860
Benros et al 2011^145^‖	Denmark	1945–96	Population register	None	ICD-8, ICD-10	Non-aff	39 076
Salokangas et al 2011^146^	Finland	..	Case register	None	ICD-8, DSM-3-R, ICD-10	Schz	30 032
Schofield et al 2011^147^	England	1996–2006	First GP record	Patient records	READ codes	FEP	508
Veling et al 2011^148^	Netherlands	1997–2005	First contact	Diagnostic meeting	DSM-4	FEP	618
Healy et al 2012^149^*	Wales	1875–2005	First admission	Case records, clinical diagnosis	ICD-10	Schz, oth	3523
Callaghan et al 2012^150^*	USA	1990–2000	First hospitalisation	Not stated	ICD-9	Schz	1499
Anderson et al 2012^151^†	Canada	2000–06	First contact	None	..	Non-aff	546
Manrique-Garcia et al 2012^152^**	Sweden	1969–70	First admission	None	ICD-8, ICD-9	Non-aff, schz	674
Turola et al 2012^153^	Italy	1979–2008	First diagnosis	Case notes	DSM-4, ICD-10	Schz	1759
Werbeloff et al 2012^154^	Israel	1979–92	Case register	None	ICD-9	Schz	2335
Nosarti et al 2012^155^†	Sweden	1973–85	First admission	None	ICD-8 and ICD-9	Non-aff, bpd	886
Gigantesco et al 2012^156^	Italy	2008	First contact	SCID-I, BPRS, GAF in duplicate	DSM-4	FEP, bpd	247
Tarricone et al 2012^11^	Italy	2002–09	First contact	SCAN, consensus diagnosis	ICD-10	FEP, Non-aff, schz, aff	163
Kirkbride et al 2012^157^†	England	2009–11	First presentation	Clinical diagnoses	ICD-10	FEP	..
Hung et al 2013^158^‖††	Taiwan	2000–05	Cohort	None	ICD-9	Bpd	9711
Peritogiannis et al 2013^159^	Greece	2008–09	First contact	None	ICD-10	FEP	132
Sutterland et al 2013^160^	Netherlands	1996–2006	First GP record	Medical records	ICPC	Non-aff, schz	293
Cantor-Graae et al 2013^161^†‡	Denmark	1995–2010	Population register	None	ICD-8, ICD-10	Non-aff, schz, schza, bpd	13 729
Kroon et al 2013^162^	Netherlands	1996–2007	First GP record	Medical records	ICPC	Bpd	649
Castagnini et al 2013^163^‡	Denmark	1995–2008	First diagnosis	None	ICD-10	Oth	11 126
Hardoon et al 2013^164^	England	2000–10	First record or diagnosis	GP records	READ	Schz, bpd, oth	10 520
Weibell et al 2013^165^	Norway	2007–11	First presentation	SCID	DSM-4	Non-aff, sip	321
Cocchi et al 2014^166^	Italy	2007–09	First contact	ERIaos-CL, sociodemographic form, HoNOS, BPRS, WHO-DAS III	ICD-10	Non-aff	43
Tortelli et al 2014^167^	France	2005–09	First admission	Case notes	ICD-10	FEP	258
Hogerzeil et al 2014^37^	Netherlands	2000–05	First contact and case register	Diagnostic meeting and clinical regularly audited)	DSM-4	Schz	254; 843
Pedersen et al 2014^168^*	Denmark	1995–2006	Case register	None	ICD-10	Oth	Unknown
Sørensen et al 2014^169^*	Denmark	1993–95	Population register	None	ICD-8, ICD-10	Schz	17 389
Munk-Olsen et al 2014^170^¶	Denmark	1960–95	First treatment	None	ICD-8, ICD-9	Oth	Unknown
Szoke et al 2014^171^	France	2010–12	First contact	Identical procedures, regular meetings	DSM-4	FEP, non-aff, aff	133
Bhavsar et al 2014^172^†	England	2000–07	First contact	Case notes	RDC	Schz	405
Omer et al 2014^173^‡	Ireland	1995–2000	First contact	SCID or clinical records	DSM-4	FEP	336
Lasalvia et al 2014^9^	Italy	2005–07	First contact	Interview, consensus diagnosis	ICD-10	FEP, non-aff, schz, aff, bpd, pd	558
Veling et al 2014^174^	Netherlands	1997–2005	First contact	Diagnostic meeting	DSM-4	FEP, schz, aff, bpd, pd, oth	618
Kirkbride et al 2014^34^‡	England	1996–2000	First contact	SCAN, consensus diagnosis	DSM-4	Non-aff, aff	484
Anderson et al 2015^5^†	Canada	1999–2008	Population register	Medical records or billing claims		Non-aff	Unknown
Paksarian et al 2015a^175^†	Denmark	1986–2010	Population register	None	ICD-8, ICD-10	Non-aff, schz, bpd	15 811
Sørensen et al 2015^176^	Denmark	1955–67	Population register	None	ICD-8, ICD-10	Non, aff, schz, aff	15 074; 7562
Paksarian et al 2015b^177^	Denmark	1986–2011	Population register	None	ICD-8, ICD-10	Non-aff, schz, bpd	14 285
Soderlund et al 2015^178^†	Sweden	1955–67	Population register	None	ICD-10	Non-aff, schz, aff	2322
Medici et al 2015^179^††	Denmark	1995–2012	Case register	None	ICD-10	Bpd	15 334
Carlborg et al 2015^180^††	Sweden	1991–2010	Case register	None	ICD-10	Bpd	10 273
Tsai et al 2016^181^‖††	Taiwan	2000–07	Cohort	None	ICD-9	Bpd	202
Chen et al 2015^182^‖††	Taiwan	2000–06	Cohort	None	ICD-9-CM	Bpd, pd	118
Latvala et al 2016^183^**	Sweden	1969–2010	Case register	None	ICD-8/9/10	Schz, bpd	14 840
Jensen et al 2016^184^*††	Denmark	1995–2010	Case register	None	ICD-10	Bpd	12 034
Kuhl et al 2016^185^*	Denmark	2000–12	Population register	None	ICD-10	Non-aff, schz	23 479
Filatova et al 2016^186^†	Finland	1966–2013	Cohort	None	ICD-8, ICD-10	Non-aff, schz, bpd, oth	295
Chiang et al 2016^48^	Taiwan	1998–2007	First admission	None	ICD-9-CM	FEP	69 690
Nielsen et al 2016^187^‡	Denmark	1997–2002	Population register	None	ICD-8, ICD-10	Schz	6927
Kendler et al 2016^49^	Sweden	1972–90	Population register	None	ICD-9, ICD-10	Non-aff, schz, bpd	22 589
Levine et al 2016a^188^*	Israel	1950–2004	Cohort	None	ICD-10	Schz	2278
Levine et al 2016b^189^*	Israel	1950–2014	Cohort	None	ICD-10	Schz	665
Vassos et al 2016^50^‡	Denmark	1995–2006	Population register	None	ICD-10	FEP, non-aff, bpd	32 983
Sørensen et al 2016^190^*	Denmark	1930–76	Cohort	None	ICD-8, ICD-10	Schz	4936
Hollander et al 2016^191^†	Sweden	1998–2011	Population register	None	ICD-10	Non-aff	3704
O'Donoghue et al 2016^192^	Ireland	2006–11	First presentation	SCID	DSM-4	FEP	292
Morgan et al 2016^15^	India, Nigeria, Trinidad	..	First contact	SSP, consensus diagnosis	ICD-10	FEP	147
Tarricone et al 2016^193^	Italy	2002–10	First contact	SCAN	ICD-10	FEP	187
Szoke et al 2016^194^	France	2010–14	First contact	Unclear—senior review if uncertain	DSM-4	Non-aff, aff	212
Mulé et al 2016^12^	Italy	2008–11	First contact	SCAN	ICD-10	FEP, schz, aff, oth	204
Ramsey et al 2017^51^**	USA	2001–14	Cohort	None	ICD-9	Schz, bpd	24 714
Okkels et al 2017^195^‖	Denmark	1985–2001	Population register	None	ICD-8, ICD-10	Non-aff, schz, bpd	9329
Vikstrom et al 2017^196^¶	Sweden	1988–2012	Cohort	None	ICD-8, ICD-10	Non-aff, bpd	91
Wang et al 2017^197^‖	Taiwan	1997–2007	Cohort	None	ICD-9	Schz	238
Lin et al 2017^198^‖††	Taiwan	2001–06	Cohort	None	ICD-9 CM	Bpd	183
Marrie et al 2017a^199^‖	Canada	1989–2012	Case register	None	ICD-9 CM	Schz, bpd	Unknown
Marrie et al 2017b^200^‖	Canada	1984–2013	Case register	None	ICD-9 CM	Schz, bpd	Unknown
Hogerzeil et al 2017^201^	Netherlands	2000–05	First contact or case register	Structured interview or clinical, then consensus	DSM-4	Schz	254; 843
Hoeffding et al 2017^202^	Denmark	1995–2013	Population register	None	ICD-8, ICD-10	Non-aff	31 647
Kim et al 2017^52^	South Korea	2002–13	Cohort	None	ICD-10	Non-aff	9387
Markkula et al 2017^53^	Finland	2011–14	Population register	None	ICD-10	Non-aff, bpd	2905
Nielsen et al 2017^203^‖	Denmark	1955–99	Population register	None	ICD-8, ICD-10	Schz	21 305
Schofield et al 2017^204^	Denmark	1965–97	Population register	None	ICD-8, ICD-10	Non-aff	26 891
Simon et al 2017^54^	USA	2007–13	First contact	None, subset case records	ICD-9	FEP	37 843
Kirkbride et al 2017a^205^†	England	2009–13	First contact	OPCRIT	ICD-10	FEP, non-aff, schz, aff, bpd, pd, sip	687
Kirkbride et al 2017b^206^†	England	2009–13	First contact	OPCRIT	ICD-10	FEP, non-aff, schz, aff	687
Schofield et al 2018^207^‡	Denmark	1965–2013	Population register	None	ICD-8, ICD-10	Non-aff	Unknown
Nyberg et al 2018^208^**‡‡	Sweden	1968–2005	Cohort	None	ICD-8, ICD-9, ICD-10	Non-aff	4641
Barghadouch et al 2018^209^†‡‡	Denmark	1993–2000	Cohort	None	ICD-10	Non-aff	392
Richardson et al 2018^210^†‡‡	England	2009–13	First contact	OPCRIT	ICD-10	FEP, non-aff, schz, aff	0687
Jongsma et al 2018^55^	England, Netherlands, France, Spain, Italy, Brazil	2005–15	First contact	SCAN, CASH, DIGS, SID, or case notes—OPCRIT	ICD-10	FEP, non-aff, aff	2774

All references up to and including 60 are found in the reference list of the main article. References from 61 onwards are found in the appendix (pp 35–43). Aff=affective psychosis. Bpd=bipolar disorder. BPRS=Brief Psychiatric Rating Scale. CASH=Comprehensive Assessment of Symptoms and History. CASH-CS=CASH-Culturally Sensitive. DIGS=Diagnostic Interview for Genetic Studies. DSM=Diagnostic and Statistical Manual. ERIaos-CL=Early Recognition Inventory Retrospective Assessment of Symptoms checklist. FEP=all first episode psychosis. GAF=Global Assessment of Functioning. HoNOS=Health of the Nations Outcome Scale. ICD=International Classification of Disease. Non-aff=non-affective psychosis. OCCPI=Operational Criteria Checklist for Psychotic Illness. OPCRIT=Operational Criteria Checklist for Psychotic Illness. Oth=other. Pd=psychotic depression. PPHS=Personal and Psychiatric History Schedule. PSE=Present State Examination. RDC=Research Diagnostic Criteria. RPMIP=Royal Park Multidiagnostic Instrument for Psychosis. SANS=Scale for the Assessment of Negative Symptoms. SCAN=Schedules Clinical Assessment Neuropsychiatry. Schz=schizophrenia. Schzp=schizophreniform disorder. Schza=schizoaffective disorder. SCID=Structural Clinical Interview for DSM-4. SCID-I=SCID-Axis I disorders. Sip=substance-induced psychosis. SID=Structured Interview for DSM-4. SSP=Screening Schedule for Psychosis. WHO-DAS III=WHO Disability Assessment Schedule.

* Citations with insufficient data to include in quantitative analyses.

† Citations only covering young people (<40 years).

‡ Citations only containing information covered in more detail in other citations.

§ Citations covering a prison population.

¶ Citations covering post-partum psychosis.

‖ Citations covering comorbid populations.

** Citations covering the army only.

†† Citations including psychotic bipolar disorder, but where this can't be clearly differentiated from bipolar disorder more widely (not included in analyses).

‡‡ Published online in 2017.

The largest study in this Article^48^ included 69 690 cases, and the smallest study^47^ identified eight cases. The middle year of recruitment varied from 1908^44^ to 2012,^53^ with most citations (105 [59%]) recruiting between approximately 1995 and 2006. Most studies reported a clearly defined catchment area (174 [98%]), clearly listed their inclusion criteria (166 [94%]), used accurate denominator data (157 [89%]), and employed population-based case-finding (135 [76%]). Few studies done used a standardised research diagnosis (50 [28%]), did a leakage study (28·5 [16%]), or used blinding to demographic variables (18 [10%]; appendix pp 6–8). 92 (52%) citations reflected first contact designs and 76 (43%) used a cohort, case, or population register. The remaining nine (5%) studies used a combination. 40 (23%) citations used a version of the DSM for diagnoses and 118 (67%) used a version of ICD. The remaining 19 (11%) used a combination, used a different diagnostic system, such as the Research Diagnostic Criteria, or it was not reported (three [2%]; table). To confirm clinical diagnoses, 21 (12%) citations used a structured interview instrument only, 19 (11%) reviewed medical records, 14 (8%) used a structured interview followed by consensus diagnosis, 13 (7%) used only an interview without specifying whether an instrument was used, ten (6%) used only a consensus or panel discussion, and five (2%) used a chart or clinical diagnoses. The remaining citations either relied solely on clinical diagnoses in registry data (85 [48%]), or information was not stated (ten [6%]; table).

**Figure 2:**
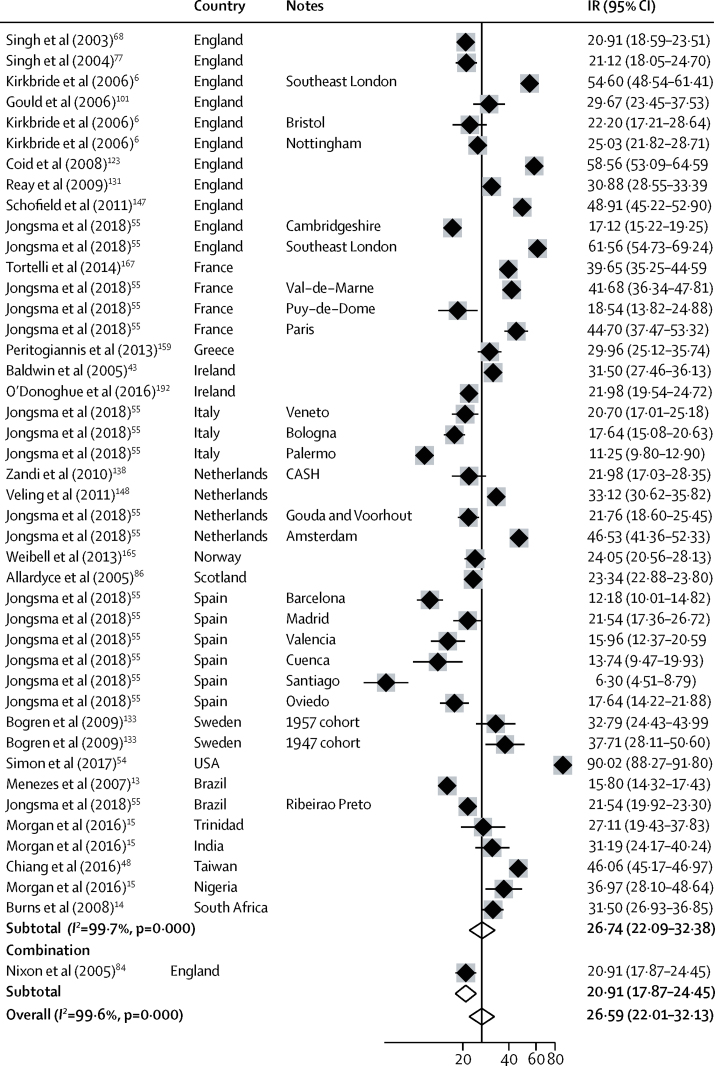
Incidence of all psychotic disorders References from 61 onwards are found in the appendix (pp 35–43). IR=incidence rates. Note: weights are from random effects analysis.

We included 44 separate estimates of the incidence of all psychotic disorders derived from 27 citations, including estimates from multicentre studies (figure 2). Incidence varied around 15 times, from 6·3 per 100 000 person-years (95% CI 4·5–8·8) in Santiago (Spain)^55^ to 90·0 (88·3–91·8) in the USA.^54^ The overall pooled incidence of all psychotic disorders was 26·6 per 100 000 person-years (22·0–31·7).

**Figure 3:**
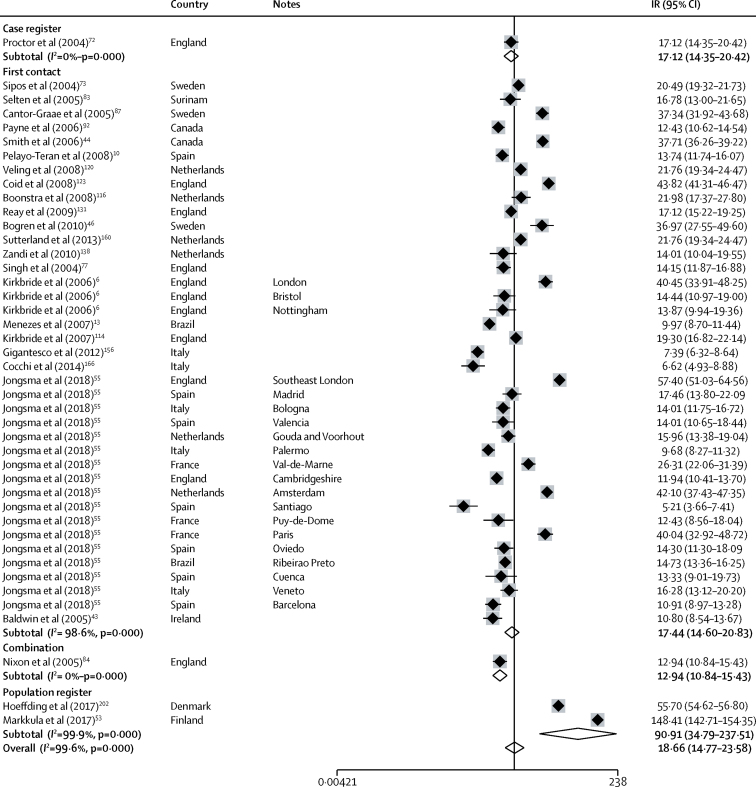
Incidence of non-affective disorders References from 61 onwards are found in the appendix (pp 35–43). IR=incidence rates. Note: weights are from random effects analysis.

**Figure 4:**
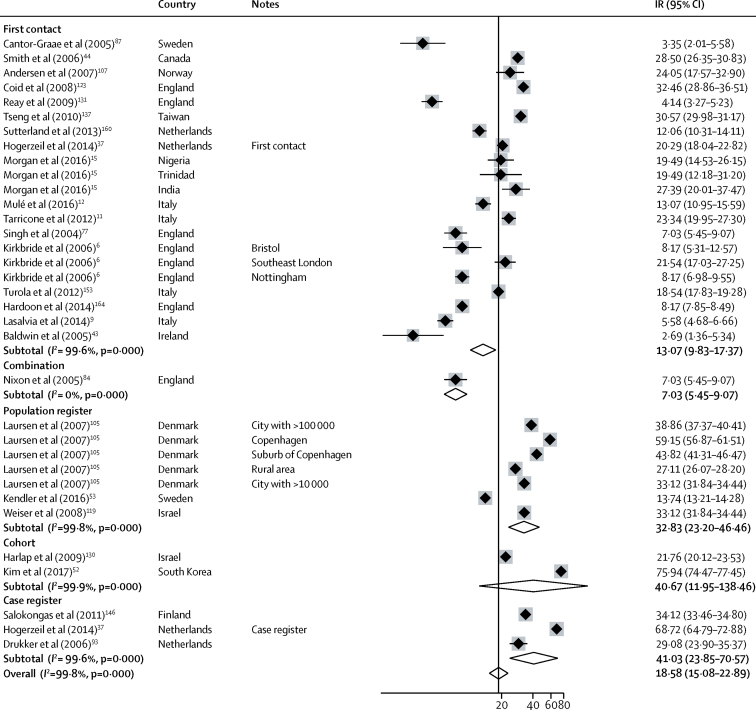
Incidence of schizophrenia References from 61 onwards are found in the appendix (pp 35–43). IR=incidence rates. Note: weights are from random effects analysis.

Incidence of non-affective disorders was available from 47 incidences derived from 28 citations (figure 3). Incidence varied almost 30 times, from 5·2 per 100 000 years (95% CI 3·7–7·4) in Santiago^55^ to 148·4 (142·7–154·4) in Finland.^53^ The overall pooled incidence was 18·7 per 100 000 person-years (14·8–23·6), but this incidence was lower in first-contact studies at 17·4 (14·6–20·8) compared with population register studies (pooled incidence rate 90·9 [34·5–237·5]; figure 3). The incidence of schizophrenia was available from 36 incidences from 26 citations and varied from 2·7 per 100 000 person-years (1·4–5·3) in Cavan-Monaghan (Ireland)^43^ to 75·9 (74·4–77·5) in South Korea.^52^ Pooled incidence was lower in first contact studies (13·1 per 100 000 person-years [9·0–15·0]) than in population registers (32·8 [23·2–46·5]; figure 4).

**Figure 5:**
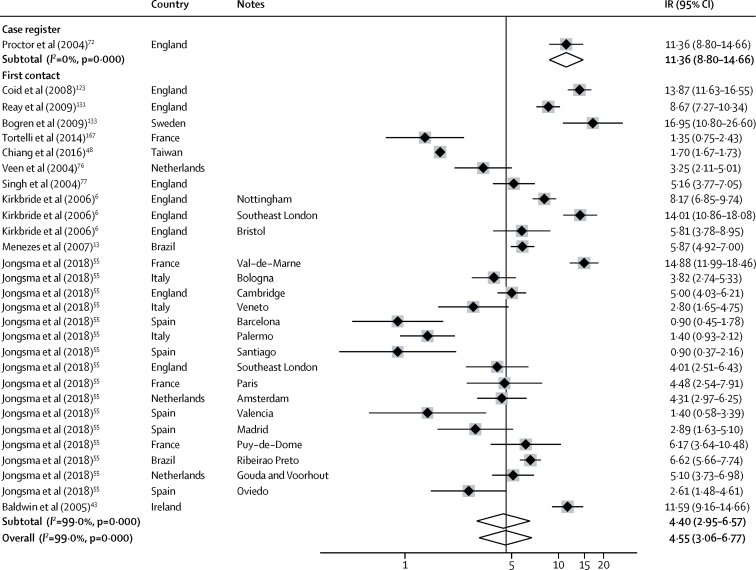
Incidence of affective disorders References from 61 onwards are found in the appendix (pp 35–43). IR=incidence rates. Note: weights are from random effects analysis.

**Figure 6:**
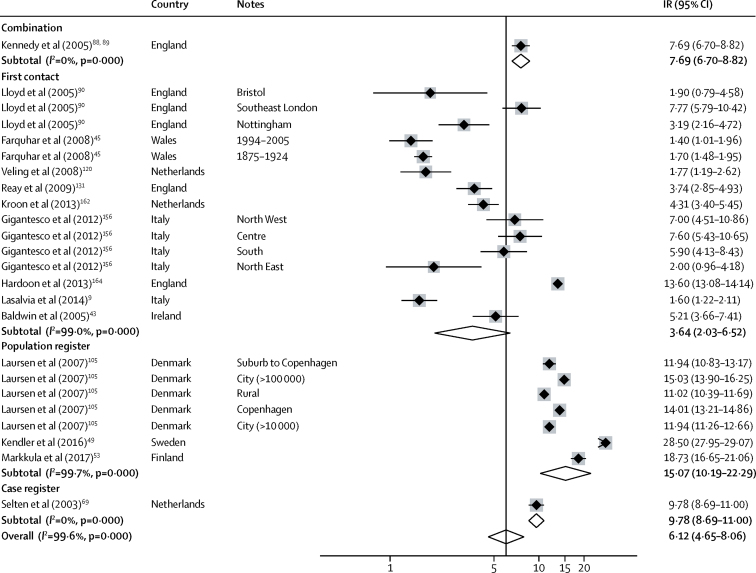
Incidence of bipolar disorder References from 61 onwards are found in the appendix (pp 35–43). IR=incidence rates. Note: weights are from random effects analysis.

We pooled 34 estimates of the incidence of affective psychotic disorders from 16 citations. Incidence varied from 0·9 per 100 000 person-years (95% CI 0·4–2·2) in Santiago^55^ to 17·0 (10·8–26·6) in Lundby (Sweden).^46^ The overall pooled rate was 4·6 per 100 000 person-years (3·1–6·8; figure 5). 24 estimates of the incidence of bipolar disorder were included in a meta-analysis, derived from 15 citations. Incidence varied from 1·4 per 100 000 person-years (1·0–2·0) in Wales^45^ to 28·5 (28·0–29·1) in Sweden,^49^ and was higher in population registers (15·1 [10·2–22·3]) than first contact studies (3·6 [2·0–6·5]; figure 6). Insufficient citations were available to pool rates for other outcomes.

Pooled estimates of the incidence of all psychotic disorders were similar across high-income and LMICs (appendix pp 11–16), though heterogeneity was substantial in both sets of data; formal comparisons were hampered by insufficient studies in LMICs.

For all psychotic disorders, 26 estimates of IRRs in men compared with women were available from 10 citations, with a pooled IRR of 1·44 (95% CI 1·27–1·62). A similar pattern was observed for non-affective psychoses (1·60 [1·44–1·77]; derived from 27 estimates using 11 citations) and schizophrenia (1·70 [1·46–1·97]; derived from 11 estimates using 11 citations). No excess risk in men was found for affective disorders (IRR 0·87 [0·75–1·00]; p=0·07; derived from 20 estimates using six citations) or for psychotic bipolar disorder (0·90 [0·73–1·11]; derived from five estimates; appendix p 17). Insufficient citations were available to pool IRRs for other outcomes.

Migrants and their descendants were at excess risk of all psychotic disorders, non-affective disorders, and schizophrenia (insufficient citations were available to synthesise results for other diagnostic outcomes). When pooling all migrant groups to a binary majority or minority division, 22 estimates from seven citations were available to pool IRRs for all psychotic disorders (pooled IRR 1·75 [95% CI 1·53–2·00]). The pooled IRR for non-affective disorders was 1·71 (1·40–2·09), derived using 28 estimates from thirteen citations. The pooled IRR for schizophrenia was 1·41 (1·15–1·75), derived using six estimates (appendix p 18). Risk was not equitably distributed across ethnic minority groups (appendix pp 19–21).

We did not pool estimates by age group because of the large variety of age groups used. Nonetheless, we observed an overall pattern of higher incidence in younger age groups (appendix pp 22–24). For example, in the multinational EU-GEI study^55^ incidence of all first episode psychosis ranged from 44·2 per 100 000 person-years (95% CI 42·2–46·2) in people aged 18–24 years to 5·5 (3·2–7·7) in people aged 60–64 years.^55^

We found some evidence that study design was associated with variation in incidence. Population registers had higher incidences of non-affective disorders (IRR 9·64 [2·72–31·82]), schizophrenia (2·54 [1·24–5·21]), and bipolar disorder (4·53 [2·41–8·51]) than first contact studies. Incidence of schizophrenia was also elevated in cohort studies (3·10 [1·12–8·53]) and case registers (3·12 [1·33–7·29]). Cohort studies (0·43 [0·20–0·93]) and population registers (0·42 [0·22–0·83]) recorded lower IRRs by minority status for non-affective disorders than first contact designs, but we found no differences by study design in IRRs for any other exposure or outcome association. We found little evidence that study quality and time period were associated with changes in incidence or IRR (appendix pp 25–28).

Heterogeneity was high across study outcomes (*I*^2^ ≥98·5%; Figure 2, Figure 3, Figure 4, Figure 5, Figure 6). Small study effects, as evidenced by Egger's test, were shown in the overall meta-analyses of incidences of all psychotic disorders (β −7·53 [SE 3·14]; p=0·021), non-affective disorders (–14·55 [2·46]; p<0·001), schizophrenia (–11·78 [5·52]; p=0·041), affective disorders (7·72 [1·60]; p<0·001), and bipolar disorder (–14·97 [2·78]; p<0·001). They were also found in analyses by sex for all psychotic disorders (2·16 [0·44]; p<0·001) and affective disorders (0·90 [0·24]; p=0·001), but not for other diagnostic outcomes or for analyses by ethnic group (appendix pp 29–32). Post-hoc sensitivity analyses supported some remaining small study effects within first contact designs (appendix p 32).

## Discussion

Our systematic review identified 177 citations containing data on the incidence of psychotic disorders published since 2002. This yield is considerably higher than reported in another systematic review^19^ and was marked by substantial heterogeneity in incidence across all major psychotic disorders. Although we found no evidence that incidences varied with study quality or time period, we did observe strong evidence of higher incidence rates reported in register-based or cohort-based study designs than in first-contact studies. Given that register-based or cohort-based studies are often done with whole population samples (ie, the USA,^51^, ^54^ Sweden,^49^ Denmark,^50^ Taiwan^48^), this difference was consistent with our evidence of small study effects, whereby smaller studies tended to estimate lower incidence rates. Together with the high levels of statistical heterogeneity observed in our meta-analyses, our results suggest that methodological variation might partially obscure true heterogeneity in the incidence of psychotic disorders. Nonetheless, as previously established, we found strong evidence of higher incidences of all first episode psychosis and non-affective psychotic disorders in men and ethnic minority groups, with less evidence of such differences for affective psychotic disorders.

The strength of our study is that our search strategy was inclusive and based on a previously used strategy with good reliability.^18^ We searched multiple databases without restriction by place or language of publication. Although individual studies might have been missed, given the size of our yield we consider it unlikely that these missing data would have substantially altered our main conclusions.

One limitation of our Article was that some citations provided incidence estimates from multiple catchment areas (notably Jongsma and colleagues, 2018),^55^ which we included as separate estimates in meta-analyses. We acknowledge this inclusion might have conservatively biased SEs around effect sizes. Nonetheless, it would not have affected our observation of substantial interestimate heterogeneity in incidence, which was the primary focus of our Article. Future studies should consider adopting individual-participant data approaches, which account for clustering by design.^56^ We used a previously published, clinician-informed algorithm to group estimates into major psychotic disorder categories.^18^ However, for non-affective disorders particularly, the use of this algorithm led to the categorisation of studies that used several overlapping diagnostic outcomes (appendix pp 33–34), which might have contributed to heterogeneity. Although our quality assessment tool was based on epidemiological good practice, we acknowledge it might have been skewed towards first-contact studies given it is not feasible to assess some criteria (ie, blinding) in register-based designs. Despite this, our quality assessment aided in assessing the gaps in the published literature.

The most recent systematic review and meta-analysis of all psychotic disorders^19^ identified substantially fewer citations (N=33) than our Article and provided no assessment or investigation of heterogeneity, despite similar inclusion criteria and time frames. The estimates of our more comprehensive review are aligned: we found a pooled estimate of non-affective disorders of 18·7 per 100 000 person-years (95% CI 14·8–23·7) and of affective disorders of 4·8 (3·3–6·9) compared with their estimates of 22·5 (16·5–28·5) for non-affective and 7·1 (1·4–12·2) for affective disorders.^19^ Our findings on the excess of psychoses in men were nuanced: the overall excess found in both reviews appears to be primarily driven by an excess in non-affective disorders in line with other meta-analytic evidence.^27^, ^28^

The median incidence of schizophrenia in our Article (21·7 per 100 000 person-years [IQR 5·6–52·0]) was higher than in the last major systematic review^17^ on this topic by McGrath and colleagues (15·2 [7·7–43·0]), with greater variation around these estimates. The only systematic review^57^ pertaining to mood disorders solely synthesised incidence of major depressive disorder and as such is not directly comparable to the present Article. The excess risk of (non-affective) psychotic disorders in migrants and their descendants is long-established,^58^ well-reported,^20^, ^29^ and covered elaborately in one publication.^30^

The present Article presents a varied epidemiological landscape, which partly appears to reflect methodological differences in study design. We found substantial heterogeneity both within and between study designs, with incidences of non-affective disorders, schizophrenia, and bipolar disorder higher in registry-based studies than in first contact studies. Different study designs were more common for different outcomes; for instance, a large proportion of schizophrenia studies were population registers, potentially contributing to this pooled estimate being higher than the pooled estimate of non-affective disorders (a broader category). Although individual studies^36^, ^37^ have done direct comparisons between different study designs, to our knowledge this study is the first systematic review to have investigated such differences. From a public mental health perspective, our results highlight the importance of parsing out potentially causally-relevant signals in geographical variance in incidence from noise generated through varying study designs used in different settings; individual studies^16^, ^55^, ^59^ that have done so suggest substantive variation in the global burden of psychotic disorders remains.

Nevertheless, more research is required to understand heterogeneity in incidence produced by different study designs. One possible explanation is that register-based studies primarily (though not exclusively) originate from Scandinavian countries, and higher incidences might indicate an association between latitude and psychotic disorders (which is well-reported, but poorly understood).^31^ Alternatively, although registry-based studies might ascertain new cases of psychotic disorder across an entire (usually secondary and tertiary) health-care system, not limited to contact with mental health providers, they also rely heavily on diagnoses made in clinical practice. Although such diagnoses are reliable,^60^ first-contact studies are often able to include standardised diagnostic assessments, which might reduce the number of false positives, leading to lower reported incidence. Small study effects are not necessarily due to publication bias^41^ and in our Article are consistent with the possibility of lower incidence rates reported in first contact designs; registry-based or insurance database-based studies tended to include a larger number of cases (table). However, sensitivity analyses (appendix p 32) suggest some within-type small study effects remained, which might reflect real variance between for instance urban (where a large number of cases accrue) and rural areas. In this Article, we were unable to assess effects of urbanicity, latitude, or other socioeconomic variables due to the preponderance of country-wide estimates for which no meaningful values could be assigned.

The geographical spread of studies in this Article remained mostly limited to Europe, Northern America, or Australia. One public health implication of our findings is the continued dearth of evidence outside of these settings, which might have profound consequences; for example, a cross-sectional study^16^ suggested the well established link between urbanicity and psychosis might not apply in LMICs. To fully understand and provide effective public mental health responses to the global burden of psychotic disorders, we will require methodologically-rigorous and culturally-appropriate epidemiological studies to delineate the incidence of psychotic disorders in a broader range of settings than has thus far been considered.

Finally, our findings also suggest that developing international guidelines for investigation of the incidence of psychotic disorders in different settings could help minimise methodological heterogeneity in the reporting of psychosis incidence across the globe.
