# Supplementary material for: International incidence of psychotic disorders, 2002–17: a systematic review and meta-analysis
Source: Lancet Public Health. 2019 May 1;4(5):e229–44. doi: 10.1016/S2468-2667(19)30056-8 (PMC6693560; doi:10.1016/S2468-2667(19)30056-8)
Supplement: Supplementary appendix [file mmc1.pdf]

THE LANCET
Public Health

Supplementary appendix

This appendix formed part of the original submission and has been peer reviewed. We post it as supplied by the authors.

Supplement to: Jongsma HE, Turner C, Kirkbride JB , Jones PB. International incidence of psychotic disorders, 2002–17: a systematic review and meta-analysis. *Lancet Public Health* 2019; **4:** e229–44.

Supplemental Material

List of Supplemental Tables

Supplemental Table 1: PRISMA Checklist 2
Supplemental Table 2: Search strategy as used in Web of Science 4
Supplemental Table 3: Diagnostic codes by diagnostic manual. 5
Supplemental Table 4: Quality Assessment 5
Supplemental Table 5: Detailed study quality by citation 6
Supplemental Table 6: Details of citations covering young people (<40 years) 9
Supplemental Table 7: Details of citations covering comorbid population groups 11
Supplemental Table 8: Details of citations covering the military or army veterans 11
Supplemental Table 9: Meta-regression all FEP 26
Supplemental Table 10: Meta-regression non-affective disorders 26
Supplemental Table 11: Meta-regression schizophrenia 27
Supplemental Table 12: Meta-regression affective psychotic disorders 27
Supplemental Table 13: Meta-regression bipolar disorder with psychosis 28
Supplemental Table 14: Egger's test for small study effects 29
Supplemental Table 15: Sensitivity analyses: Egger's test by study type 32
Supplemental Table 16: Exact diagnoses included in the non-affective psychotic disorders category. 33

List of Supplemental Figures

Supplemental Figure 1: Incidence of all psychotic disorders by countries' economic classification 13
Supplemental Figure 2: Incidence of non-affective psychotic disorders by countries' economic classification... 14
Supplemental Figure 3: Incidence of schizophrenia by countries' economic classification. 15
Supplemental Figure 4: Incidence of affective disorders by countries' economic classification 16
Supplemental Figure 5: Incidence rate ratios (men : women) by diagnostic outcome 17
Supplemental Figure 6: Incidence rate ratio (minority : majority) by diagnostic outcome 18
Supplemental Figure 7: Incidence rate ratios by ethnic group, all psychotic disorders. 19
Supplemental Figure 8: Incidence rate ratios by ethnic group, non-affective disorders 20
Supplemental Figure 9: Incidence rate ratios by ethnic group, schizophrenia 21
Supplemental Figure 10: Incidence by age group, all psychotic disorders 22
Supplemental Figure 11: Incidence by age group, non-affective disorders 23
Supplemental Figure 12: Incidence by age group, schizophrenia 24
Supplemental Figure 10: Funnel plots for all FEP 30
Supplemental Figure 11: Funnel plots of non-affective disorders 30
Supplemental Figure 12: Funnel plots of schizophrenia 31
Supplemental Figure 13: Funnel plots of affective psychotic disorders 31
Supplemental Figure 14: Funnel plots of bipolar disorder 32

Supplemental Methods

Below is the full PRISMA checklist (Supplemental Table 1).

Supplemental Table 1: PRISMA Checklist

Section/topic	#	Checklist item	Reported on page #
**Title**			
Title	1	Identify the report as a systematic review, meta-analysis, or both.	1
**Abstract**			
Structured summary	2	Provide a structured summary including, as applicable: background; objectives; data sources; study eligibility criteria, participants, and interventions; study appraisal and synthesis methods; results; limitations; conclusions and implications of key findings; systematic review registration number.	2 – abstract
**Introduction**			
Rationale	3	Describe the rationale for the review in the context of what is already known.	3 - introduction
Objectives	4	Provide an explicit statement of questions being addressed with reference to participants, interventions, comparisons, outcomes, and study design (PICOS).	3 - introduction
**Methods**			
Protocol and registration	5	Indicate if a review protocol exists, if and where it can be accessed (e.g., Web address), and, if available, provide registration information including registration number.	3 - methods
Eligibility criteria	6	Specify study characteristics (e.g., PICOS, length of follow-up) and report characteristics (e.g., years considered, language, publication status) used as criteria for eligibility, giving rationale.	3 – methods
Information sources	7	Describe all information sources (e.g., databases with dates of coverage, contact with study authors to identify additional studies) in the search and date last searched.	3 - methods
Search	8	Present full electronic search strategy for at least one database, including any limits used, such that it could be repeated.	Supplemental Table 2
Study selection	9	State the process for selecting studies (i.e., screening, eligibility, included in systematic review, and, if applicable, included in the meta-analysis).	3 - methods
Data collection process	10	Describe method of data extraction from reports (e.g., piloted forms, independently, in duplicate) and any processes for obtaining and confirming data from investigators.	4 - methods
Data items	11	List and define all variables for which data were sought (e.g., PICOS, funding sources) and any assumptions and simplifications made.	4 – methods. Full spreadsheet available online
Risk of bias in individual studies	12	Describe methods used for assessing risk of bias of individual studies (including specification of whether this was done at the study or outcome level), and how this information is to be used in any data synthesis.	4 - methods
Summary measures	13	State the principal summary measures (e.g., risk ratio, difference in means).	4 - methods
Synthesis of results	14	Describe the methods of handling data and combining results of studies, if done, including measures of consistency (e.g., I^2) for each meta-analysis.	4 - methods
Risk of bias across studies	15	Specify any assessment of risk of bias that may affect the cumulative evidence (e.g., publication bias, selective reporting within	4 - methods

		studies).	
Additional analyses	16	Describe methods of additional analyses (e.g., sensitivity or subgroup analyses, meta-regression), if done, indicating which were pre-specified.	4 – methods,<br>6- results
**Results**			
Study selection	17	Give numbers of studies screened, assessed for eligibility, and included in the review, with reasons for exclusions at each stage, ideally with a flow diagram.	Figure 1
Study characteristics	18	For each study, present characteristics for which data were extracted (e.g., study size, PICOS, follow-up period) and provide the citations.	Table 1
Risk of bias within studies	19	Present data on risk of bias of each study and, if available, any outcome level assessment (see item 12).	Supplemental Table 5
Results of individual studies	20	For all outcomes considered (benefits or harms), present, for each study: (a) simple summary data for each intervention group (b) effect estimates and confidence intervals, ideally with a forest plot.	Figures 2-6 / online spreadsheet
Synthesis of results	21	Present results of each meta-analysis done, including confidence intervals and measures of consistency.	5 - Results Figures 2-6
Risk of bias across studies	22	Present results of any assessment of risk of bias across studies (see Item 15).	6, Supplemental Tables 14/15
Additional analysis	23	Give results of additional analyses, if done (e.g., sensitivity or subgroup analyses, meta-regression [see Item 16]).	6, Supplemental Tables 9-13
**Discussion**			
Summary of evidence	24	Summarize the main findings including the strength of evidence for each main outcome; consider their relevance to key groups (e.g., healthcare providers, users, and policy makers).	6 - Discussion
Limitations	25	Discuss limitations at study and outcome level (e.g., risk of bias), and at review-level (e.g., incomplete retrieval of identified research, reporting bias).	7 - discussion
Conclusions	26	Provide a general interpretation of the results in the context of other evidence, and implications for future research.	7/8 - discussion
**FUNDING**			
Funding	27	Describe sources of funding for the systematic review and other support (e.g., supply of data); role of funders for the systematic review.	4 – methods and acknowledgements

From: Moher D, Liberati A, Tetzlaff J, Altman DG, The PRISMA Group (2009). Preferred Reporting Items for Systematic Reviews and Meta-Analyses: The PRISMA Statement. PLoS Med 6(7): e1000097. doi:10.1371/journal.pmed1000097

Below are the search strategies as used in the PubMed and Web of Science databases.

PubMed

((((((((inciden*[Title/Abstract]) OR epidemiolog*[Title/Abstract])) OR ((((((episod*[Title/Abstract]) OR contact*[Title/Abstract]) OR admission*[Title/Abstract]) OR admit*[Title/Abstract])) AND (((first*[Title/Abstract]) OR 1st[Title/Abstract]) OR hospital*[Title/Abstract]))) OR ((case[Title/Abstract]) AND register*[Title/Abstract])) OR case control*[Title/Abstract]) OR ((((prospectiv*[Title/Abstract]) OR population*[Title/Abstract]) OR communit*[Title/Abstract]) OR survey*[Title/Abstract]))) AND (((((((((schizo*[Title/Abstract]) OR (((psychotic[Title/Abstract]) OR psychosis[Title/Abstract]) OR psychoses[Title/Abstract])) OR bipolar disorder*[Title/Abstract]) OR delusion* disorder[Title/Abstract]) OR (((((illness*[Title/Abstract]) OR disorder*[Title/Abstract])) AND mental[Title/Abstract]) AND (((severe[Title/Abstract]) OR serious[Title/Abstract]) OR chronic[Title/Abstract]))) OR SMI[Title/Abstract]) OR mani* depressi*[Title/Abstract]) OR chronic psychosis) OR schizoaffective disorder) AND ("2002/01/01"[PDat] : "2017/12/31"[PDat])

Web of science

Supplemental Table 2: Search strategy as used in Web of Science

**#19**	#18 AND #1<br>*DocType=All document types; Language=All languages;*
**#18**	#17 AND #11<br>*DocType=All document types; Language=All languages;*
**#17**	#16 OR #15 OR #14 OR #13 OR #12<br>*DocType=All document types; Language=All languages;*
**#16**	TI=(prospectiv* or population* or communit* or survey*)<br>*DocType=All document types; Language=All languages;*
**#15**	TI=(case control*)<br>*DocType=All document types; Language=All languages;*
**#14**	TI=(case AND register)<br>*DocType=All document types; Language=All languages;*
**#13**	TI=(inciden* OR epidemiolog*)<br>*DocType=All document types; Language=All languages;*
**#12**	TI=((first* OR 1st OR hospital*) AND (episod* OR contact* OR admission* OR admit*))<br>*DocType=All document types; Language=All languages;*
**#11**	#10 OR #9 OR #8 OR #7 OR #6 OR #5 OR #4 OR #3 OR #2<br>*DocType=All document types; Language=All languages;*
**#10**	TI=(schizoaf* disorder)<br>*DocType=All document types; Language=All languages;*
**#9**	TI=(mani* depressi*)<br>*DocType=All document types; Language=All languages;*
**#8**	TS=psychosis<br>*DocType=All document types; Language=All languages;*
**#7**	TI=(SMI)<br>*DocType=All document types; Language=All languages;*
**#6**	TI=((sever OR serious OR chronic) AND mental AND (illness* OR disorder*))<br>*DocType=All document types; Language=All languages;*
**#5**	TI=(delusion* disorder)<br>*DocType=All document types; Language=All languages;*
**#4**	TI=(bipolar disorder*)<br>*DocType=All document types; Language=All languages;*
**#3**	TI=(psychotic OR psychosis OR psychoses)<br>*DocType=All document types; Language=All languages;*
**#2**	TI=(schizo*)<br>*DocType=All document types; Language=All languages;*
**#1**	PY=(2002-2017)<br>*DocType=All document types; Language=All languages;*

The below Supplemental Table (3) indicates which diagnostic codes were used for which outcome, per diagnostic manual.

Supplemental Table 3: Diagnostic codes by diagnostic manual.

Diagnostic category	ICD-8	ICD-9	ICD-10	DSM-III	DSM-IV
All psychotic disorders	291, 295-299	292.1,293.1 295-298	F20-33	--[1]	292.11/.12, 295-299
Non-affective disorders *	295, 297-299	295,297,298.1-298.9	F20-29	--[1]	295,297,298
Schizophrenia	295	295	F20	295	295.10/.20/.30/60.90
Affective psychotic disorders	296	296, 298.0	F30-33	--[1]	296.x4
Bipolar disorder with psychosis	--[2]	296.0-296.6	F30-31	296.44,296.44I, 296.54C,296.54I, 296.64C, 296.64I	296.04/.44/.54/.64/
Psychotic depression	--[2]	298.0	F32-33	296.34	296.24/.34
Substance-induced psychosis	291	292.1, 293.1	F1X.5	--[1]	292.11,292.12

* The non-affective category in practice included anything that was broader than schizophrenia and did not include any affective disorders. See Supplemental Table 16.

[1] It was not possible to derive these diagnostic categories from the data included in the review

[2] ICD-8 did not differentiate the affective psychoses clearly.

Where citations reported overlapping data from the same study or population, we used the following criteria to determine inclusion in analyses:

(i) data most relevant to the specific outcome and/or exposure under investigation;
(ii) data presented with a corresponding standard error;
(iii) data most closely related to entry criteria for the individual analysis;
(iv) published data;
(v) citations published in the highest impact journal according to Clarivate Journal Citation Reports 2017.

Quality of yield was assessed using the criteria detailed in Supplemental Table 4 below.

Supplemental Table 4: Quality Assessment

Quality criterion	Explanation
Defined catchment area	A clearly defined catchment area was reported. This could be a whole population cohort, or existing boundaries of a municipality or clinical service.
Accurate reporting and reliable source of denominator data	Denominator data was traceable to a reliable source, such as official government statistics.
Population-based case-finding	Cases were identified across the whole population of interest.
Blinding of clinician to demographic variables	In determining a diagnosis, the clinician was blinded to cases' demographic characteristics, such as age, sex and ethnicity
Inclusion criteria clearly stated	Inclusion criteria for the study are listed in such a way that it the study would potentially be replicable.
Leakage study	Leakage methodology was employed to minimise case ascertainment bias. This tends to involve trawling of records of services that are not part of the core study, such as A&E administrative data and police records.

Supplemental results

Study quality

Below are details on study quality by citation. Study quality was scored on seven criteria: having a defined catchment area, reporting an accurate and reliable source of denominator data, conducting population-based case-finding, using standardized research diagnoses, blinding of the clinician to demographic variables, a clear listing of inclusion criteria and conducting a leakage study. The total quality score was used in meta-regression.

Supplemental Table 5: Detailed study quality by citation

First Author (year)	Defined catch-ment area	Accurate denomi-nator	Popu-lation based case-finding	Standar-dised research diagnosis	Blinding to demo-graphic variables	Inclusion criteria	Leakage study	Total Quality Score
Tsuchiya (2002)	1	1	1	0	0	1	0	4
Hanoeman (2002)	1	1	1	0	0	1	0	4
Selten (2002)	1	1	1	0	0	1	0	4
Baldwin (2002)	1	1	1	1	0	1	0	5
Scully (2002)	1	1	1	1	0	1	0	5
Boydell (2003)	1	1	1	1	1	1	1	7
Smith (2003)	1	1	0	0	0	0	0	2
Singh (2003)	1	0	0	0	0	1	0	2
Selten (2003)	1	1	0	0	0	1	0	3
Cantor-Graae (2003)	1	1	1	0	0	1	0	4
Baldwin (2003)	1	1	1	1	0	1	0	5
Proctor (2004)	1	0	0	0	0	0	0	1
Sipos (2004)	1	1	1	0	0	1	0	4
Chien (2004)	1	1	1	0	0	1	0	4
Boydell (2004)	1	0	1	1	1	1	1	6
Veen (2004)	1	1	1	1	1	1	0	6
Singh (2004)	1	1	1	1	0	1	1	6
Sailas (2005)	1	1	0	0	0	0	0	2
Harris (2005)	1	0	1	0	0	1	0	3
Sundquist (2005)	1	1	1	0	0	1	0	4
Nager (2005)	1	1	1	0	0	1	0	4
Laursen (2005)	1	1	1	0	0	1	0	4
Selten (2005)	1	1	1	0	0	1	0	4
Nixon (2005a)	1	0	0	1	1	0	1	4
Qin (2005	1	1	1	0	0	1	0	4
Allardyce (2005)	1	1	1	0	0	1	0	4
Cantor-Graae (2005)	1	1	1	0	1	1	0	5
Baldwin (2005)	1	1	1	1	0	1	0	5
Kennedy (2005a)	1	1	1	1	0	1	1	6
Kennedy (2005b)	1	1	1	1	0	1	1	6
Lloyd (2005)	1	1	1	1	1	1	1	7
Leao (2006)	1	1	0	0	0	1	0	3
Bray (2006)	1	1	0	0	0	1	0	3
Payne (2006)	1	1	0	0	0	1	0	3
Drukker (2006)	1	1	0	0	0	1	0	3
Turner (2006)	1	1	1	0	0	0	0	3
Mahmmood (2006)	1	0	0	1	0	1	0	3
Westman (2006)	1	1	1	0	1	0	0	4
Munk-Olsen (2006)	1	1	1	0	0	1	0	4
Smith (2006)	1	1	0	1	1	1	0	5
Amminger (2006)	1	1	1	1	0	1	0	5
Veling (2006)	1	1	1	1	0.5[1]	1	0	5.5
Morgan (2006)	1	1	1	1	0	1	1	6
Fearon (2006)	1	1	1	1	0	1	1	6
Gould (2006)	1	1	1	1	0	1	1	6
Kirkbride (2006)	1	1	1	1	1	1	1	7
Zipursky (2006)	1	1	1	1	1	1	1	7
Li (2007)	1	1	1	0	0	0	0	3
Schimmelmann (2007)	1	1	0	0	0	1	0	3
Laursen (2007)	1	1	1	0	0	1	0	4
Ajdacic-Gross (2007)	1	1	1	0	0	1	0	4
Andersen (2007)	1	1	1	0	0	1	0	4
Harlow (2007)	1	1	1	0	0	1	0	4
Juvonen (2007)	1	1	1	0	0	1	0	4
Cantor-Graae (2007a)	1	1	1	0	0	1	0	4
Cantor-Graae (2007b)	1	1	1	0	0	1	0	4
Leao (2007)	1	1	1	0	1	1	0	5
Kikbride (2007a)	1	1	1	1	0	1	1	6
Menezes (2007)	1	1	1	1	0	1	1	6
Kirkbride (2007b)	1	1	1	1	0	1	1	6
Stain (2008)	1	0	0	0	0	1	0	2

Boonstra (2008)	1	0	0	0	0	1	0	2
Crebbin (2008)	1	0	0	0	0	1	1	3
Farquhar (2008)	1	1	0.5[1]	0	0	1	0	3.5
Pelayo-Teran (2008)	1	0	1	1	0	1	0	4
Castagnini (2008	1	1	1	0	0	1	0	4
Burns (2008)	1	0	1	0	1	1	0	4
Weiser (2008)	1	1	1	0	0	1	0	4
Veling (2008)	1	1	1	1	0.5[1]	1	0	5.5
Kirkbride (2008a)	1	1	1	1	1	1	0	6
Kirkbride (2008b)	1	1	1	1	1	1	0	6
Coid (2008)	1	1	1	1	1	1	1	7
Grant (2009)	0	0	1	0	0	1	0	2
Crebbin (2009)	1	0	0	0	0	1	0	2
Bih (2009)	1	1	1	0	0	1	0	4
Corcoran (2009)	1	1	1	0	0	1	0	4
Osby (2009)	1	1	1	0	0	1	0	4
Valdimarsdottir (2009)	1	1	1	0	0	1	0	4
Harlap (2009)	1	1	1	0	0	1	0	4
Reay (2009)	1	1	0	1	0	1	0	4
Norredam (2009)	1	1	1	0	0	1	0	4
Bogren (2009	1	1	1	0.5[1]	0	1	0	4.5
Kirkbride (2009)	1	1	1	1	0	1	1	6
Coid (2009)	ERRATUM ONLY TO COID (2008)							
Cheng (2010)	1	1	1	0	0	1	0	4
Healy (2010)	1	1	0.5[1]	0	0	1	0	3.5
Bogren (2010)	1	1	1	0	0	1	0	4
Zammit (2010)	1	1	1	0	0	1	0	4
Tseng (2010)	1	1	1	0	0	1	0	4
Zandi (2010)	1	1	1	0	0	1	0	4
Norredam (2010)	1	1	1	0	0	1	0	4
Goodman (2011)	1	1	0	0	0	1	0	3
Cowan (2011)	1	1	0	0	0	1	0	3
Harris (2011)	1	1	0.5[1]	0	0	1	0	3.5
Jorgensen (2011)	1	1	1	0	0	1	0	4
Cheng (2011)	1	1	1	0	0	1	0	4
Kleinhaus (2011)	1	1	1	0	0	1	0	4
Benros (2011)	1	1	1	0	0	1	0	4
Salokangas (2011)	1	1	1	0	0	1	0	4
Schofield (2011)	1	1	1	0	0	1	0	4
Veling (2011)	1	1	1	1	0.5[1]	1	0	5.5
Callaghan (2012)	1	1	0	0	0	0	0	2
Anderson (2012)	1	0	0	0	0	1	0	2
Manrique-Garcia (2012)	1	1	0	0	0	1	0	3
Turola (2012)	1	1	0	0	0	1	0	3
Werbeloff (2012)	1	1	1	0	0	1	0	4
Nosarti (2012)	1	1	1	0	0	1	0	4
Gigantesco (2012)	1	1	0	1	0	1	0	4
Tarricone (2012)	1	1	0	1	0	1	1	5
Kirkbride (2012)	1	1	1	1	0	1	0	5
Hung (2013)	1	1	0	0	0	1	0	3
Peritogiannis (2013)	1	0	1	0.5[1]	1	0	0	3.5
Sutterland (2013)	1	1	1	0	0	0	1	4
Cantor-Graae (2013)	1	1	1	0	0	1	0	4
Kroon (2013)	1	1	1	0	0	1	0	4
Castagnini (2013)	1	1	1	0	0	1	0	4
Hardoon (2013)	1	1	1	0	0	1	0	4
Weibell (2013)	1	1	1	1	0	1	0	5
Cocchi (2014)	0	0	0	0	0	1	0	1
Tortelli (2014)	1	1	0	0	0	1	0	3
Hogerzeil (2014)	1	1	1	0	0	1	0	4
Pedersen (2014)	1	1	1	0	0	1	0	4
Sorensen (2014)	1	1	1	0	0	1	0	4
Munk-Olsen (2014)	1	1	1	0	0	1	0	4
Szoke (2014)	1	1	0	0	0	1	1	4
Bhavsar (2014)	1	1	0	1	0	1	1	5
Omer (2014)	1	1	1	1	0	1	0	5
Lasalvia (2014)	1	1	0	1	0	1	1	5
Veling (2014)	1	1	1	1	0.5[1]	1	0	5.5
Kirkbride (2014)	1	1	1	1	0	1	1	6
Anderson (2015)	1	0	1	0	0	1	0	3

Paksarian (2015a)	1	1	1	0	0	1	0	4
Sorensen (2015)	1	1	1	0	0	1	0	4
Paksarian (2015b)	1	1	1	0	0	1	0	4
Soderlund (2015)	1	1	1	0	0	1	0	4
Medici (2015)	1	1	1	0	0	1	0	4
Carlborg (2015)	1	1	1	0	0	1	0	4
Tsai (2016)	1	1	0	0	0	1	0	3
Chen (2016)	1	1	1	0	0	1	0	4
Latvala (2016)	1	1	1	0	0	1	0	4
Jensen (2016)	1	1	1	0	0	1	0	4
Kuhl (2016)	1	1	1	0	0	1	0	4
Filatova (2016)	1	1	1	0	0	1	0	4
Chiang (2016)	1	1	1	0	0	1	0	4
Nielsen (2016)	1	1	1	0	0	1	0	4
Kendler (2016)	1	1	1	0	0	1	0	4
Levine (2016a)	1	1	1	0	0	1	0	4
Levine (2016b)	1	1	1	0	0	1	0	4
Vassos (2016)	1	1	1	0	0	1	0	4
Sorensen (2016)	1	1	1	0	0	1	0	4
Hollander (2016)	1	1	1	0	0	1	0	4
O'Donoghue (2016)	1	1	1	1	0	1	0	5
Morgan (2016)	1	0	1	1	0	1	1	5
Tarricone (2016)	1	1	0	1	0	1	1	5
Szoke (2016)	1	1	1	0	0	1	1	5
Mule (2016)	1	1	1	1	0	1	1	6
Ramsey (2017)	1	0	0	0	0	1	0	2
Okkels (2017)	1	1	0	0	0	1	0	3
Vikstrom (2017)	1	1	0	0	0	1	0	3
Wang (2017)	1	1	0	0	0	1	0	3
Lin (2017)	1	1	0.5[1]	0	0	1	0	3.5
Marrie (2017a)	1	1	0.5[1]	0	0	1	0	3.5
Marrie (2017b)	1	1	0.5[1]	0	0	1	0	3.5
Hogerzeil (2017)	1	1	1	0	0	1	0	4
Hoeffding (2017)	1	1	1	0	0	1	0	4
Kim (2017)	1	1	1	0	0	1	0	4
Markkula (2017)	1	1	1	0	0	1	0	4
Nielsen (2017)	1	1	1	0	0	1	0	4
Schofield (2017a)	1	1	1	0	0	1	0	4
Schofield (2017b)	1	1	1	0	0	1	0	4
Simon (2017)	1	1	1	0	0	1	0	4
Kirkbride (2017a)	1	1	1	1	0	1	0	5
Kirkbride (2017b)	1	1	1	1	0	1	0	5
Nyberg (2018)	1	1	1	0	0	1	0	4
Barghadouch (2018)	1	1	1	0	0	1	0	4
Richardson (2018)	1	1	1	1	0	1	0	5
Jongsma (2018)	1	1	1	1	0	1	0.5[1]	5.5

[1] A score of 0.5 indicates that this item was met for part of the study, but not the whole study. For instance, in Jongsma et al (2018) a leakages study was carried out in some, but not all catchment areas.

Incidence of psychotic disorders in young people (<40 years old)

Twenty-six citations only described the incidence of psychotic disorder in young people (Supplemental Table 6). This was defined as an upper age limit of 40 years or lower. Seven of these citations included data derived from England, five were derived from Sweden, three each from Australia, Canada and Denmark, two from Israel and one each from Finland, the Netherlands and Switzerland. These studies are described in Supplemental Table 5 below.

The incidence of all psychotic disorders was reported by nine studies [1–9], and varied from 11.78 per 100,000 person-years in one Australian study[1] to 167.0 in men in a second Australian study[2]. The incidence of non-affective disorders was reported in 14 citations[6,7,10–22], and ranged from 21.2 per 100,000 person-years reported in Israel[14] to 138.1 in men refugees in Denmark[13]. The incidence of schizophrenia was reported in 8 citations[6–8,11,21–24], and ranged from 17.3 (95%CI: 15.6-19.2) in England[6,7] to 119.6 (95%CI: 107.4-132.4) per 100,000 person-years in men in Canada[23]. Affective disorders were reported less frequently (three citations[11,15,21]) and varied from 11.3 in Sweden[11] to 15.6 per 100,000 person-years in Denmark[21]. The incidence of bipolar disorder is reported by five citations[6–8,19,22] and varied from 3.2 (95%CI: 2.5-4.1) per 100,000 person-years in England[6,7] to 16.2 in Denmark[19]. The incidence of psychotic depression is reported by four citations[6,8,19,25] and varied from

0.9 (95%CI: 0.6-1.5) in England[6,7] to 10.5 per 100,000 person-years in Finland[8]. Three citations[6,7,26] reported some other form of psychotic disorders. Two citations covering the Schizophrenia and other Psychoses in East Anglia (SEPEA) study reported an incidence of substance induced psychosis of 1.5 (95%CI: 1.0-2.1) per 100,000 person-years[6,7], and a further citation reported an incidence of clinically relevant HoNOS scores of between 11.4 and 25.2 per 100,000 person-years[26].

Supplemental Table 6: Details of citations covering young people (<40 years)

First author (year)	Country	Age range	Outcomes included	Incidence rate per 100,000 person-years (95%CI)
Harris (2005)	Australia	13-25	All psychotic disorders	11.78[1]
Leao (2007)	Sweden	16-34	 Non-affective disorders<br>Affective disorders	Not reported. Hazard ratio (HR) for men:<br>1.40 (95%CI: 1.31-1.59)<br>0.64 (95%CI: 0.61-0.69)
Leao (2006)	Sweden	20-39	Non-affective disorders	Age-standardised only. Varied from 31 in Swedish women to 123 in 2nd generation Finnish men.
Bray (2006)	Canada	14-24	Schizophrenia	Increase between 1989 and 1998.<br>Women: from 77.1 (90%CI: 42.1-137.7) to 89.9 (90%CI:80.1-100.1).<br>Men: from 66.6 (90%CI: 38.8-113.3) to 119.6 (95%CI: 107.4-132.4)
Amminger (2006)	Australia	15-29	All psychotic disorders	Men: 167.0<br>Women: 81.0
Ajdacic-Gross (2007)	Switzerland	15-29	Non-affective disorders	Not reported[2]
Stain (2008)	Australia	10-25	HoNOS scores	Coastal: 11.4<br>Remote: 21.4<br>Rural: 25.2[1]
Corcoran (2009)	Israel	0-33	Non-affective disorders	21.2
Cheng (2010)	England	17-35	All psychotic disorders	50.0 (44.5-56.2)
Cheng (2011)	England	16-35	All psychotic disorders	50.0 (44.5-56.2)
Kleinhaus (2011)	Israel	0-39	Non-affective disorders	All: 24.7<br>Men: 30.0<br>Women: 19.1
Nosarti (2012)	Sweden	16-29	Non-affective disorders<br>Bipolar disorder	7.0<br>2.0
Kirkbride (2013)	England	16-35	All psychotic disorders	All: 42.5<br>Men: 49.6<br>Women: 28.4
Anderson (2012)	Canada	14-25	Non-affective disorders	All: 43.6<br>Men: 84.4<br>Women: 33.1
Cantor-Graae (2013)	Denmark	0-40	Non-affective disorders<br>Schizophrenia<br>Affective disorders	52.8<br>39.2<br>15.9
Bhavsar (2014)	England	16-35	Schizophrenia	54.6
Paksarian (2015)	Denmark	15-39	Non-affective disorders<br>Schizophrenia<br>Bipolar disorder	77.2<br>43.4<br>16.2
Soderlund (2015)	Sweden	18-30	Non-affective disorders<br>Schizophrenia<br>Affective disorders	71.7<br>30.3<br>11.3
Anderson (2015)	Canada	14-40	Non-affective disorders	Majority population: 55.6 (54.9-56.4)<br>Migrants: 51.7 (49.2-54.4)<br>Refugees: 72.8 (67.1-78.9)
Filatova (2016)	Finland	0-27	All psychotic disorders<br>Schizophrenia<br>Bipolar disorder<br>Psychotic depression	93.9<br>29.3<br>9.2<br>10.5
Hollander (2016)	Sweden	14-40	Non-affective disorders	Overall: 41.9<br>Majority population: 38.5<br>Migrants: 80.4<br>Refugees: 126.4
Kirkbride (2016)	England	16-35	All psychotic disorders	All: 34.0<br>Men: 44.5<br>Women: 23.0
Kirkbride (2017)	England	16-35	All psychotic disorders<br>Non-affective disorders<br>Schizophrenia<br>Bipolar disorder<br>Psychotic depression<br>Substance-induced psychosis	34.0 (31.5-36.6)<br>28.3<br>17.3 (15.6-19.2)<br>3.2 (2.5-4.1)<br>0.9 (0.6-1.5)<br>1.5 (1.0-2.1)

Richardson (2018)	England	16-35	See 206	See 206
Barghadouch (2018)	Denmark	18-24	Non-affective disorders	All refugees: 95.8<br>Men refugees: 138.1<br>Women refugees: 44.5
Selten (2002)	The Netherlands	15-39	Schizophrenia	All: 19.1<br>Men: 23.5<br>Women 14.4

[1] Based on approximate denominator
[2] Reported as 3-year moving averages in graphs only.

Incidence of psychotic disorders in special population groups

Twelve citations reported the incidence of psychotic disorders in a population group with a pre-exiting comorbidity and compared it with a general population cohort[27–38]. These studies are summarised in Supplemental Table 7 below. These citations reported data from Taiwan (n=5), Denmark (n=4), Canada (n=2) and Finland (n=1). Most pre-existing medical conditions investigated increased the risk for a psychotic disorder. The highest relative increase was reported in Danish patients with comorbid substance use (Hazard Ratio [HR] of schizophrenia: 6.04, 95%CI: 5.84-6.26)[38]. The only comorbidity associated with a lower risk of any disorder was Type-1 diabetes in Finland (relative risk [RR] for non-affective disorders: 0.38, 95%CI: 0.25-0.57)[28]. Please note that in the citations originating from the Taiwanese health insurance database it was impossible to differentiate between bipolar disorder with and without psychosis[32–35].

Supplemental Table 7: Details of citations covering comorbid population groups

First author (year)	Country	Comorbidity	Outcome of interest	Relative risk (RR) / Incidence Rate Ratio (IRR) / Hazard Ratio (HR)
Qin (2005)	Denmark	Epilepsy	Non-affective disorders<br>Schizophrenia	RR: 2.93 (95%CI: 2.69-3.20)<br>RR: 2.48 (95%CI: 2.20-2.80)
Juvonen (2007)	Finland	Type-1 diabetes	Non-affective disorders	RR: 0.38 (95%CI: 0.25-0.57)
Benros (2011)	Denmark	Autoimmune disorders	Non-affective disorders	IRR: 1.29 (95%CI: 1.15-1.41)
Hung (2013)	Taiwan	Breast cancer	Bipolar disorder	IRR: 2.06 (95%CI: 1.37-3.15)
Chen (2015)	Taiwan	Prostate cancer	Bipolar disorder	IRR: 1.84 (95%CI: 1.25-2.74)
Tsai (2016)	Taiwan	COPD	Bipolar disorder	HR: 2.43 (95%CI: 1.65-3.58)
Lin (2017)	Taiwan	Scabies	Bipolar disorder	HR: 1.86 (95%CI: 1.36-2.54)
Marrie (2017a)	Canada	Immune disorders	Schizophrenia<br>Bipolar disorder	IRR: 1.32 (95%CI: 1.03-1.69)<br>IRR: 1.68 (95%CI: 1.52-1.85)
Marrie (2017b)	Canada	Schizophrenia<br>Bipolar disorder	Immune disorders	IRR: 1.71 (95%CI: 0.73-4.01)[1]<br>IRR: 1.88 (95%CI: 1.35-2.62)[1]
Nielsen (2017)	Denmark	Substance abuse	Schizophrenia	HR: 6.04 (95%CI: 5.84-6.26)
Okkels (2017)	Denmark	PTSD	Non-affective disorders<br>Schizophrenia<br>Bipolar disorder	IRR: 2.34 (95%CI: 1.46-3.53)<br>IRR: 3.80 (95%CI: 2.33-5.80)<br>IRR: 4.22 (95%CI: 2.25-7.13)
Wang (2017)	Taiwan	Asthma	Schizophrenia	HR: 1.40 (95%CI: 1.05-1.87)

[1] This study investigates the relative risk of developing an immune disorder after developing schizophrenia or bipolar disorder. The IRR for bipolar disorder is within three years of an immune disorder diagnosis.

Seven citations reported rates deriving from the army, army conscripts or army veterans[39–45]. These studies are summarised in Table 6 below. A further study used data from the Israeli draft board[46], but considering the universal nature of the Israeli draft (both men and women are drafted into the army), this is included in the main analysis. Three USA-based studies examined the incidence of non-affective disorders (160 per 100,000 combat-years) and bipolar disorder (120) during combat[39], and of schizophrenia in the military (IR: 14.3 per 100,000 person-years[40]). A further USA-based study examined incidence of schizophrenia and bipolar disorder in army veterans[45] (see Table 6). Three Swedish conscript studies examined the effect of various risk factors on the incidence of psychotic disorders. One citation reported the effects of frequent cannabis use on risk of developing non-affective disorders (OR: 2.0, 95%CI: 0.8-4.7), schizophrenia (OR: 3.7, 95%CI: 2.3-5.8) and brief psychosis (OR: 2.2, 95%CI: 1.0-4.7)[41]. Two further citations examined the effects of resting heart rate on schizophrenia (HR for resting heart rate of >82 beats per minute: 1.10 (95%CI: 1.08-1.12) and bipolar disorder (OR: 1.01, 85%CI: 1.00-1.03)[42] and the effects of cardiovascular fitness on non-affective (HR for low fitness: 1.44, 95%CI:1.29-1.61) and other psychotic disorders (HR: 1.41, 95%CI: 1.27-1.56)[44]. A final citation found an incidence of non-affective disorders of 11 per 100,000 person-years and of schizophrenia of 3 among English army personnel[43].

Supplemental Table 8: Details of citations covering the military or army veterans

First author (year)	Country	Army group	Outcome	Incidence rate (95%CI)
Goodman (2011)	USA	Combat (Iraq War)	Non-affective disorders<br>Bipolar disorder	160[1]<br>120
Cowan (2011)	USA	Military	Schizophrenia	14.3
Manrique-Garcia (2012)	Sweden	Conscripts	Non-affective disorders<br>Schizophrenia<br>Brief psychosis	Not reported, examined the influence of cannabis use on psychosis.
Latvala (2016)	Sweden	Conscripts	Schizophrenia<br>Bipolar disorder	Not reported, examined the influence of resting heart rate on psychosis
Turner (2006)	England	Army personnel	Non-affective disorders<br>Schizophrenia	11<br>3
Nyberg (2018)	Sweden	Conscripts	Non-affective disorders<br>Other psychotic disorders	Not reported, examined the influence of cardiovascular fitness on psychosis
Ramsey (2017)	USA	Veterans (Iraqi Freedom, Enduring Freedom, New Dawn)	Schizophrenia<br><br><br>Bipolar disorder	Ranged from 0.05 (0.02 -0.10; women aged 45-64) to 0.47 (0.45-0.49, men aged 18-29)<br>Ranged from 1.88 (1.81-1.96, men aged 45-64) to 4.78 (4.63-4.94, women aged 18-29)

[1] per 100,000 combat years

Five citations investigated post-partum psychosis in Scandinavia[47–51]. Three citations contained data from Sweden, and found an incidence of 4.84 per 1,000 person-years[47]. Older maternal age was a risk factor[47,48], as

well as being a single mother[48]. Having comorbid diabetes or having a baby with high birthweight lowered risk of developing post-partum psychosis[47], and there was no difference in risk between first-time mothers who conceived naturally and those who used IVF[51]. Two further citations included data from Denmark[49,50]. Both citations looked at any psychiatric contact, although one citation specifically reported the relative risk for schizophrenia (5.65, 95%CI: 3.47-9.20) and bipolar disorder (23.33, 95%CI: 11.52-47.24) in the first thirty days after birth, compared with women in the general population[49].

A final study examined psychotic disorders in incarcerated 15-21 year-olds, and found that during the study period fewer people went to prison in total, increasing the proportion of individuals with a psychotic disorder in prison[52].

Incidence rates pooled by countries' World Bank economic classification.

Supplemental Figures 1-4 below group the incidence rates by countries' World Bank Economic Classification (June 2018 edition)[53]. There is little difference in incidence rates between high-income countries and low- and middle-income countries (LMICs), although any formal comparisons are hampered by a lack of studies conducted in LMICs.

Supplemental Figure 1: Incidence of all psychotic disorders by countries' economic classification

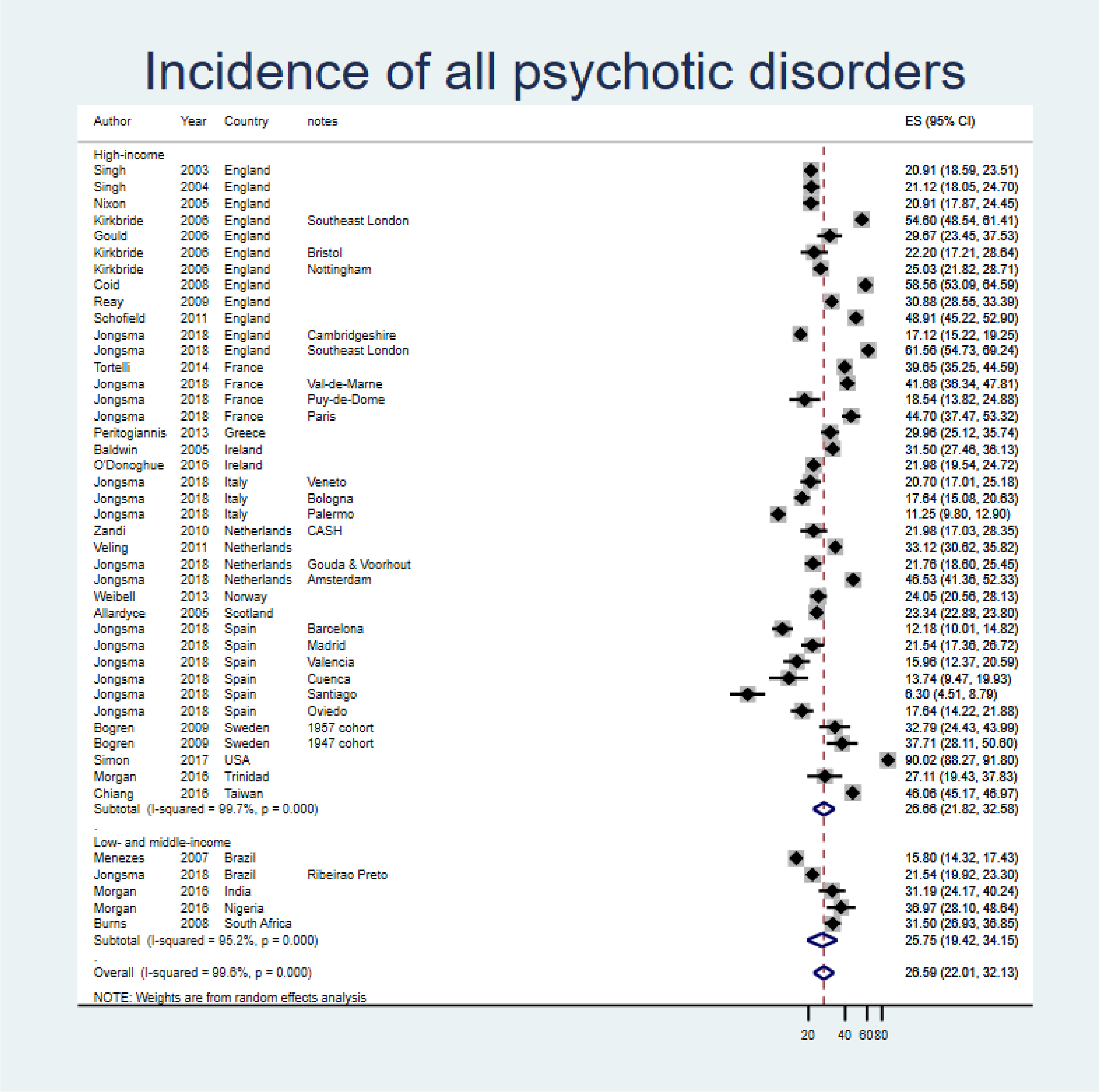

The column ‘notes’ is used to differentiate estimates resulting from the same citation (but different catchment areas or time periods).

Supplemental Figure 2: Incidence of non-affective psychotic disorders by countries' economic classification.

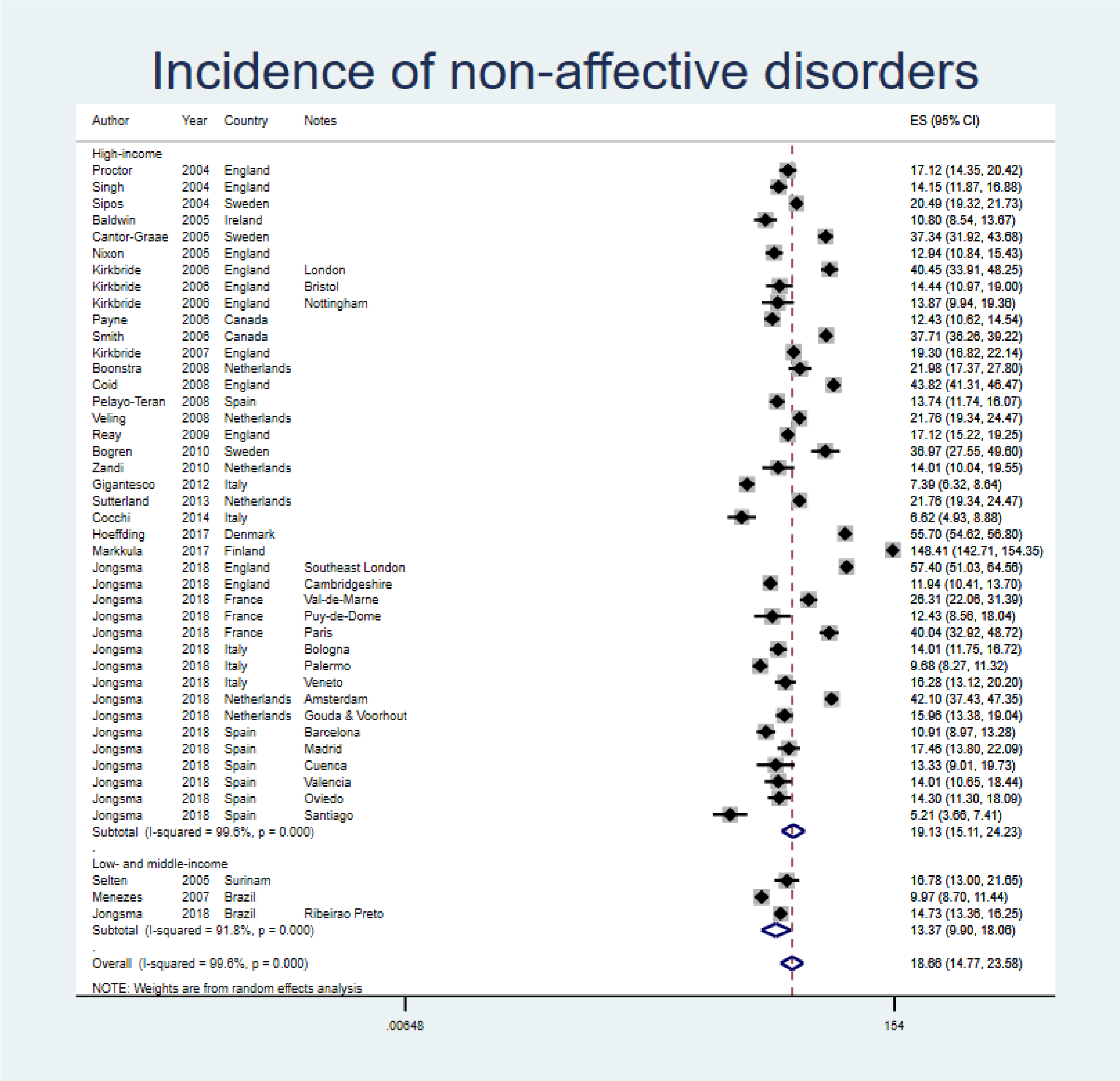

The column ‘Notes’ is used to differentiate estimates resulting from the same citation (but different catchment areas or time periods).

Supplemental Figure 3: Incidence of schizophrenia by countries' economic classification.

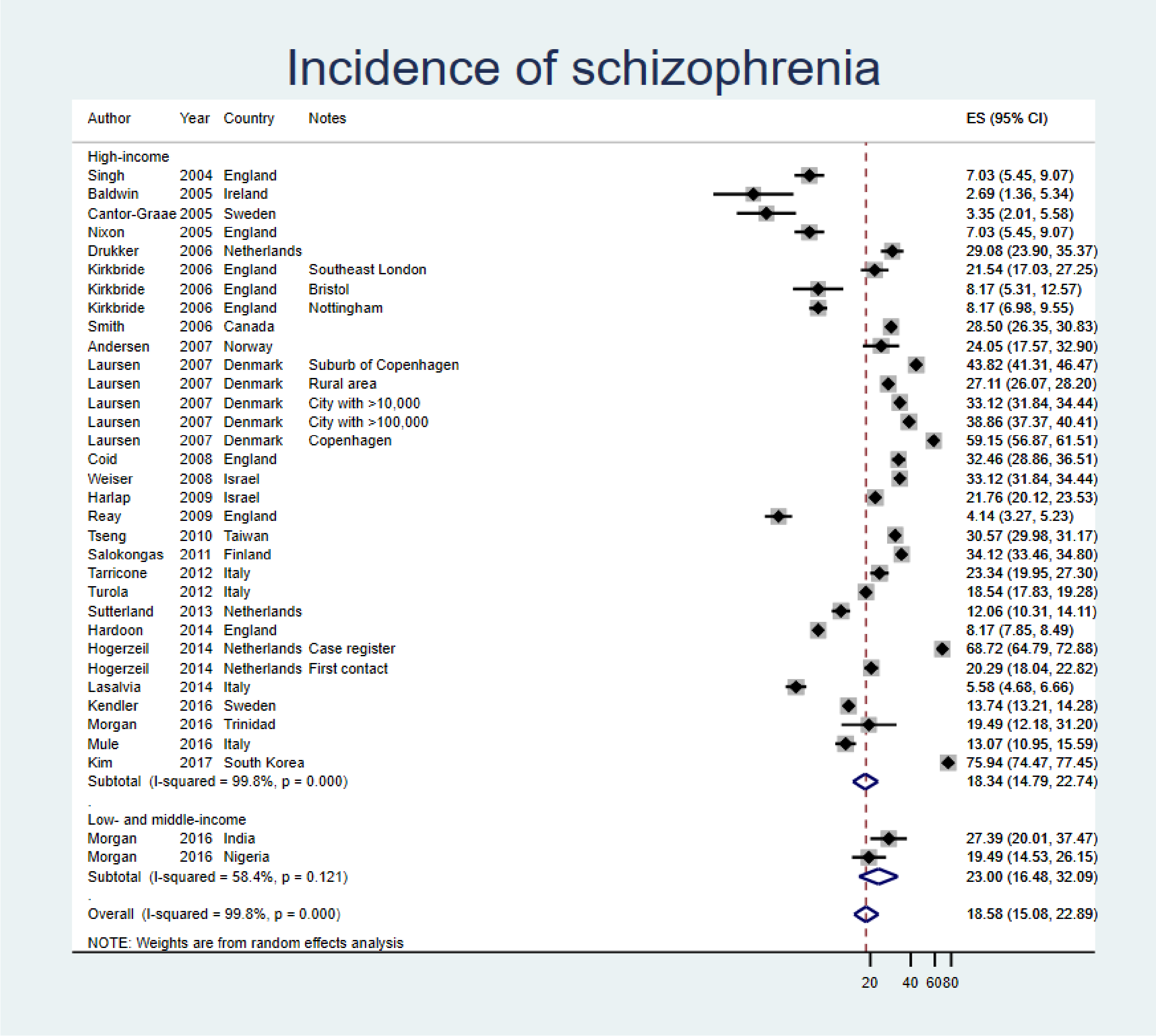

The column ‘Notes’ is used to differentiate estimates resulting from the same citation (but different catchment areas or time periods).

Supplemental Figure 4: Incidence of affective disorders by countries' economic classification

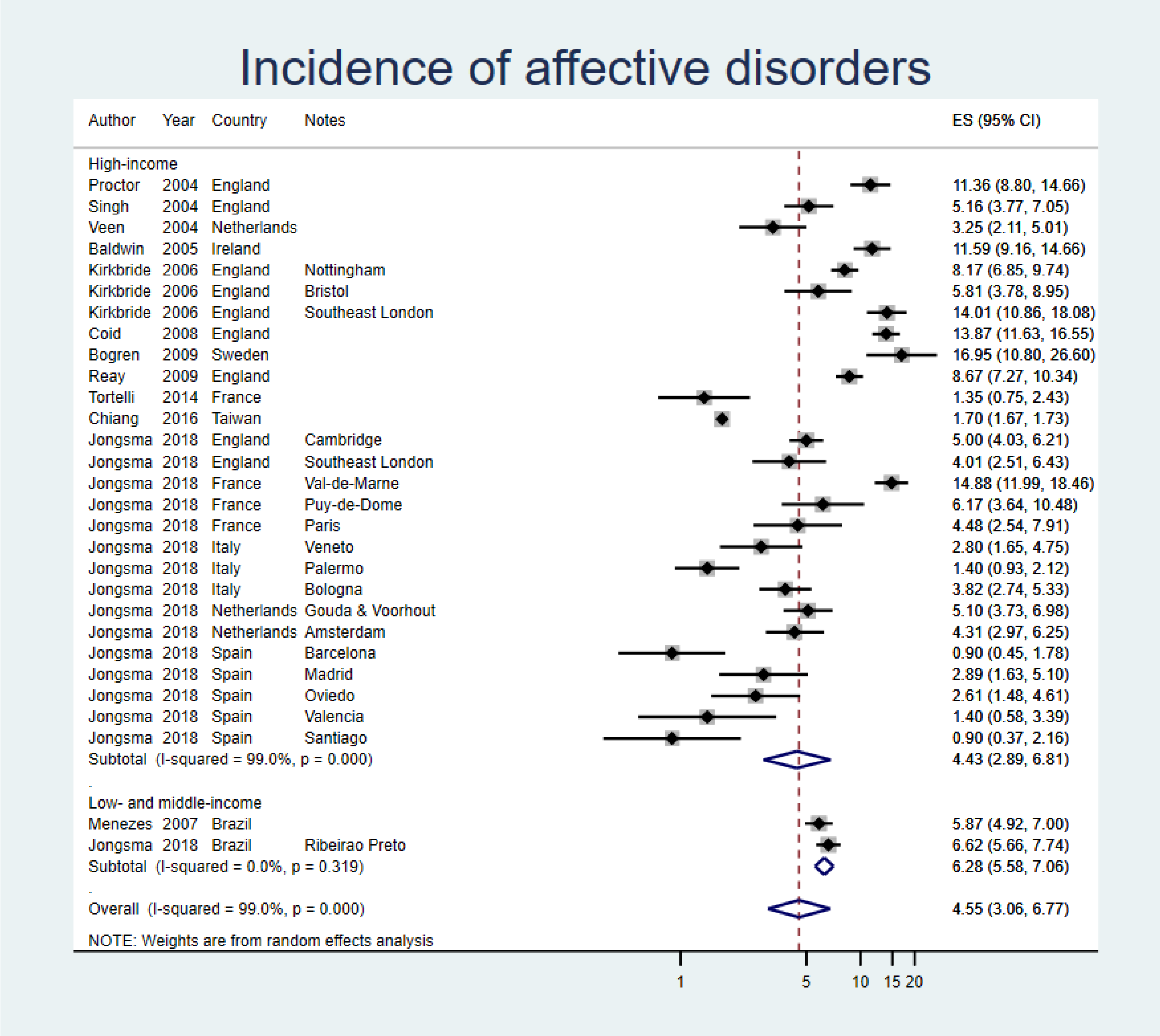

The column 'Notes' is used to differentiate estimates resulting from the same citation (but different catchment areas or time periods).

Incidence rate ratios by sex.

Below is the figure displaying all incidence rate ratios by sex (men : women) for all FEP, non-affective disorders, schizophrenia, affective disorders and bipolar disorder. Insufficient citations were available to pool IRRs for psychotic depression.

Supplemental Figure 5: Incidence rate ratios (men : women) by diagnostic outcome

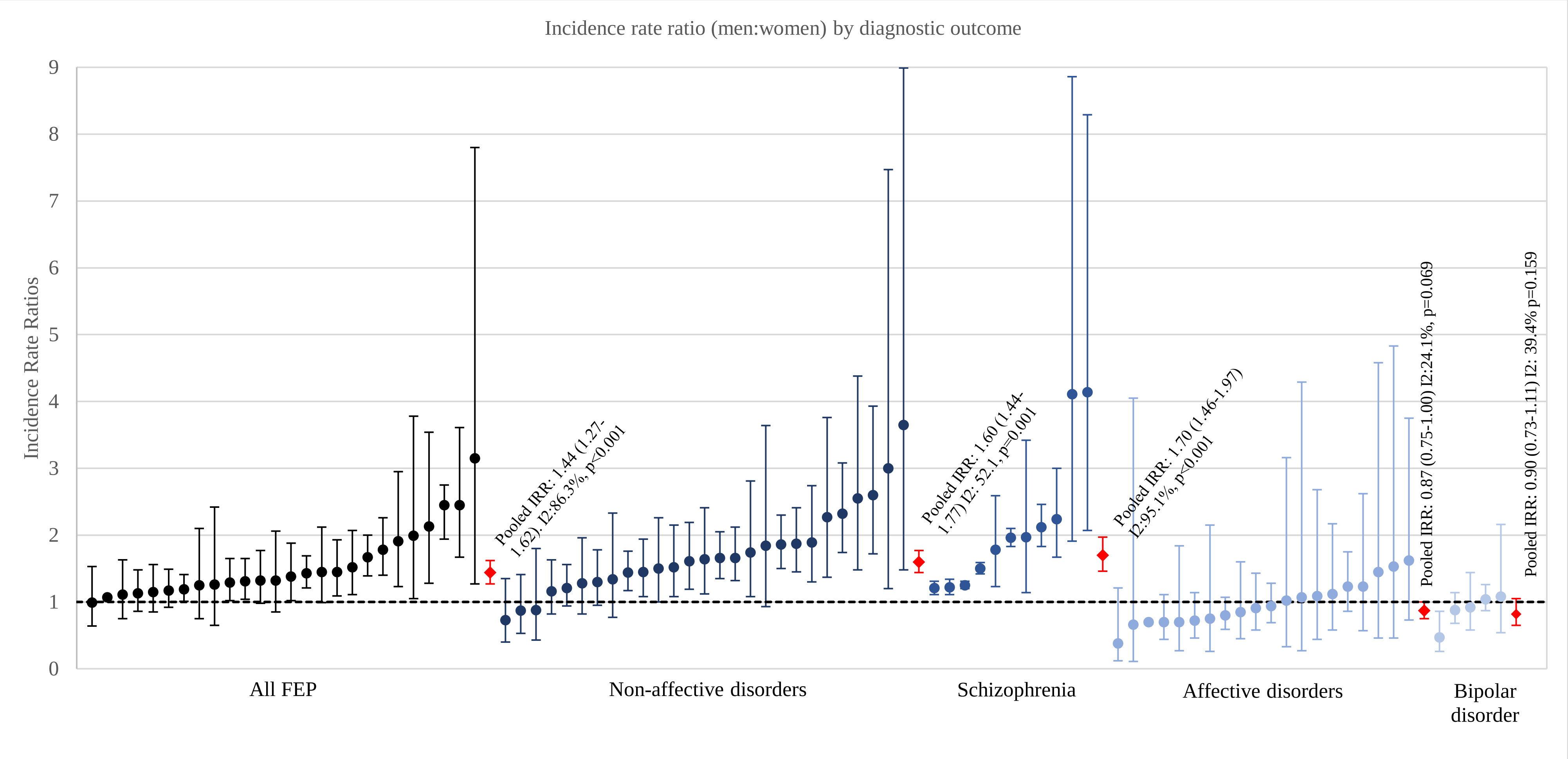

Incidence rate ratios by minority status.

Below is the figure displaying all incidence rate ratios (minority : majority) for all FEP, non-affective disorders and schizophrenia. Insufficient citations were available to synthesise results for other diagnostic outcomes.

Supplemental Figure 6: Incidence rate ratio (minority : majority) by diagnostic outcome

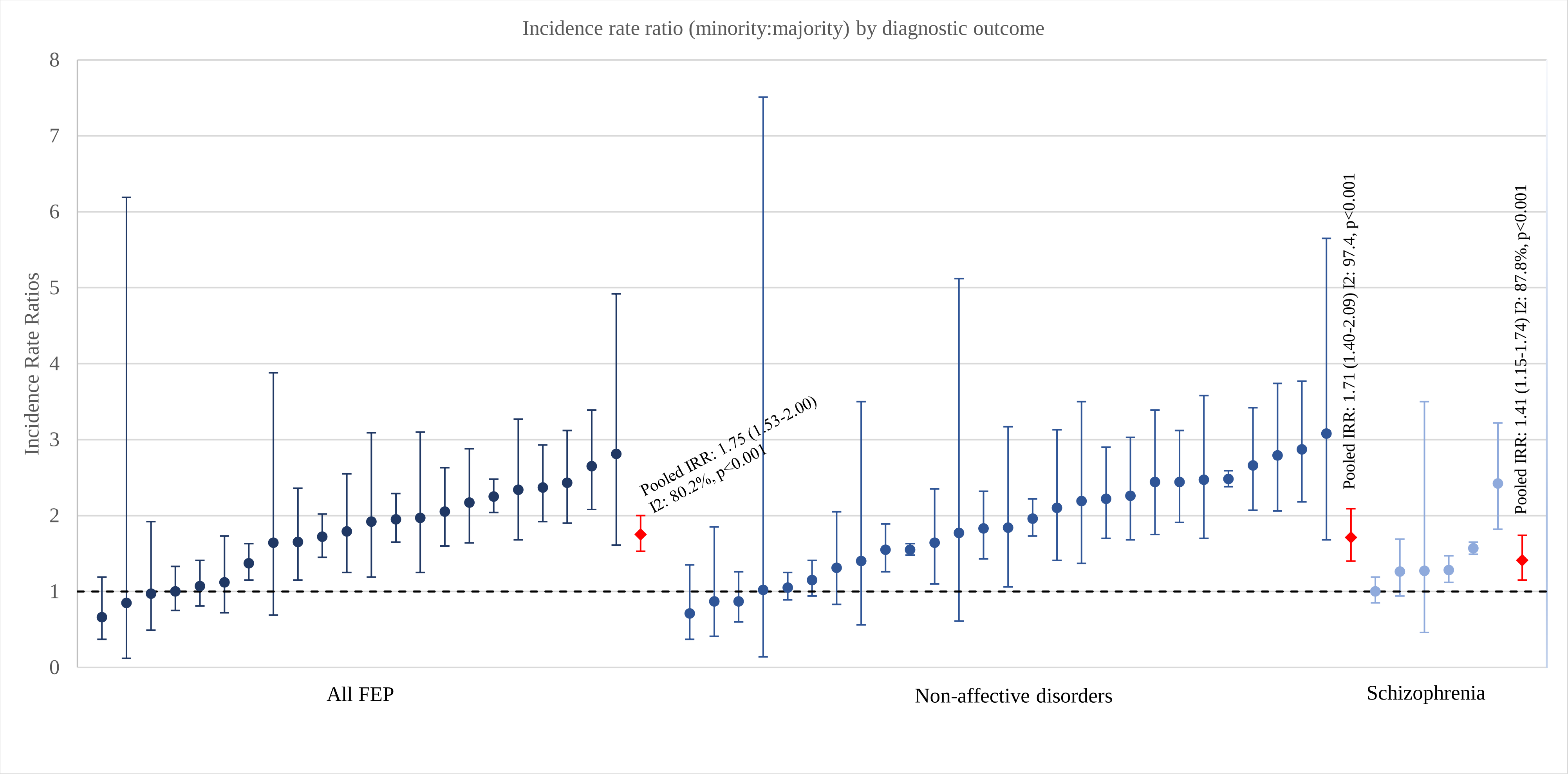

Incidence rate ratios by ethnic group

Grouping all ethnic minorities together to a general minority group masks heterogeneity between ethnic groups. The below forest plots give a detailed overview of which ethnic minority groups in which country are at highest risk of which psychotic disorder.

For all psychotic disorders, the highest risk is exhibited by Sub-Saharan African migrants in Paris (IRR:7.26, 95%CI: 5.45)[54], and the Indian population in East-Anglia is the only minority group with a lower risk than the white (British) population (IRR: 0.23, 95%CI: 0.06-0.91; Supplemental Figure 7)[6].

Supplemental Figure 7: Incidence rate ratios by ethnic group, all psychotic disorders.

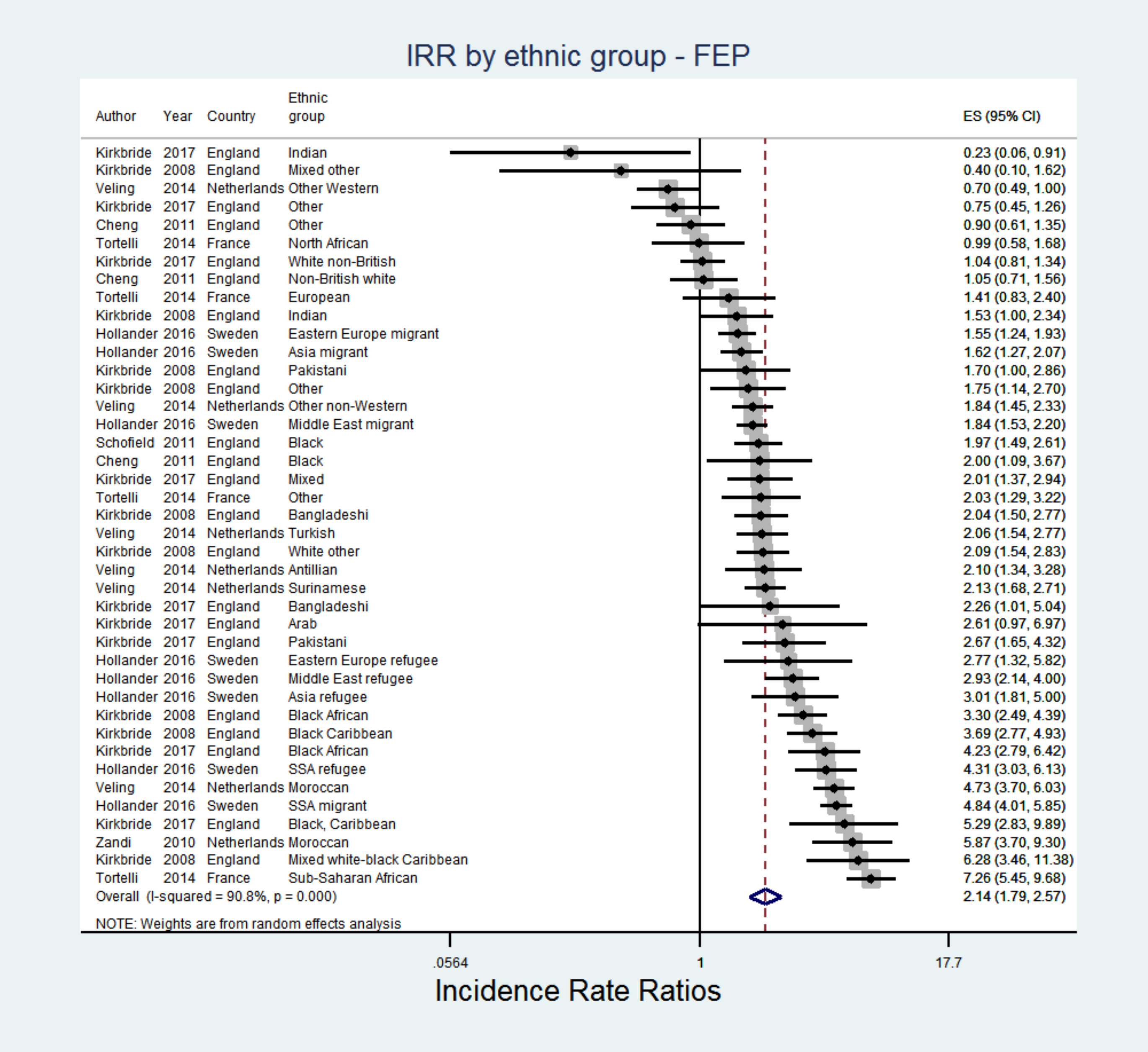

The highest risk for non-affective disorders is exhibited by Moroccan migrants to the Hague in the Netherlands (IRR: 6.04, 95%CI: 4.06-8.99)[55], and the lowest risk by the Indian minority population in East Anglia (IRR: 0.28, 95%CI:0.07-1.12; Supplemental Figure 8)[7].

Supplemental Figure 8: Incidence rate ratios by ethnic group, non-affective disorders

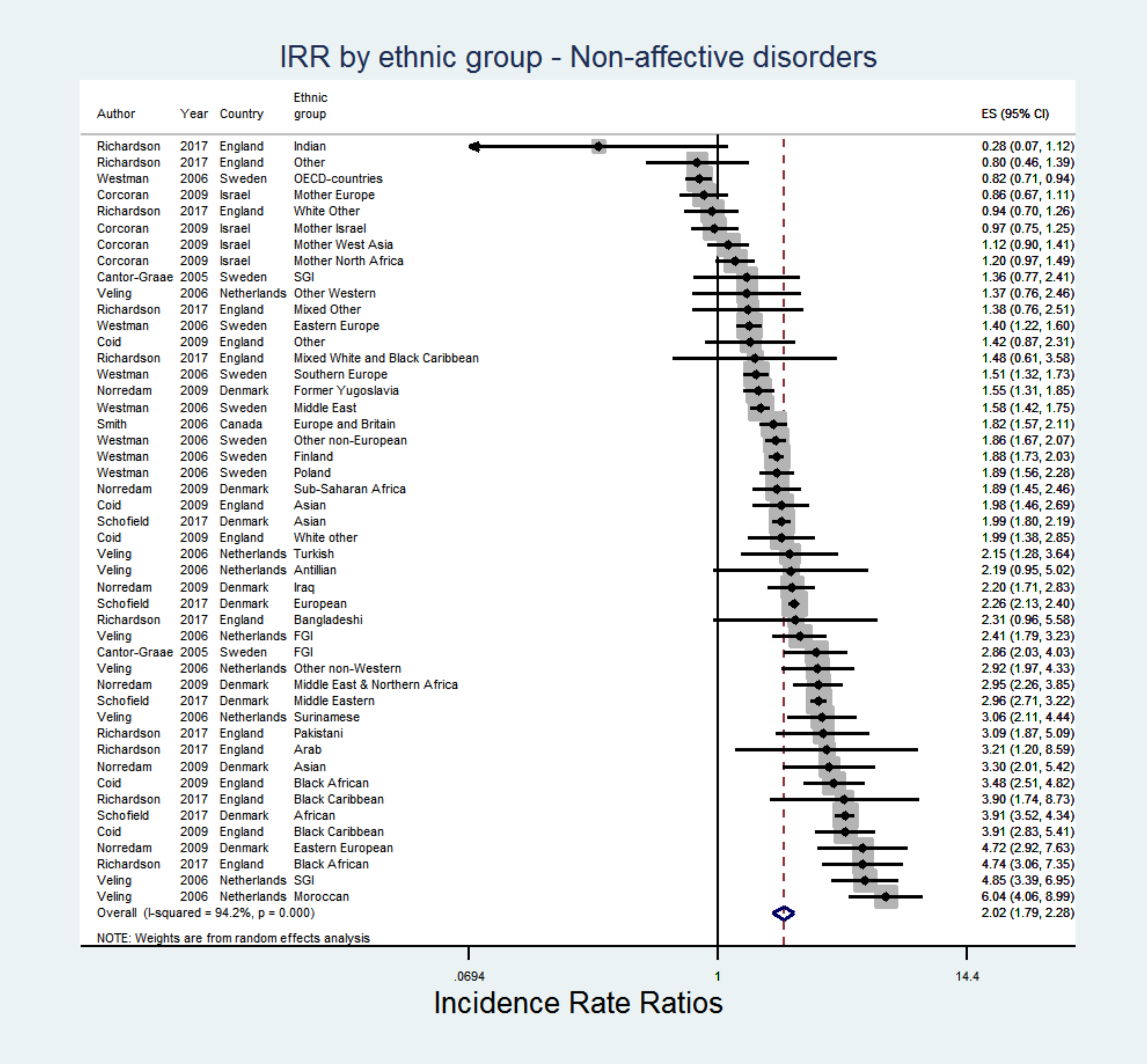

The lowest incidence rate ratio for schizophrenia is recorded in migrants to the Netherlands falling under the ‘other’ category (IRR: 0.25,95%CI: 0.21-0.30)[56] and the highest IRR recorded for a subgroup of migrants is of second-generation migrants to Denmark, where one or both parents originate from Greenland (IRR: 4.25, 95%CI: 3.37-5.37; Supplemental Figure 9 below)[57].

Supplemental Figure 9: Incidence rate ratios by ethnic group, schizophrenia

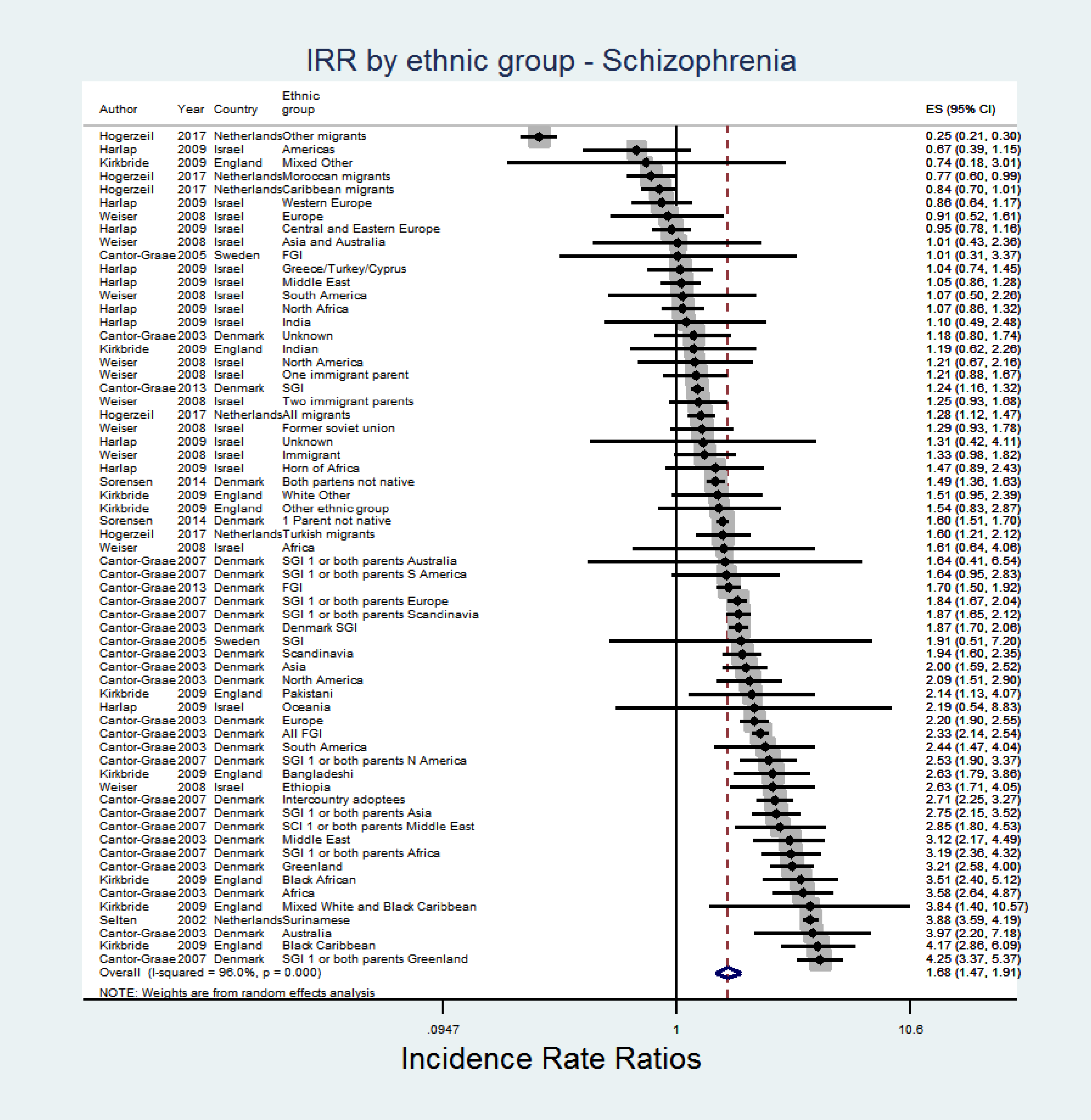

Incidence by age groups

Supplemental Figures 10 to 12 below display incidence rates by age group for all psychotic disorders, non-affective disorders and schizophrenia. Rates are grouped by citation, and ordered from youngest to oldest age group. Overall, incidence appears to be higher in younger age groups.

Supplemental Figure 10: Incidence by age group, all psychotic disorders

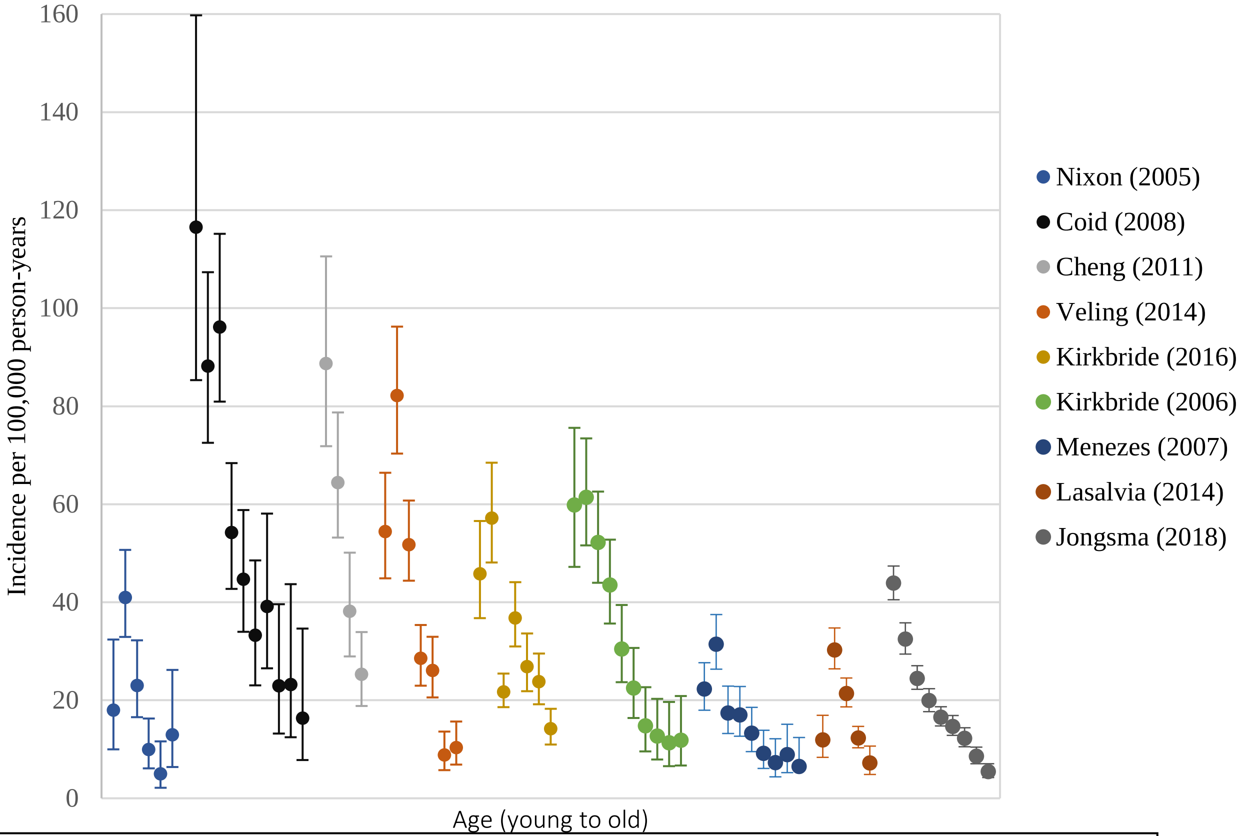

Age groups used are as follows:
Nixon (2005): 16-19, 20-29-, 30-39, 40-49, 50-59, 60-64.
Coid (2008) and Kirkbride (2006): 16-19, 20-24, 25-29, 30-34, 35-39, 40-44, 45-49, 50-54, 55-59, 60-64
Cheng (2011): 17-19, 20-24, 25-29, 30-35
Veling (2014): 15-19, 20-24, 25-29, 30-34, 35-39, 44-44, 45-49
Kirkbride (2016): 16-17, 18-19, 20-22, 23-35, 26-28, 29-31, 32-35
Menezes (2007) and Jongsma (2018): 18-24, 25-29, 30-34, 35-39, 40-44, 45-49, 50-54, 55-59, 60-64
Lasalvia (2014): 15-19, 20-9, 30-39, 40-49, 50-54

Supplemental Figure 11: Incidence by age group, non-affective disorders

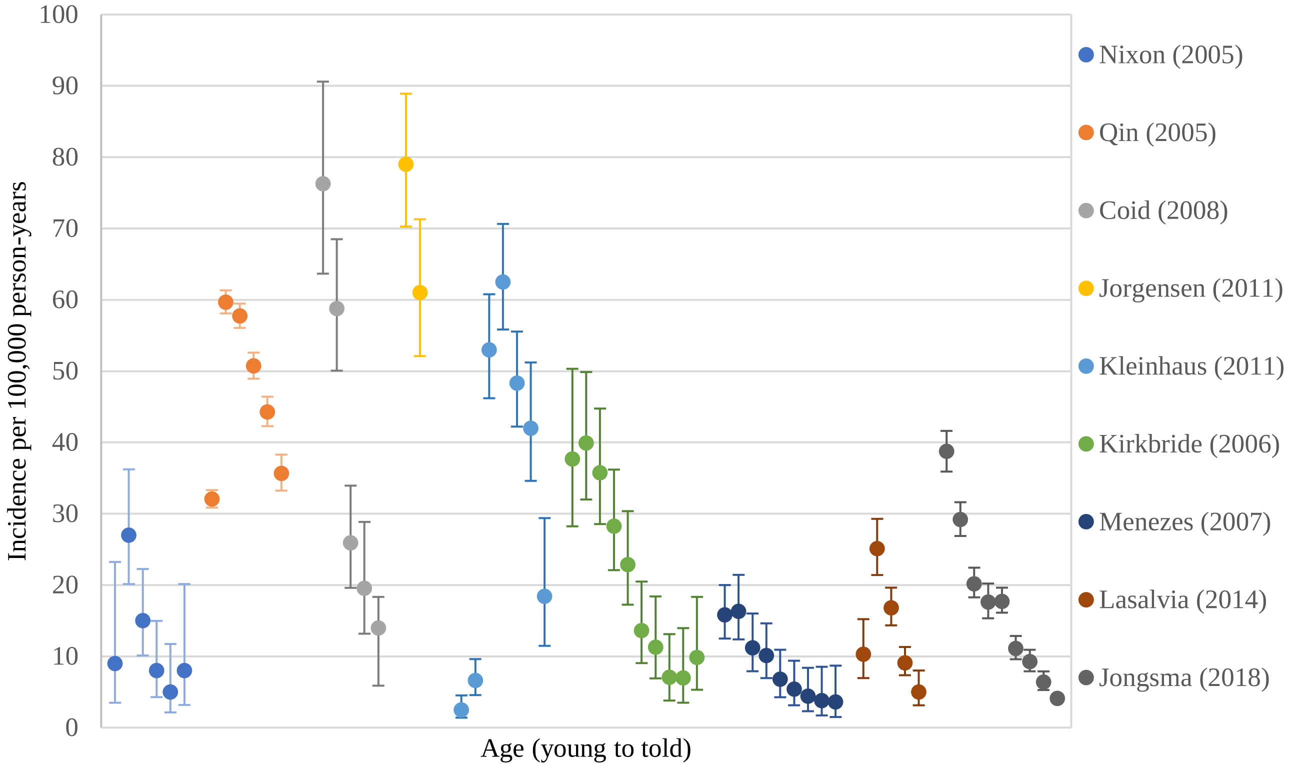

Age groups used are as follows:
Nixon (2005): 16-19, 20-29-, 30-39, 40-49, 50-59, 60-64.
Qin (2005): 15-19, 20-24, 25-29, 30-34, 35-39, 40-44, 45+
Coid (2008): 18-24, 25-34, 35-44, 45-54, 55-64
Jorgensen(2011): 18-34, 35-44
Kleinhaus(2011): 5-9, 10-14, 15-19, 20-24, 25-29, 30-34, 35-39
Kirkbride (2006): 16-19, 20-24, 25-29, 30-34, 35-39, 40-44, 45-49, 50-54, 55-59, 60-64
Menezes (2007) and Jongsma (2018): 18-24, 25-29, 30-34, 35-39, 40-44, 45-49, 50-54, 55-59, 60-64
Lasalvia (2014): 15-19, 20-9, 30-39, 40-49, 50-54

Supplemental Figure 12: Incidence by age group, schizophrenia

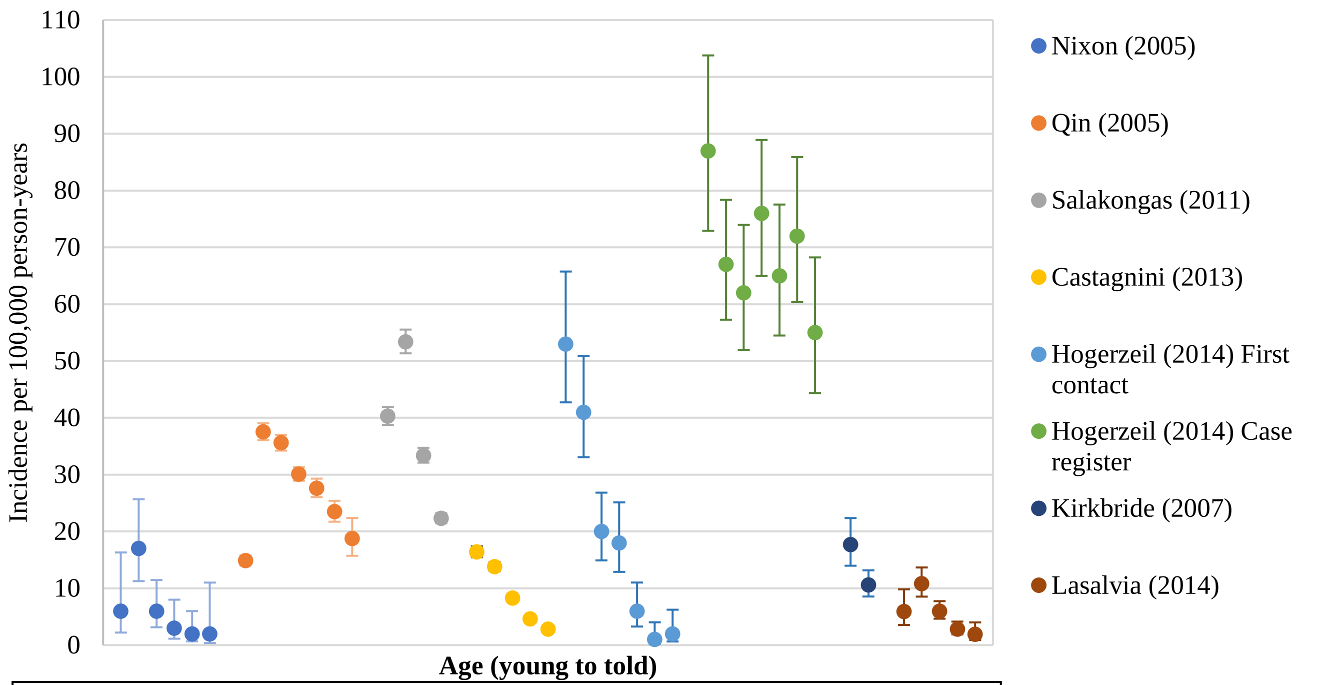

Age groups used are as follows:
Nixon (2005): 16-19, 20-29-, 30-39, 40-49, 50-59, 60-64.
Qin (2005): 15-19, 20-24, 25-29, 30-34, 35-39, 40-44, 45+
Salakongas (2011): 15-24, 25-34, 35-44, 45-64
Castagnini (2013): 15-24, 25-34, 35-44, 45-54, 55-64
Hogerzeil (2014): 20-24, 25-29, 30-34, 35-39, 40-44, 45-49, 50-54
Kirkbride (2007): 16-29, 30-64
Lasalvia (2014): 15-19, 20-29, 30-29, 40-49, 50-54

Meta-regression

Supplemental Tables 9-13 below include both crude and adjusted results of meta-regression for each outcome with study quality (1-7) time period (middle-year of recruitment period) and study design (first contact, a combination, cohort, case register or population register) as exposure variables.

Results by study design are summarised in the main text. No incidence rate (ratio) of any outcome was associated with study quality in a multivariable model. Later citations reported slightly lower incidence rate ratios for minority groups compared with the majority for non-affective disorders (IRR: 0.97, 95%CI: 0.95-1.00) and a slightly lower incidence of affective disorders (IRR: 0.96, 95%CI: 0.92-0.99).

Supplemental Table 9: Meta-regression all FEP

	Psychotic disorders, overall incidence				Incidence rate ratio by sex				Incidence rate ratio by minority status			
**Exposure**	*Crude association*		*Adjusted association[3]*		*Crude association*		*Adjusted association[3]*		*Crude association*		*Adjusted association[4]*	
	β (95%CI)	p-value	β (95%CI)	p- value	β (95%CI)	p-value	β (95%CI)	p-value	β (95%CI)	p-value	β (95%CI)	p-value
Study quality[1]	0.94 (0.78-1.14)	0.53	0.96 (0.80-1.11)	0.52	1.06 (0.95-1.20)	0.29	1.01 (0.85-1.20)	0.95	1.04 (0.52-2.07)	0.83	--	--
Time period[2]	0.99 (0.98-1.01)	0.42	0.99 (0.97-1.01)	0.18	1.00 (0.99-1.01)	0.92	1.00 (0.98-1.01)	0.59	0.99 (0.95-1.02)	0.45	--	--
Study design												
First contact	Reference		Reference		Reference		Reference		Reference		Reference	
Combination	0.78 (0.21-2.87)	0.71	0.40 (0.09-1.73)	0.21	0.94 (0.55-1.62)	0.83	0.79 (0.31-2.03)	0.61	--	--	--	--
Cohort	--	--	--	--	--	--	--	--	--	--	--	--
Case register	--	--	--	--	--	--	--	--	--	--	--	--
Population register	--	--	--	--	0.73 (0.47-1.12)	0.14	0.71 (0.36-1.38)	0.291	1.31 (0.68-2.51)	0.40	--	--

[1] Ranged from 1 – 7 [2]Measured as middle year of recruitment [3]Adjusted for other variables in model (study quality, time period, study design) [4]Insufficient observations available

Effect sizes in **bold** are statistically significant (p<0.05)

Supplemental Table 10: Meta-regression non-affective disorders

	Non-affective disorders, overall incidence				Incidence rate ratio by sex				Incidence rate ratio by minority status			
**Exposure**	*Crude association*		*Adjusted association[3]*		*Crude association*		*Adjusted association[3]*		*Crude association*		*Adjusted association[3]*	
	β (95%CI)	p-value	β (95%CI)	p- value	β (95%CI)	p-value	β (95%CI)	p-value	β (95%CI)	p-value	β (95%CI)	p-value
Study quality[1]	1.04 (0.86-1.25)	0.66	1.11 (0.95-1.30)	0.18	1.02 (0.90-1.15)	0.786	1.02 (0.89-1.16)	0.80	1.03 (0.79-1.34)	0.83	0.82 (0.83-1.06)	0.12
Time period[2]	1.00 (1.00-1.00)	0.94	1.00 (1.00-1.00)	0.568	1.00 (0.99-1.01)	0.926	1.00 (0.99-1.02)	0.77	0.99 (0.98-1.01)	0.55	**0.97 (0.95-1.00)**	**0.03**
Study design												
First contact	Reference		Reference		Reference		Reference		Reference		Reference	
Combination	0.74 (0.21-2.59)	0.63	1.07 (0.19-2.78)	0.76	--	--	--	--	--	--	--	--
Cohort	--	--	--	--	--	--	--	--	0.79 (0.35-1.81)	0.57	**0.43 (0.20-0.92)**	**0.03**
Case register	0.98 (0.28-3.42)	1.00	1.08 (0.6.01)	0.616	--	--	--	--	--	--	--	--
Population register	**5.31 (2.21-13.27)**	**0.001**	**9.64 (2.92-31.82)**	**<0.001**	--	--	--	--	0.72 (0.37-1.43)	0.34	**0.42 (0.22-0.83)**	**0.01**

[1] Ranged from 1 – 7 [2]Measured as middle year of recruitment [3]Adjusted for other variables in model (study quality, time period, study design)

Effect sizes in **bold** are statistically significant (p<0.05)

Supplemental Table 11: Meta-regression schizophrenia

	Schizophrenia, overall incidence				Incidence rate ratio by sex				Incidence rate ratio by minority status			
**Exposure**	*Crude association*		*Adjusted association[3]*		*Crude association*		*Adjusted association[3]*		*Crude association*		*Adjusted association[4]*	
	β (95%CI)	p-value	β (95%CI)	p- value	β (95%CI)	p-value	β (95%CI)	p-value	β (95%CI)	p-value	β (95%CI)	p-value
Study quality[1]	0.82 (0.64-1.05)	0.11	1.00 (0.77-1.30)	0.99	1.16 (0.93-1.46)	0.17	1.10 (0.66-1.83)	0.59	1.36 (0.94-1.97)	0.08	--	--
Time period[2]	0.99 (0.98-1.01)	0.40	0.99 (0.8-1.01)	0.29	1.01 (0.99-1.03)	0.57	1.02 (0.94-1.10)	0.52	1.00 (0.99-1.03)	0.40	--	--
Study design												
First contact	Reference		Reference		Reference		Reference		Reference		Reference	
Combination	0.63 (0.13-2.24)	0.31	0.45 (0.09-2.31)	0.33	1.10 (0.29-4.17)	0.87	2.06 (0.05-84.01)	0.58	0.57 (0.21-1.60)	0.15	--	--
Cohort	**3.10 (1.12-8.53)**	**0.03**	**3.27 (1.06-10.12)**	**0.04**	0.84 (0.40-1.76)	0.59	0.71 (0.13-3.76)	0.56	0.45 (0.15-1.29)	0.08	--	--
Case register	**3.12 (1.33-7.29)**	**0.01**	**3.95 (1.22-12.83)**	**0.02**	0.68 (0.38-1.25)	0.18	0.79 (0.11-5.57)	0.72	--	--	--	--
Population register	**2.50 (1.36-4.55)**	**0.004**	**2.54 (1.24-5.21)**	**0.01**	1.13 (0.61-2.10)	0.64	1.18 (0.29-4.91)	0.73	0.65 (0.25-1.67)	0.19	--	--

[1] Ranged from 1 – 7 [2]Measured as middle year of recruitment [3]Adjusted for other variables in model (study quality, time period, study design) [4]Insufficient observations available
Effect sizes in **bold** are statistically significant (p<0.05)

Supplemental Table 12: Meta-regression affective psychotic disorders

	Affective psychotic disorders, overall incidence				Incidence rate ratio by sex			
**Exposure**	*Crude association*		*Adjusted association[3]*		*Crude association*		*Adjusted association[3]*	
	β (95%CI)	p-value	β (95%CI)	p- value	β (95%CI)	p-value	β (95%CI)	p-value
Study quality[1]	1.04 (0.77-1.39)	0.80	1.31 (0.94-1.84)	0.11	**1.15 (1.07-1.24)**	**0.001**	1.12 (0.92-1.35)	0.26
Time period[2]	0.96 (0.91-1.00)	0.06	**0.96 (0.92-0.99)**	**0.02**	1.00 (0.99-1.02)	0.66	1.00 (0.98-1.02)	0.80
Study design								
First contact	Reference		Reference		Reference		Reference	
Combination	--	--	--	--	--	--	--	--
Cohort	--	--	--	--	--	--	--	--
Case register	2.58 (0.28-23.95)	0.39	6.64 (0.71-62.09)	0.09	--	--	--	--
Population register	--	--	--	--	**0.76 (0.65-0.88)**	**0.001**	0.91 (0.57-1.46)	0.67

[1] Ranged from 1 – 7 [2]Measured as middle year of recruitment [3]Adjusted for other variables in model (study quality, time period, study design)
Effect sizes in **bold** are statistically significant (p<0.05)

Supplemental Table 13: Meta-regression bipolar disorder with psychosis

	Bipolar disorder with psychosis, overall incidence				Incidence rate ratio by sex			
**Exposure**	*Crude association*		*Adjusted association*[3]		*Crude association*		*Adjusted association*[4]	
	β (95%CI)	p-value	β (95%CI)	p- value	β (95%CI)	p-value	β (95%CI)	p-value
Study quality[1]	0.82 (0.59-1.15)	0.24	0.95 (0.71-1.26)	0.69	0.96 (0.72-1.57)	0.77	--	--
Time period[2]	1.00 (0.98-1.02)	0.89	1.00 (0.1.00-1.02)	0.13	0.99 (0.93-1.04)	0.76	--	--
Study design								
First contact	Reference		Reference		Reference		Reference	
Combination	2.08 (0.52-8.35)	0.28	2.45 (0.63-9.51)	0.18	1.13 (0.19-9.46)	0.79	--	--
Cohort	--	--	--	--	--	--	--	--
Case register	2.64 (0.66-10.59)	0.16	2.40 (0.61-9.50)	0.20	1.34 (0.24-7.35)	0.54	--	--
Population register	**4.08 (2.19-7.59)**	**<0.0001**	**4.53 (2.41-8.51)**	**<0.0001**	--	--	--	--

[1] Ranged from 1 – 7

[2] Measured as middle year of recruitment

[3] Adjusted for other variables in model (study quality, time period, study design)

[4] Insufficient observations available

Effect sizes in **bold** are statistically significant (p<0.05)

Small study effects

The below table (13) details results of the Egger's test for small study effects, where the number of studies included in the analysis is at least ten. Funnel plots of all analyses are displayed below (Figures 9-13). Orange lines in the Figures corresponds to Egger's test for funnel plot asymmetry. A negative coefficient indicates that smaller studies produce smaller outcomes, and a positive coefficient indicates that smaller studies produce larger outcomes.

Supplemental Table 14: Egger's test for small study effects

**Outcome**	***N***	**Bias (β)**	**Standard error**	**p-value**
All FEP				
Overview	44	**-7.53**	**3.14**	**0.02**
By sex	26	**2.16**	**0.44**	**<0.0001**
By minority status	22	-1.53	0.89	0.10
Non-affective disorders				
Overview	43	**-14.55**	**2.46**	**<0.0001**
By sex	27	0.05	0.76	0.95
By minority status	28	-0.79	1.57	0.62
Schizophrenia				
Overview	34	**-11.78**	**5.52**	**0.04**
By sex	11	2.97	1.74	0.12
By minority status	6	--	--	--
Affective disorders				
Overview	29	**7.72**	**1.60**	**<0.0001**
By sex	20	**0.90**	**0.24**	**0.001**
Bipolar disorder				
Overview	24	**-14.67**	**2.69**	**<0.0001**
By sex	5	--	--	--

Estimates in **bold** are statistically significant (p<0.05) and provide evidence of small study effects.

Supplemental Figure 13: Funnel plots for all FEP

a. Overall incidence

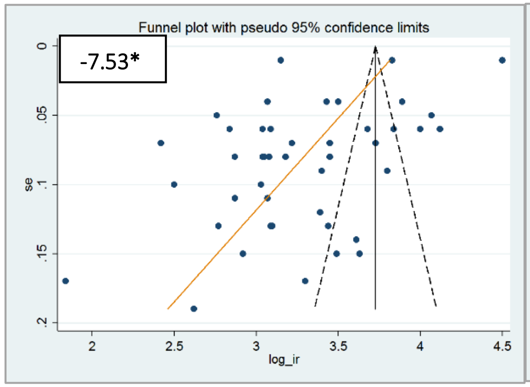

b. IRR by sex

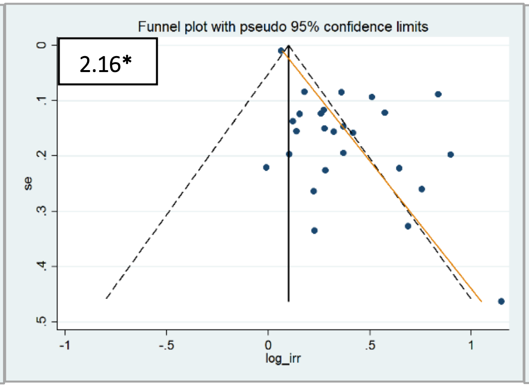

c. IRR by minority status

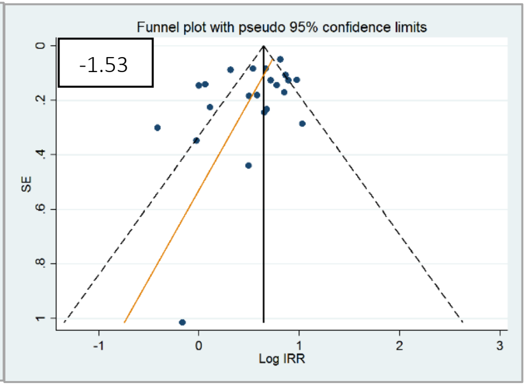

The orange line represents Egger's coefficient, and corresponds to the bias (β) in Supplemental Table 10. Coefficients in ***bold**** *are statistically significant (p<0.05)*

Supplemental Figure 14: Funnel plots of non-affective disorders

a. Overall incidence

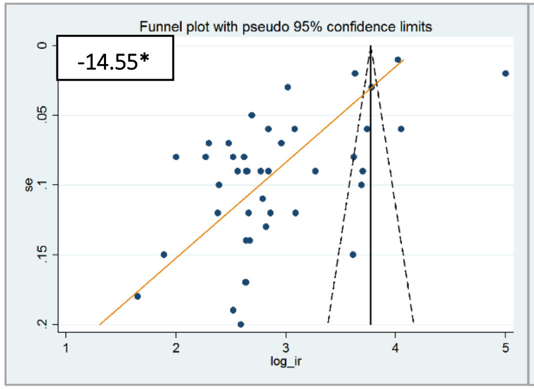

b. IRR by sex

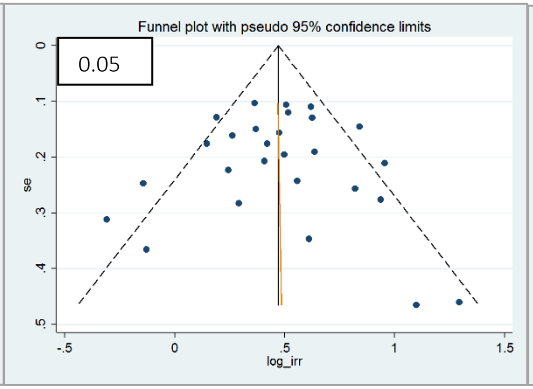

c. IRR by minority status

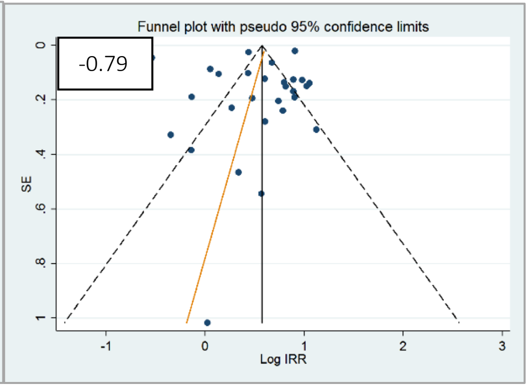

The orange line represents Egger's coefficient, and corresponds to the bias (β) in Supplemental Table 10. Coefficients in bold are statistically significant (p<0.05)*

Supplemental Figure 15: Funnel plots of schizophrenia

a. Overall incidence

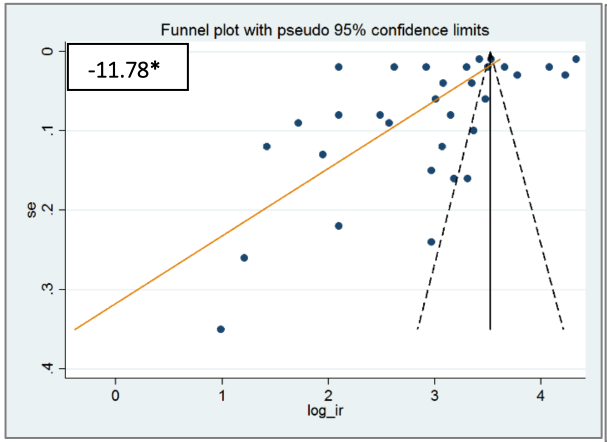

b. IRR by sex

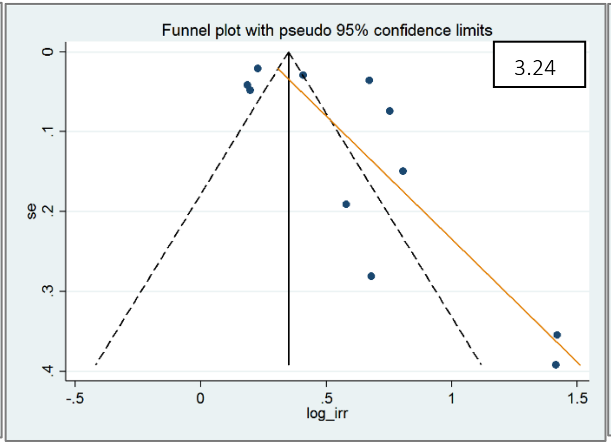

c. IRR by minority status

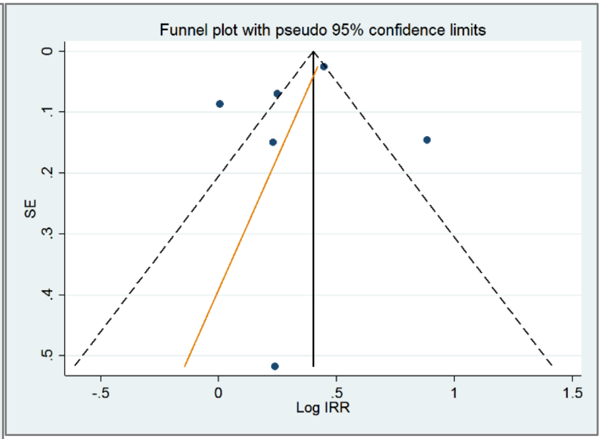

*The orange line represents Egger's coefficient, and corresponds to the bias (β) in Supplemental Table 10. Coefficients in **bold*** are statistically significant (p<0.05). No Egger's test was computed for IRR by minority status (insufficient observations).*

Supplemental Figure 16: Funnel plots of affective psychotic disorders

a. Overall incidence

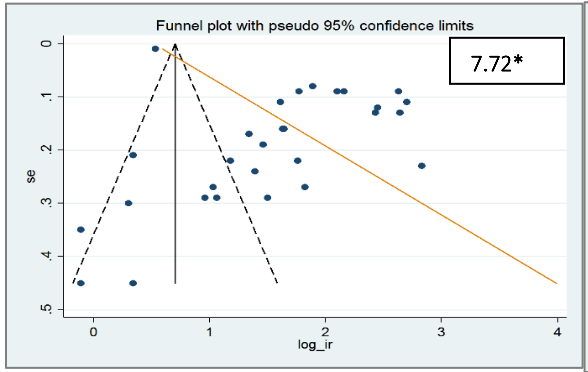

b. IRR by sex

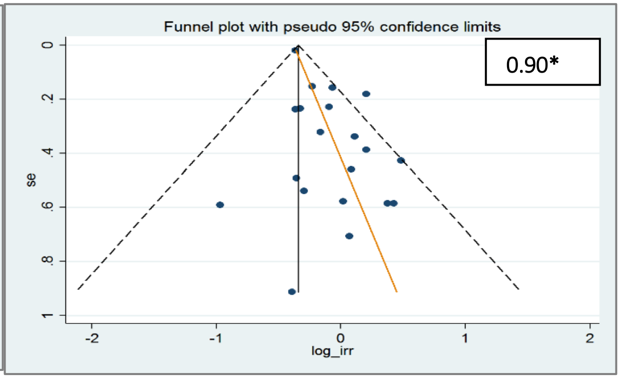

*The orange line represents Egger's coefficient, and corresponds to the bias (β) in Supplemental Table 10. Coefficients in **bold*** are statistically significant (p<0.05)*

Supplemental Figure 17: Funnel plots of bipolar disorder

a. Overall incidence **b. IRR by sex**

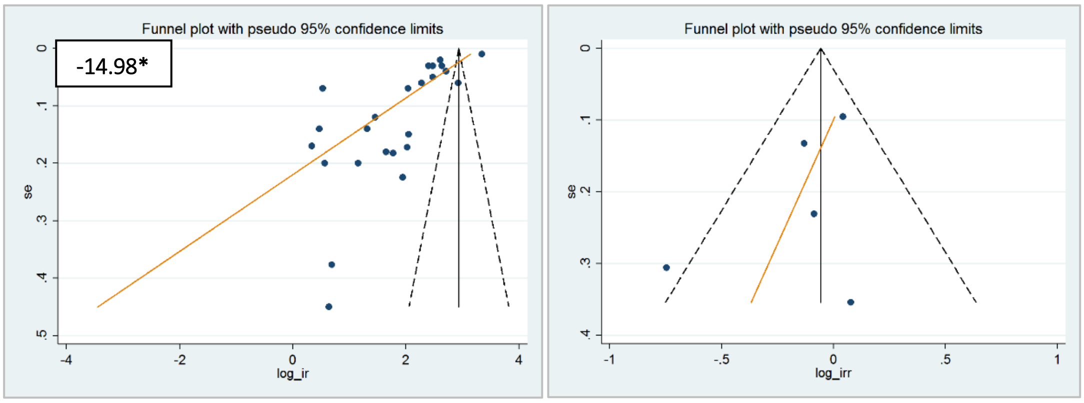

The *orange* *line represents Egger's coefficient. Coefficients in* ***bold*** *are statistically significant (p<0.05). No Egger's test was computed for IRR by sex (insufficient observations).*

In *post-hoc* sensitivity analyses, we repeated Egger's test by study type, to test our initial hypothesis that this could explain strong evidence of small study effects (Supplemental Table 15). Whilst study type appeared to explain some of the small study effects, some evidence of it remained, particularly within first contact studies.

Supplemental Table 15: Sensitivity analyses: Egger's test by study type

Outcome	Study type	*N*	Bias (β)	Standard error	p-value
All FEP	First contact				
Overview		43	**-7.43**	**3.21**	**0.03**
By sex		24	0.01	0.91	0.99
By minority status		21	-0.87	1.07	0.43
All FEP	Population register				
Overview			Insufficient observations		
By sex					
By minority status					
Non-affective disorders	First contact				
Overview		39	**-8.75**	**1.97**	**<0.0001**
By sex		27	0.05	0.76	0.95
By minority status		23	-0.53	1.09	0.63
Non-affective disorders	Population register				
Overview			Insufficient observations		
By sex					
By minority status					
Schizophrenia	First contact				
Overview		21	-7.19	4.00	0.09
By sex		5	**3.89**	**0.67**	**0.01**
By minority status		n/a			
Schizophrenia	Population register				
Overview		7	33.67	74.79	0.67
By sex		n/a			
By minority status		n/a			
Affective disorders	First contact				
Overview		28	**7.46**	**1.63**	**<0.0001**
By sex		19	0.18	0.42	0.68
Affective disorders	Population register				
Overview			Insufficient observations		
By sex					
Bipolar disorder	First contact				
Overview		15	**-8.94**	**2.46**	**0.03**
By sex		n/a			
Bipolar disorder	Population register				
Overview		7	**-25.41**	**7.11**	**0.02**
By sex		n/a			

Estimates in **bold** are statistically significant (p<0.05) and provide evidence of small study effects.

Non-affective psychotic disorders

Supplemental Table 16 below gives an overview of the exact diagnoses included in the broad 'non-affective psychotic disorders' category.

Supplemental Table 16: Exact diagnoses included in the non-affective psychotic disorders category.

First author (year)	Diagnostic manual	Outcomes included	Diagnostic codes
Hanoeman (2002)	DSM-III-R	Schizophrenia, schizophreniform disorder	None given
Proctor (2004)	ICD-10	Non-affective disorders	F20-F29
Sipos (2004)	ICD-9 & ICD-10[1]	Non-affective disorders	F20-F29
Leao (2007)	ICD-9 & ICD-10	Non-affective disorders	F20-F29
Cantor-Graae (2005)	DSM-IV	Schizophrenia, schizoaffective disorder, schizophreniform disorder	297.1, 298.9, 298.9
Qin (2005)	ICD-8 & ICD-10	Non-affective disorders	F20-F25, F28/F29
Westman (2006)	ICD-9 & ICD-10	Non-affective disorders	F20-F29
Leao (2006)	ICD-9 & ICD-10	Non-affective disorders	F20-F29
Munk-Olsen (2006)	ICD-8 & ICD-10	Non-affective disorders	F20-F29
Payne (2006)	Not stated	Schizophrenia, schizoaffective disorder, schizophreniform disorder, delusional disorder, psychosis NOS	None given
Kirkbride (2007)	ICD-10	Non-affective disorders	F20-F29
Coid (2008)	DSM-IV	Non-affective disorders	295.xx, 297.xx, 298.8, 298.9
Boonstra (2008)	DSM-IV	Schizophrenia, schizoaffective disorder, schizophreniform disorder, brief psychosis, delusional disorder, psychosis NOS	None given
Corocran (2009)	ICD-10	Non-affective disorders	F20-F29
Harlap (2009)	ICD-10	Non-affective disorders	F20-F29
Reay (2009)	ICD-10	Non-affective disorders	F20-F29
Kirkbride (2009)	ICD-10	Non-affective disorders	F20-F29
Bogren (2010)	DSM-IV	Schizophrenia, schizoaffective disorder, schizophreniform disorder, delusional disorder, brief psychosis, delusional disorder, psychosis NOS	None given
Zammit (2010)	ICD-8 & ICD-10	Non-affective disorders	F20-F29
Bogren (2009)	DSM-IV	Schizophrenia, schizoaffective disorder, schizophreniform disorder, delusional disorder, brief psychosis, delusional disorder, psychosis NOS	None given
Jorgensen (2011)	ICD-10	Non-affective disorders	F20-F29
Kleinhaus (2011)	ICD-10	Non-affective disorders	F20-F29
Benros (2011)	ICD-10	Non-affective disorders	F20-F29
Cowan (2011)	ICD-9	Schizophrenia, schizoaffective disorders, schizophreniform disorder	295.x
Manrique-Garcia (2012)	ICD-8 & ICD-10	Schizophrenia, substance-induced psychosis, delusional disorder, acute and transient psychosis, other non-organic psychosis, psychosis NOS	F20, F125/F127, F22, F23, F28, F25
Nosarti (2012)	ICD-8 & ICD-10	Schizophrenia, schizotypal disorder, acute and transient psychosis, schizoaffective disorder, other non-organic psychosis, psychosis NOS	F20, F21, F23.1/23.2, F25, F28, F29
Anderson (2012)	Not stated	Schizophrenia, schizophreniform disorder, schizoaffective disorder, delusional disorder, brief psychosis, psychosis NOS	None given
Sutterland (2013)	ICPC	Schizophrenia, schizophreniform disorder, schizoaffective disorder, brief psychosis, psychosis NOS	None given
Cantor-Graae (2007)	ICD-8 & ICD-10	Schizophrenia, schizotypal disorder, delusional disorder, acute and transient psychosis, psychosis NOS.	F20-F23, F29
Weibell (2014)	DSM-IV	Schizophrenia, schizoaffective disorder, schizophreniform disorder, delusional disorder, brief psychosis, psychosis NOS	295.3, 295.7, 295.4, 297.1, 298.9, 298.9
Kirkbride (2015)	DSM-IV	Non-affective disorders	295.xx, 297.xx, 298.8, 298.9
Paksarian (2015)	ICD-8 & ICD-10	Non-affective disorders	F20-F29
Paksarian (2015)	ICD-8 & ICD-10	Non-affective disorders	F20-F29
Soderlund (2015)	ICD-10	Non-affective disorders	F20-F29
Anderson (2015)	ICD-10	Non-affective disorders	F20, F25
Szoke (2016)	DSM-IV	Non-affective disorders	295.xx, 297.x, 298.x
Kendler (2016)	ICD-9 & ICD-10	Non-affective disorders	F20-F29
Hollander (2016)	ICD-10	Non-affective disorders	F20-F29
Kirkbride (2016)	ICD-10	Non-affective disorders	F20-F29
Hoeffding (2017)	ICD-8 and ICD-10	Non-affective disorders	F20-F29

Veling (2006)	DSM-IV	Schizophrenia, schizoaffective disorder, schizophreniform disorder	None given
Tarricone (2012)	ICD-10	Non-affective disorders	F20-F29
Singh (2004)	ICD-10	Non-affective disorders	F20-F29
Kirkbride (2006)	DSM-IV	Non-affective disorders	295.xx, 297.xx, 298.8, 298.9
Turner (2006)	ICD-10	Non-affective disorders	F20-F29
Menezes (2006)	DSM-IV	Non-affective disorders	295.10-.90, 297,1, 298.8, 298.8
Kirkbride (2007)	ICD-10	Non-affective disorders	F20-F29
Szoke (2014)	DSM-IV	Non-affective disorders	None given
Lasalvia (2014)	ICD-10	Non-affective disorders	F20-F29
Cocchi (2014)	ICD-10	Non-affective disorders	F20-F29
Jongsma (2018)	ICD-10	Non-affective disorders	F20-F29
Kim (2017)	ICD-10	Non-affective disorders	F20, F25
Kirkbride (2017)	ICD-10	Non-affective disorders	F20-F29
Markkula (2017)	ICD-10	Non-affective disorders	F20-F29
Nyberg (2018)	ICD-8 & ICD-10	Non-affective disorders	F20, F22, F24, F25, F28, F29
Okkels (2017)	ICD-8 & ICD-10	Non-affective disorders	F20-F29
Richardson (2018)	ICD-10	Non-affective disorders	F20-F29
Schofield (2017)	ICD-8 & ICD-10	Non-affective disorders	F20-F29
Schofield (2017)	ICD-8 & ICD-10	Non-affective disorders	F20-F29
Barghadouch (2018)	ICD-10	Non-affective disorders	F20-F29
Vikstrom (2017)	ICD-8 & ICD-10	Non-affective disorders	F20-F29
Norredam (2009)	ICD-10	Non-affective disorders	F20-F29
Norredam (2010)	ICD-10	Non-affective disorders	F20-F29

[1] For all citations including more than one version of the ICD manual, codes referring to the most recent manual are given (in all cases: ICD-10).

Bibliography of Table 1 of the main article.

NB: some studies are also referred to in the body of the main article, and these are included in the reference list at the end of the main article (references up until #60 in Table 1).

61. Tsuchiya KJ, Munk-Jørgensen P. First-admission rates of schizophrenia in Denmark, 1980-1997: Have they been increasing? Schizophr Res. 2002;54(3):187–91.

62. Hanoeman M, Selten J-P, Kahn RS. Incidence of schizophrenia in Surinam. Schizophr Res. 2002;54(3):219–21.

63. Selten J-P, Cantor-Graae E, Slaets J, Kahn RS. Ødegaard's selection hypothesis revisited: schizophrenia in Surinamese immigrants to the Netherlands. Am J Psychiatry. 2002 Apr;159(4):669–71.

64. Baldwin PA, Scully PJ, Quinn JF, Morgan MG, Kinsella A, O'Callaghan E, et al. First episode bipolar disorder: Systematic comparison of incidence with other affective and non-affective psychoses among an epidemiologically complete, rural population. Bipolar Disord. 2002;4(Suppl1):39–40.

65. Scully PJ, Quinn JF, Morgan MG, Kinsella A, Callaghan EO, Owens JM, et al. First-episode schizophrenia, bipolar disorder and other psychoses in a rural Irish catchment area: incidence and gender in the Cavan-Monaghan study at 5 years. Br Journ. 2002;1(181):s3–9.

66. Boydell J, Van Os J, Lambri M, Castle D, Allardyce J, McCreadie RG, et al. Incidence of schizophrenia in south-east London between 1965 and 1997. Br J Psychiatry. 2003;182(JAN.):45–9.

67. Smith G, Lupton L, Honer W. Changes in the incidence of schizophrenia in British Columbia: 1907 to 1913. Schizophr Res. 2003;60(1, Supplement):2003.

68. Singh S, Wright C, Joyce E, Barnes T, Burns TRE. Developing early intervention services in the NHS: A survey to guide workforce and training needs. Psychiatr Bull. 2003;27(7):254–8.

69. Selten JP, van Os J, Nolen WA. First admissions for mood disorders in immigrants to the Netherlands. Soc Psychiatry Psychiatr Epidemiol. 2003;38(10):547–50.

70. Cantor-Graae E, Pedersen CB, McNeil TF, Mortensen PB. Migration as a risk factor for schizophrenia: a Danish population – based cohort study. Br J Psychiatry. 2003;182:117 – 122.

71. Baldwin P, Scully P, Quinn J, Morgan M, Kinsella A, Owens J, et al. Reduced rural incidence of schizophrenia is primarily a female phenomenon: The Cavan-Monaghan first episode study at seven years. Schizophr Res. 2003;60(1, Supplement):33.

72. Proctor SE, Mitford E, Paxton R. First episode psychosis: A novel methodology reveals higher than expected incidence; a reality-based population profile in Northumberland, UK. J Eval Clin Pract. 2004;10(4):539–47.

73. Sipos A, Rasmussen F, Harrison G, Tynelius P, Lewis G, Leon DA, et al. Paternal age and schizophrenia: a population based cohort study. BMJ. 2004;329(7474):1070.

74. Chien I-C, Chou Y-J, Lin C-H, Bih S-H, Chou P. Prevalence of psychiatric disorders among National Health Insurance enrollees in Taiwan. Psychiatry Clin Neurosci. 2004;58(6):611–8.

75. Boydell J, van Os J, McKenzie K, Murray RM. The association of inequality with the incidence of schizophrenia - An ecological study. Soc Psychiatry Psychiatr Epidemiol. 2004;39(8):597–9.

76. Veen ND, Selten JP, Schols D, Laan W, Hoek HW, Van Der Tweel I, et al. Diagnostic stability in a Dutch psychosis incidence cohort. Br J Psychiatry. 2004;185(DEC.):460–4.

77. Singh SP, Burns T, Amin S, Jones PB, Harrison G. Acute and transient psychotic disorders: precursors, epidemiology, course and outcome. Br J Psychiatry. 2004;185:452–9.

78. Sailas ES, Feodoroff B, Virkkunen M, Wahlbeck K. Mental disorders in prison populations aged 15-21: national register study of two cohorts. BMJ. 2005;330(April):1354–64.

79. Harris A, Brennan J, Anderson J, Taylor A, Sanbrook M, Fitzgerald D, et al. Clinical profiles, scope and general findings of the Western Sydney First Episode Psychosis Project. Aust N Z J Psychiatry. 2005;39(1–2):36–43.

80. Sundquist K, Frank G, Sundquist J. Urbanisation and incidence of psychosis and depression: Follow-up study of 4.4 million women and men in Sweden. Br J Psychiatry. 2004;184(APR.):293–8.

81. Nager A, Johansson LM, Sundquist K. Are sociodemographic factors and year of delivery associated with hospital admission for postpartum psychosis? A study of 500 000 first-time mothers. Acta Psychiatr Scand. 2005;112(1):47–53.

82. Laursen TM, Labouriau R, Licht RW, Bertelsen A, Munk-Olsen T, Mortensen PB. Family history of psychiatric illness as a risk factor for schizoaffective disorder: a Danish register-based cohort study. Arch Gen Psychiatry. 2005;62(8):841–8.

83. Selten J-P, Zeyl C, Dwark Asing R, Lumsden V, Kahn RS, van Harten PN. First-contact incidence of schizophrenia in Surinam. Br J Psychiatry. 2005;186:74–5.

84. Nixon NL, Doody GA. Official psychiatric morbidity and the incidence of schizophrenia 1881-1994. Psychol Med. 2005;35(8):1145–53.

85. Qin P, Xu H, Laursen TM, Vestergaard M, Mortensen PB. Risk for schizophrenia and schizophrenia-like psychosis among patients with epilepsy: population based cohort study. BMJ. 2005;331(7507):23.

86. Allardyce J, Gilmour H, Atkinson J, Rapson T, Bishop J, McCreadie RG. Social fragmentation, deprivation and urbanicity : relation to first-admission rates for psychoses. Br J Psychiatry. 2005;187(May):401–6.

87. Cantor-Graae E, Zolkowska K, McNeil TF. Increased risk of psychotic disorder among immigrants in Malmö: a 3-year first-contact study. Psychol Med. 2005;35(8):1155–63.

88. Kennedy N, Boydell J, Kalidindi S, Fearon P, Jones PB, Van Os J, et al. Gender differences in incidence and age at onset of mania and bipolar disorder over a 35-year period in Camberwell, England. Am J Psychiatry. 2005;162(2):257–62.

89. Kennedy N, Everitt B, Boydell J, Van Os J, Jones PB, Murray RM. Incidence and distribution of first-episode mania by age: results from a 35-year study. Psychol Med. 2005;35(6):855–63.

90. Lloyd T, Kennedy N, Fearon P, Kirkbride J, Mallett R, Leff J, et al. Incidence of bipolar affective disorder in three UK cities: Results from the ÆSOP study. Br J Psychiatry. 2005;186(FEB.):126–31.

91. Bray I, Waraich P, Jones W, Slater S, Goldner EM, Somers J. Increase in schizophrenia incidence rates: Findings in a Canadian cohort born 1975-1985. Soc Psychiatry Psychiatr Epidemiol. 2006;41(8):611–8.

92. Payne J, Malla A, Norman R, Windell D, Brown N. Status of first-episode psychosis patients presenting for routine care in a defined catchment area. Can J Psychiatry. 2006;51(1):42–7.

93. Drukker M, Krabbendam L, Driessen G, van Os J. Social disadvantage and schizophrenia: A combined neighbourhood and individual-level analysis. Soc Psychiatry Psychiatr Epidemiol. 2006;41(8):595–604.

94. Turner MA, Finch PJC, McKechanie AG, Kiernan MD, Hawksley OJ, Wadhwani S, et al. Psychosis in the British army: a 2-year follow-up study. Mil Med. 2006 Dec;171(12):1215–9.

95. Mahmmood M, Fisher H. 0548 The incidence of first episode psychosis in inner London: Findings from the Lambeth Early Onset (LEO) service. Schizophr Res. 2006;86:S66–7.

96. Westman J, Johansson LM, Sundquist K. Country of birth and hospital admission rates for mental disorders: a cohort study of 4.5 million men and women in Sweden. Eur Psychiatry. 2006;21(5):307–14.

97. Munk-Olsen T, Laursen TM, Pedersen CB, Mors O, Mortensen PB. New Parents and Mental Disorders. Jama. 2006;296(21):2582–9.

98. Amminger GP, Harris MG, Conus P, Lambert M, Elkins KS, Yuen HP, et al. Treated incidence of first-episode psychosis in the catchment area of EPPIC between 1997 and 2000. Acta Psychiatr Scand. 2006;114(5):337–45.

99. Morgan C, Dazzan P, Morgan K, Jones P, Harrison G, Leff J, et al. First episode psychosis and ethnicity: initial findings from the AESOP study. World Psychiatry. 2006;5(1):40–6.

100. Fearon P, Kirkbride JB, Morgan C, Dazzan P, Morgan K, Lloyd T, et al. Incidence of schizophrenia and other psychoses in ethnic minority groups: results from the MRC AESOP Study. Psychol Med. 2006;36(11):1541–50.

101. Gould M, Theodore K, Pilling S, Bebbington P, Hinton M, Johnson S. Initial treatment phase in early psychosis: Can intensive home treatment prevent admission? Psychiatr Bull. 2006;30(7):243–6.

102. Zipursky RB. Gender, age, ethnicity and area of residence influence incidence of psychotic disorders. Evid Based Ment Health. 2006;9.

103. Li X, Sundquist J, Sundquist K. Age-specific familial risks of psychotic disorders and schizophrenia: A nation-wide epidemiological study from Sweden. Schizophr Res. 2007;97(1–3):43–50.

104. Schimmelmann BG, Conus P, Cotton S, McGorry PD, Lambert M. Pre-treatment, baseline, and outcome differences between early-onset and adult-onset psychosis in an epidemiological cohort of 636 first-episode patients. Schizophr Res. 2007;95(1–3):1–8.

105. Laursen TM, Munk-Olsen T, Nordentoft M, Bo Mortensen P. A comparison of selected risk factors for unipolar depressive disorder, bipolar affective disorder, schizoaffective disorder, and schizophrenia from a danish population-based cohort. J Clin Psychiatry. 2007;68(11):1673–81.

106. Ajdacic-Gross V, Lauber C, Warnke I, Haker H, Murray RM, Rössler W. Changing incidence of psychotic disorders among the young in Zurich. Schizophr Res. 2007;95(1–3):9–18.

107. Andersen JE, Hynnekleiv T. Hospital-treated psychosis and suicide in a rural community (1877-2005). Part 2: Genetic founder effects. Acta Psychiatr Scand. 2007;116(SUPPL. 436):20–32.

108. Harlow BL, Vitonis AF, Sparen P, Cnattingius S, Joffe H, Hultman CM. Incidence of hospitalization for postpartum psychotic and bipolar episodes in women with and without prior prepregnancy or prenatal psychiatric hospitalizations. Arch Gen Psychiatry. 2007;64(1):42–8.

109. Juvonen H, Reunanen A, Haukka J, Muhonen M, Suvisaari J, Arajärvi R, et al. Incidence of schizophrenia in a nationwide cohort of patients with type 1 diabetes mellitus. Arch Gen Psychiatry. 2007;64(8):894–9.

110. Cantor-Graae E, Pedersen CB. Risk for schizophrenia in intercountry adoptees: A Danish population-based cohort study. J Child Psychol Psychiatry Allied Discip. 2007;48(11):1053–60.

111. Cantor-Graae E, Pedersen C. Risk of schizophrenia in second-generation immigrants: a Danish population-based cohort study. Psychol Med. 2007 Apr 4;37(04):485.

112. Leão T, Sundquist J, Johansson LM, Johansson S-E, Sundquist K. Incidence of mental disorders in second-generation immigrants in Sweden: a four-year cohort study. Ethn Health. 2007;10(3):243–56.

113. Kirkbride JB, Fearon P, Morgan C, Dazzan P, Morgan K, Murray RM, et al. Neighbourhood variation in the incidence of psychotic disorders in Southeast London. Soc Psychiatry Psychiatr Epidemiol. 2007;42(6):438–45.

114. Kirkbride JB, Morgan C, Fearon P, Dazzan P, Murray RM, Jones PB. Neighbourhood-level effects on psychoses: re-examining the role of context. Psychol Med. 2007;37(10):1413–25.

115. Stain H, Sartore GM, Andrews D, Kelly B. First-episode psychosis in rural, coastal and remote Australian communities. Australas Psychiatry. 2008;16(2):119–24.

116. Boonstra N, Wunderink L, De Wit PHM, Noorthoorn E, Wiersma D. De administratieve incidentie van niet-affectieve psychosen in Friesland en Twente. Tijdschr Psychiatr. 2008;50(10):637–43.

117. Crebbin K, Mitford E, Paxton R, Turkington D. First-episode psychosis: An epidemiological survey comparing psychotic depression with schizophrenia. J Affect Disord. 2008;105(1–3):117–24.

118. Castagnini A, Bertelsen A, Berrios GE. Incidence and diagnostic stability of ICD-10 acute and transient psychotic disorders. Compr Psychiatry. 2008;49(3):255–61.

119. Weiser M, Werbeloff N, Vishna T, Yoffe R, Lubin G, Shmushkevitch M, et al. Elaboration on immigration and risk for schizophrenia. Psychol Med. 2008 Aug 8;38(08):1113–9.

120. Veling W, Susser E, Van Os J, Mackenbach JP, Selten JP, Hoek HW. Ethnic density of neighborhoods and incidence of psychotic disorders among immigrants. Am J Psychiatry. 2008;165(1):66–73.

121. Kirkbride JB, Boydell J, Ploubidis GB, Morgan C, Dazzan P, McKenzie K, et al. Testing the association between the incidence of schizophrenia and social capital in an urban area. Psychol Med. 2008;38(8):1083–94.

122. Kirkbride JB, Barker D, Cowden F, Stamps R, Yang M, Jones PB, et al. Psychoses, ethnicity and socio-economic status. Br J Psychiatry. 2008;193(1):18–24.

123. Coid JW, Kirkbride JB, Barker D, Cowden F, Stamps R, Yang M, et al. Raised incidence rates of all psychoses among migrant groups: findings from the East London First Episode Psychosis Study. Arch Gen Psychiatry. 2008;65(11):1250–8.

124. Grant BF, Goldstein RB, Chou SP, Huang B, Stinson FS, Dawson DA, et al. Sociodemographic and psychopathologic predictors of first incidence of DSM-IV substance use, mood and anxiety disorders: results from the Wave 2 National Epidemiologic Survey on Alcohol and Related Conditions. Mol Psychiatry. 2009 Nov;14(11):1051–66.

125. Crebbin K, Mitford E, Paxton R, Turkington D. First-episode drug-induced psychosis: A medium term follow up study reveals a high-risk group. Soc Psychiatry Psychiatr Epidemiol. 2009;44(9):710–5.

126. Bih SH, Chien IC, Chou YJ, Lin CH, Lee CH, Chou P. The treated prevalence and incidence of bipolar disorder among national health insurance enrollees in Taiwan, 1996-2003. Soc Psychiatry Psychiatr Epidemiol. 2008;43(11):860–5.

127. Corcoran C, Perrin M, Harlap S, Deutsch L, Fennig S, Manor O, et al. Incidence of Schizophrenia among second-generation immigrants in the Jerusalem perinatal cohort. Schizophr Bull. 2009;35(3):596–602.

128. Ösby U, Tiainen A, Backlund L, Edman G, Adler M, Hällgren J, et al. Psychiatric admissions and hospitalization costs in bipolar disorder in Sweden. J Affect Disord. 2009;115(3):315–22.

129. Valdimarsdottir U, Hultman CM, Harlow B, Cnattingius S, Sparen P. Psychotic illness in first-time mothers with no previous psychiatric hospitalizations: A population-based study. PLoS Med. 2009;6(2):0194–201.

130. Harlap S, Perrin MC, Deutsch L, Kleinhaus K, Fennig S, Nahon D, et al. Schizophrenia and birthplace of paternal and maternal grandfather in the Jerusalem perinatal cohort prospective study. Schizophr Res. 2009;111(1–3):23–31.

131. Reay R, Mitford E, McCabe K, Paxton R, Turkington D. Incidence and diagnostic diversity in first-episode psychosis. Acta Psychiatr Scand. 2010;121(4):315–9.

132. Norredam M, Garcia-Lopez A, Keiding N, Krasnik A. Risk of mental disorders in refugees and native Danes: a register-based retrospective cohort study. Soc Psychiatry Psychiatr Epidemiol. 2009 Dec 18;44(12):1023–9.

133. Bogren M, Mattisson C, Tambs K, Horstmann V, Munk-Jørgensen P, Nettelbladt P. Predictors of psychosis: A 50-year follow-up of the Lundby population. Eur Arch Psychiatry Clin Neurosci. 2010;260(2):113–25.

134. Kirkbride JB, Croudace T, Brewin J, Donoghue K, Mason P, Glazebrook C, et al. Is the incidence of psychotic disorder in decline? Epidemiological evidence from two decades of research. Int J Epidemiol. 2009;38(5):1255–64.

135. Cheng F, Kirkbride JB, Lennox B, Perez J, Masson K, Lawrence K, et al. Incidence of Psychosis in an Early Intervention for Psychosis Service in England: First Epidemiological Evidence From a Diverse, Predominantly Rural Setting. Schizophr Res. 2010;117(2–3):532.

136. Zammit S, Lewis G, Rasbash J, Dalman C, Gustafsson J-E, Allebeck P. Individuals, schools, and neighborhood: a multilevel longitudinal study of variation in incidence of psychotic disorders. ArchGenPsychiatry. 2010;67(9):914–22.

137. Tseng KC, Hemenway D, Kawachi I, Subramanian S V., Chen WJ. The impact of the Chi-Chi earthquake on the incidence of hospitalizations for schizophrenia and on concomitant hospital choice. Community Ment Health J. 2010;46(1):93–101.

138. Zandi T, Havenaar JM, Smits M, Limburg-Okken AG, van Es H, Cahn W, et al. First contact incidence of psychotic disorders among native Dutch and Moroccan immigrants in the Netherlands: Influence of diagnostic bias. Schizophr Res. 2010;119(1–3):27–33.

139. Marie N, Ana G-L, Niels K, Allan K. Risk of mental disorders in family reunification migrants and native Danes: a register-based historically prospective cohort study. Int J Public Health. 2010 Oct 30;55(5):413–9.

140. Cowan DN, Weber NS, Fisher JA, Bedno SA, Niebuhr DW. Incidence of adult onset schizophrenic disorders in the US Military: Patterns by sex, race and age. Schizophr Res. 2011;127(1–3):235–40.

141. Harris M, Farquhar F, Healy D, Le Noury J, Baker D, Whitaker C, et al. The incidence and prevalence of admissions for melancholia in two cohorts (1875-1924 and 1995-2005). J Affect Disord. 2011;134(1–3):45–51.

142. Jörgensen L, Ahlbom A, Allebeck P, Dalman C. The Stockholm non-affective psychoses study (snaps): The importance of including out-patient data in incidence studies. Acta Psychiatr Scand. 2010;121(5):389–92.

143. Cheng F, Kirkbride JB, Lennox BR, Perez J, Masson K, Lawrence K, et al. Administrative incidence of psychosis assessed in an early intervention service in England: first epidemiological evidence from a diverse, rural and urban setting. Psychol Med. 2011;41:949–58.

144. Kleinhaus K, Harlap S, Perrin M, Manor O, Weiser M, Lichtenberg P, et al. Age, sex and first treatment of schizophrenia in a population cohort. J Psychiatr Res. 2011;45(1):136–41.

145. Benros ME, Nielsen PR, Nordentoft M, Eaton WW, Dalton SO, Mortensen PB. Autoimmune diseases and severe infections as risk factors for schizophrenia: A 30-year population-based register study. Am J Psychiatry. 2011;168(12):1303–10.

146. Salokangas RKR, Helminen M, Koivisto AM, Rantanen H, Oja H, Pirkola S, et al. Incidence of hospitalised schizophrenia in Finland since 1980: Decreasing and increasing again. Soc Psychiatry Psychiatr Epidemiol. 2011;46(4):343–50.

147. Schofield P, Ashworth M, Jones R. Ethnic isolation and psychosis: re-examining the ethnic density effect. Psychol Med. 2011;41(6):1263–9.

148. Veling W, Hoek HW, Selten J-P, Susser E. Age at migration and future risk of psychotic disorders among immigrants in the Netherlands: a 7 -year incidence study. Am J Psychiatry. 2011;168(12):1278–85.

149. Healy D, Le Noury J, Linden SC, Harris M, Whitaker C, Linden D, et al. The incidence of admissions for schizophrenia and related psychoses in two cohorts: 1875-1924 and 1994-2010. BMJ Open. 2012;2(1):1.

150. Callaghan RC, Cunningham JK, Allebeck P, Arenovich T, Sajeev G, Remington G, et al. Methamphetamine Use and Schizophrenia: A Population-Based Cohort Study in California. Am J Psychiatry. 2012 Apr;169(4):389–96.

151. Anderson KK, Fuhrer R, Abrahamowicz M, Malla AK. The incidence of first-episode schizophrenia-spectrum psychosis in adolescents and young adults in Montreal: An estimate from an administrative claims database. Can J Psychiatry. 2012;57(10):626–33.

152. Manrique-Garcia E, Zammit S, Dalman C, Hemmingsson T, Andreasson S, Allebeck P. Cannabis, schizophrenia and other non-affective psychoses: 35 years of follow-up of a population-based cohort. Psychol Med. 2012;42(06):1321–8.

153. Turola MC, Comellini G, Galuppi A, Nanni MG, Carantoni E, Scapoli C. Schizophrenia in real life: courses, symptoms and functioning in an Italian population. Int J Ment Health Syst. 2012;6(1):22.

154. Werbeloff N, Levine SZ, Rabinowitz J. Elaboration on the association between immigration and schizophrenia: A population-based national study disaggregating annual trends, country of origin and sex over 15 years. Soc Psychiatry Psychiatr Epidemiol. 2012;47(2):303–11.

155. Nosarti C, Reichenberg A, Murray RM, Cnattingius S, Lambe MP, Yin L, et al. Preterm birth and psychiatric disorders in young adult life. ArchGenPsychiatry. 2012;69(1538–3636 (Electronic)):E1--E8.

156. Gigantesco A, Lega I, Picardi A. The Italian SEME surveillance system of severe mental disorders presenting to community mental health services. Clin Pract Epidemiol Ment Health. 2012;8:7–11.

157. Kirkbride JB, Stubbins C, Jones PB. Psychosis incidence through the prism of early intervention services. Br J Psychiatry. 2012;200(2):156–7.

158. Hung Y-P, Liu C-J, Tsai C-F, Hung M-H, Tzeng C-H, Liu C-Y, et al. Incidence and risk of mood disorders in patients with breast cancers in Taiwan: a nationwide population-based study. Psychooncology. 2013 Mar 1;22(10):n/a-n/a.

159. Peritogiannis V, Mantas C, Tatsioni A, Mavreas V. Rates of first episode of psychosis in a defined catchment area in Greece. Clin Pract Epidemiol Ment Health. 2013;9:251–4.

160. Sutterland AL, Dieleman J, Storosum JG, Voordouw BAC, Kroon J, Veldhuis J, et al. Annual incidence rate of schizophrenia and schizophrenia spectrum disorders in a longitudinal population-based cohort study. Soc Psychiatry Psychiatr Epidemiol. 2013;48(9):1357–65.

161. Cantor-Graae E, Pedersen CB. Full Spectrum of Psychiatric Disorders Related to Foreign Migration. JAMA Psychiatry. 2013;70(4):427.

162. Kroon JS, Wohlfarth TD, Dieleman J, Sutterland AL, Storosum JG, Denys D, et al. Incidence rates and risk factors of bipolar disorder in the general population: A population-based cohort study. Bipolar Disord. 2013;15(3):306–13.

163. Castagnini A, Foldager L. Variations in incidence and age of onset of acute and transient psychotic disorders. Soc Psychiatry Psychiatr Epidemiol. 2013;48(12):1917–22.

164. Hardoon S, Hayes JF, Blackburn R, Petersen I, Walters K, Nazareth I, et al. Recording of severe mental illness in United Kingdom primary care, 2000-2010. PLoS One. 2013;8(12).

165. Weibell MA, Joa I, Bramness J, Johannessen JO, McGorry PD, Ten Velden Hegelstad W, et al. Treated incidence and baseline characteristics of substance induced psychosis in a Norwegian catchment area. BMC Psychiatry. 2013;13:319.

166. Cocchi A, Balbi A, Corlito G, Ditta G, Di Munzio W, Nicotera M, et al. Early intervention in psychosis: A feasibility study financed by the Italian Center on Control of Maladies. Early Interv Psychiatry. 2015;9(2):163–71.

167. Tortelli A, Morgan C, Szöke A, Nascimento A, Skurnik N, Monduit De Caussade E, et al. Different rates of first admissions for psychosis in migrant groups in Paris. Soc Psychiatry Psychiatr Epidemiol. 2014;49(7):1109–1109.

168. Pedersen L, Simonsen E. Incidence and prevalence rates of personality disorders in Denmark-A register study. Nord J Psychiatry. 2014;68(8):543–8.

169. Sørensen HJ, Nielsen PR, Pedersen CB, Benros ME, Nordentoft M, Mortensen PB. Population impact of familial and environmental risk factors for schizophrenia: A nationwide study. Schizophr Res. 2014;153(1–3):214–9.

170. Munk-Olsen T, Bech BH, Vestergaard M, Li J, Olsen J, Laursen TM. Psychiatric disorders following fetal death: a population-based cohort study. BMJ Open. 2014;1–6.

171. Szöke A, Charpeaud T, Galliot A-M, Vilain J, Richard J-R, Leboyer M, et al. Rural-urban variation in incidence of psychosis in France: a prospective epidemiologic study in two contrasted catchment areas. BMC Psychiatry. 2014;14:78.

172. Bhavsar V, Boydell J, Murray R, Power P. Identifying aspects of neighbourhood deprivation associated with increased incidence of schizophrenia. Schizophr Res. 2014;156(1):115–21.

173. Omer S, Kirkbride JB, Pringle DG, Russell V, O'Callaghan E, Waddington JL. Neighbourhood-level socio-environmental factors and incidence of first episode psychosis by place at onset in rural Ireland: The Cavan-Monaghan First Episode Psychosis Study [CAMFEPS]. Schizophr Res. 2014;152(1):152–7.

174. Veling W, Susser E, Selten J-P, Hoek HW. Social disorganization of neighborhoods and incidence of psychotic disorders: a 7-year first-contact incidence study. Psychol Med. 2014;45(09):1789–98.

175. Paksarian D, Eaton WW, Mortensen PB, Merikangas KR, Pedersen CB. A population-based study of the risk of schizophrenia and bipolar disorder associated with parent-child separation during development. Psychol Med. 2015;1–13.

176. Sørensen HJ, Larsen JT, Mors O, Nordentoft M, Mortensen PB, Petersen L. Analysis of risk factors for schizophrenia with two different case definitions: A nationwide register-based external validation study. Schizophr Res. 2015;162(1–3):74–8.

177. Paksarian D, Eaton WW, Mortensen PB, Pedersen CB. Childhood Residential Mobility, Schizophrenia, and Bipolar Disorder: A Population-based Study in Denmark. Schizophr Bull. 2015;41(2):346–54.

178. Söderlund J, Wicks S, Jörgensen L, Dalman C. Comparing cohort incidence of schizophrenia with that of bipolar disorder and affective psychosis in individuals born in Stockholm County 1955-1967. Psychol Med. 2015;1990:1–7.

179. Medici CR, Videbech P, Gustafsson LN ørgreen, Munk-Jørgensen P. Mortality and secular trend in the incidence of bipolar disorder. J Affect Disord. 2015;183(April):39–44.

180. Carlborg A, Ferntoft L, Thuresson M, Bodegard J. Population study of disease burden, management, and treatment of bipolar disorder in Sweden: A retrospective observational registry study. Bipolar Disord. 2015;17(1):76–85.

181. Tsai P-J, Liao Y-T, Lee CT-C, Hsu C-Y, Hsieh M-H, Tsai C-J, et al. Risk of bipolar disorder in patients with COPD: a population-based cohort study. Gen Hosp Psychiatry. 2016 Jul 1;41:6–12.

182. Chen PM, Chen SC, Liu CJ, Hung MH, Tsai CF, Hu YW, et al. The association between prostate cancer and mood disorders: a nationwide population-based study in Taiwan. Int Psychogeriatr. 2015;27(3):481–90.

183. Latvala A, Kuja-Halkola R, Rück C, D'Onofrio BM, Jernberg T, Almqvist C, et al. Association of Resting Heart Rate and Blood Pressure in Late Adolescence With Subsequent Mental Disorders. JAMA Psychiatry. 2016 Dec 1;73(12):1268.

184. Mohr Jensen C, Steinhausen H-C. Time Trends in Lifetime Incidence Rates of First-Time Diagnosed Bipolar and Depressive Disorders Across 16 Years in Danish Psychiatric Hospitals. J Clin Psychiatry. 2016 Dec 28;77(12):e1570–5.

185. Kühl JOG, Laursen TM, Thorup A, Nordentoft M. The incidence of schizophrenia and schizophrenia spectrum disorders in Denmark in the period 2000–2012. A register-based study. Schizophr Res. 2016 Oct;176(2–3):533–9.

186. Filatova S, Marttila R, Koivumaa-Honkanen H, Nordström T, Veijola J, Mäki P, et al. A comparison of the cumulative incidence and early risk factors for psychotic disorder in young adults in the Northern Finland Birth Cohorts 1966 and 1986. Epidemiol Psychiatr Sci. 2017 Jun 28;26(03):314–24.

187. Nielsen PR, Laursen TM, Agerbo E. Comorbidity of schizophrenia and infection: a population-based cohort study. Soc Psychiatry Psychiatr Epidemiol. 2016 Dec 19;51(12):1581–9.

188. Levine SZ, Levav I, Goldberg Y, Pugachova I, Becher Y, Yoffe R. Exposure to genocide and the risk of schizophrenia: a population-based study. Psychol Med. 2015;1–9.

189. Levine SZ, Levav I, Pugachova I, Yoffe R, Becher Y. Transgenerational effects of genocide exposure on the risk and course of schizophrenia: A population-based study. Schizophr Res. 2016 Oct 1;176(2–3):540–5.

190. Sørensen HJ, Gamborg M, Sørensen TIA, Baker JL, Mortensen EL. Childhood body mass index and risk of schizophrenia in relation to childhood age, sex and age of first contact with schizophrenia. Eur Psychiatry. 2016 Apr 1;34:64–9.

191. Hollander A-C, Dal H, Lewis G, Magnusson C, Kirkbride JB, Dalman C. Refugee migration and risk of schizophrenia and other non-affective psychoses: cohort study of 1.3 million people in Sweden. BMJ. 2016;352.

192. O'Donoghue B, Lyne JP, Renwick L, Lane A, Madigan K, Staines A, et al. Neighbourhood characteristics and the incidence of first-episode psychosis and duration of untreated psychosis. Psychol Med. 2016 May 5;46(07):1367–78.

193. Tarricone I, Boydell J, Kokona A, Triolo F, Gamberini L, Sutti E, et al. Risk of psychosis and internal migration: Results from the Bologna First Episode Psychosis study. Schizophr Res. 2016;(173):90–3.

194. Szöke A, Pignon B, Baudin G, Tortelli A, Richard J-R, Leboyer M, et al. Small area-level variation in the incidence of psychotic disorders in an urban area in France: an ecological study. Soc Psychiatry Psychiatr Epidemiol. 2016;51:951–60.

195. Okkels N, Trabjerg B, Arendt M, Pedersen CB. Traumatic Stress Disorders and Risk of Subsequent Schizophrenia Spectrum Disorder or Bipolar Disorder: A Nationwide Cohort Study. Schizophr Bull. 2017 Jan 1;43(1):180–6.

196. Vikström J, Josefsson A, Hammar M, Bladh M, Sydsjö G. Risk of postpartum psychosis after IVF treatment: a nationwide case-control study. Hum Reprod. 2016 Dec 6;32(1):139–46.

197. Wang W-C, Lu M-L, Chen VC-H, Ng M-H, Huang K-Y, Hsieh M-H, et al. Asthma, corticosteroid use and schizophrenia: A nationwide population-based study in Taiwan. PLoS One. 2017 Mar;12(3).

198. Lin C-Y, Chang F-W, Yang J-J, Chang C-H, Yeh C-L, Lei W-T, et al. Increased risk of bipolar disorder in patients with scabies: A nationwide population-based matched-cohort study. Psychiatry Res. 2017 Nov 1;257:14–20.

199. Marrie RA, Walld R, Bolton JM, Sareen J, Walker JR, Patten SB, et al. Increased incidence of psychiatric disorders in immune-mediated inflammatory disease. J Psychosom Res. 2017 Oct 1;101:17–23.

200. Marrie RA, Walld R, Bolton JM, Sareen J, Walker JR, Patten SB, et al. Rising incidence of psychiatric disorders before diagnosis of immune-mediated inflammatory disease. Epidemiol Psychiatr Sci. 2017 Nov 3;1–10.

201. Hogerzeil SJ, van Hemert AM, Veling W, Hoek HW. Incidence of schizophrenia among migrants in the Netherlands: a direct comparison of first contact longitudinal register approaches. Soc Psychiatry Psychiatr Epidemiol. 2017;52(2):147–54.

202. Hoeffding LK, Trabjerg BB, Olsen L, Mazin W, Sparsø T, Vangkilde A, et al. Risk of Psychiatric Disorders Among Individuals With the 22q11.2 Deletion or Duplication. JAMA Psychiatry. 2017 Mar 1;74(3):282.

203. Nielsen SM, Toftdahl NG, Nordentoft M, Hjorthøj C. Association between alcohol, cannabis, and other illicit substance abuse and risk of developing schizophrenia: a nationwide population based register study. Psychol Med. 2017 Jul 7;47(09):1668–77.

204. Schofield P, Thygesen M, Das-Munshi J, Becares L, Cantor-Graae E, Pedersen C, et al. Ethnic density, urbanicity and psychosis risk for migrant groups - A population cohort study. Schizophr Res. 2017 Dec 1;190:82–7.

205. Kirkbride JB, Hameed Y, Ankireddypalli G, Ioannidis K, Crane CM, Nasir M, et al. The epidemiology of first-episode psychosis in early intervention in psychosis services: findings From the Social Epidemiology of Psychoses in East Anglia [SEPEA] study. Am J Psychiatry. 2017;174(2):143–53.

206. Kirkbride JB, Hameed Y, Ioannidis K, Ankireddypalli G, Crane CM, Nasir M, et al. Ethnic Minority Status, Age-at-Immigration and Psychosis Risk in Rural Environments: Evidence From the SEPEA Study. Schizophr Bull. 2017 Oct 21;43(6):1251–61.

207. Schofield P, Thygesen M, Das-Munshi J, Becares L, Cantor-Graae E, Agerbo E, et al. Neighbourhood ethnic density and psychosis — Is there a difference according to generation? Schizophr Res. 2018 May 1;195:501–5.

208. Nyberg J, Henriksson M, Åberg MAI, Rosengren A, Söderberg M, Åberg ND, et al. Cardiovascular fitness in late adolescent males and later risk of serious non-affective mental disorders: a prospective, population-based study. Psychol Med. 2018 Feb 28;48(03):416–25.

209. Barghadouch A, Carlsson J, Norredam M. Psychiatric Disorders and Predictors Hereof Among Refugee Children in Early Adulthood A Register-Based Cohort Study. J Nerv Ment Dis. 2018;206(1).

210. Richardson L, Hameed Y, Perez J, Jones PB, Kirkbride JB. Association of Environment With the Risk of Developing Psychotic Disorders in Rural Populations: Findings from the Social Epidemiology of Psychoses in East Anglia Study. JAMA Psychiatry. 2018;75(1):75–83.

Bibliography of Supplemental Material

1. Harris A, Brennan J, Anderson J, et al. Clinical profiles, scope and general findings of the Western Sydney First Episode Psychosis Project. *Aust N Z J Psychiatry*. 2005;39(1-2):36-43. doi:10.1111/j.1440-1614.2005.01517.x

2. Amminger GP, Harris MG, Conus P, et al. Treated incidence of first-episode psychosis in the catchment area of EPPIC between 1997 and 2000. *Acta Psychiatr Scand*. 2006;114(5):337-345. doi:10.1111/j.1600-0447.2006.00790.x

3. Cheng F, Kirkbride JB, Lennox B, et al. Incidence of Psychosis in an Early Intervention for Psychosis Service in England: First Epidemiological Evidence From a Diverse, Predominantly Rural Setting. *Schizophr Res*. 2010;117(2-3):532. doi:10.1016/j.schres.2010.02.1046

4. Cheng F, Kirkbride JB, Lennox BR, et al. Administrative incidence of psychosis assessed in an early intervention service in England: first epidemiological evidence from a diverse, rural and urban setting. *Psychol Med*. 2011;41:949-958. doi:10.1017/S0033291710002461

5. Kirkbride JB, Hameed Y, Ankireddypalli G, et al. The epidemiology of first-episode psychosis in early intervention in psychosis services: findings From the Social Epidemiology of Psychoses in East Anglia [SEPEA] study. *Am J Psychiatry*. 2017;174(2):143-153. doi:10.1176/appi.ajp.2016.16010103

6. Kirkbride JB, Hameed Y, Ioannidis K, et al. Ethnic Minority Status, Age-at-Immigration and Psychosis Risk in Rural Environments: Evidence From the SEPEA Study. *Schizophr Bull*. 2017;43(6):1251-1261. doi:10.1093/schbul/sbx010

7. Richardson L, Hameed Y, Perez J, Jones PB, Kirkbride JB. Association of Environment With the Risk of Developing Psychotic Disorders in Rural Populations: Findings from the Social Epidemiology of Psychoses in East Anglia Study. *JAMA Psychiatry*. 2018;75(1):75-83. doi:10.1001/jamapsychiatry.2017.3582

8. Filatova S, Marttila R, Koivumaa-Honkanen H, et al. A comparison of the cumulative incidence and early risk factors for psychotic disorder in young adults in the Northern Finland Birth Cohorts 1966 and 1986. *Epidemiol Psychiatr Sci*. 2017;26(03):314-324. doi:10.1017/S2045796016000123

9. Kirkbride JB, Stubbins C, Jones PB. Psychosis incidence through the prism of early intervention services. *Br J Psychiatry*. 2012;200(2):156-157. doi:10.1192/bjp.bp.111.094896

10. Anderson KK, Cheng J, Susser E, McKenzie KJ, Kurdyak P. Incidence of psychotic disorders among first-generation immigrants and refugees in Ontario. *Can Med Assoc J*. 2015;187(9):E279-E286. doi:10.1503/cmaj.150494

11. Söderlund J, Wicks S, Jörgensen L, Dalman C. Comparing cohort incidence of schizophrenia with that of bipolar disorder and affective psychosis in individuals born in Stockholm County 1955-1967. *Psychol Med*. 2015;1990:1-7. doi:10.1017/S0033291715001336

12. Hollander A-C, Dal H, Lewis G, Magnusson C, Kirkbride JB, Dalman C. Refugee migration and risk of schizophrenia and other non-affective psychoses: cohort study of 1.3 million people in Sweden. *BMJ*. 2016;352. doi:10.1136/bmj.i1030

13. Barghadouch A, Carlsson J, Norredam M. Psychiatric Disorders and Predictors Hereof Among Refugee Children in Early Adulthood A Register-Based Cohort Study. *J Nerv Ment Dis*. 2018;206(1). doi:10.1097/NMD.0000000000000576

14. Corcoran C, Perrin M, Harlap S, et al. Incidence of Schizophrenia among second-generation immigrants in the Jerusalem perinatal cohort. *Schizophr Bull*. 2009;35(3):596-602. doi:10.1093/schbul/sbn089

15. Leão T, Sundquist J, Johansson LM, Johansson S-E, Sundquist K. Incidence of mental disorders in second-generation immigrants in Sweden: a four-year cohort study. *Ethn Health*. 2007;10(3):243-256. doi:10.1080/13557850500096878

16. Leão TS, Sundquist J, Frank G, Johansson L-M, Johansson S-E, Sundquist K. Incidence of schizophrenia or other psychoses in first- and second-generation immigrants: a national cohort study. *J Nerv Ment Dis*. 2006;194(1):27-33. doi:10.1097/01.nmd.0000195312.81334.81

17. Ajdacic-Gross V, Lauber C, Warnke I, Haker H, Murray RM, Rössler W. Changing incidence of psychotic disorders among the young in Zurich. *Schizophr Res*. 2007;95(1-3):9-18. doi:10.1016/j.schres.2007.06.001

18. Kleinhaus K, Harlap S, Perrin M, et al. Age, sex and first treatment of schizophrenia in a population cohort. *J Psychiatr Res*. 2011;45(1):136-141. doi:10.1016/j.jpsychires.2010.05.010

19. Nosarti C, Reichenberg A, Murray RM, et al. Preterm birth and psychiatric disorders in young adult life. *ArchGenPsychiatry*. 2012;69(1538-3636 (Electronic)):E1--E8. doi:10.1001/archgenpsychiatry.2011.1374

20. Anderson KK, Fuhrer R, Abrahamowicz M, Malla AK. The incidence of first-episode schizophrenia-spectrum psychosis in adolescents and young adults in Montreal: An estimate from an administrative claims database. *Can J Psychiatry*. 2012;57(10):626-633. doi:10.1177/070674371205701007

21. Cantor-Graae E, Pedersen CB. Full Spectrum of Psychiatric Disorders Related to Foreign Migration. *JAMA Psychiatry*. 2013;70(4):427. doi:10.1001/jamapsychiatry.2013.441

22. Paksarian D, Eaton WW, Mortensen PB, Pedersen CB. Childhood Residential Mobility, Schizophrenia, and Bipolar Disorder: A Population-based Study in Denmark. *Schizophr Bull*. 2015;41(2):346-354. doi:10.1093/schbul/sbu074

23. Bray I, Waraich P, Jones W, Slater S, Goldner EM, Somers J. Increase in schizophrenia incidence rates: Findings in a Canadian cohort born 1975-1985. *Soc Psychiatry Psychiatr Epidemiol*. 2006;41(8):611-618. doi:10.1007/s00127-006-0073-z

24. Selten J-P, Cantor-Graae E, Slaets J, Kahn RS. Ødegaard's selection hypothesis revisited: schizophrenia in Surinamese immigrants to the Netherlands. *Am J Psychiatry*. 2002;159(4):669-671. doi:10.1176/appi.ajp.159.4.669

25. R. J, N. G, A. M, et al. Is higher primary care quality associated with lower hospital admissions for people with serious mental illness? *J Ment Health Policy Econ*. 2013;16:S15-S16. http://ovidsp.ovid.com/ovidweb.cgi?T=JS&PAGE=reference&D=emed12&NEWS=N&AN=71383729.

26. Stain H, Sartore GM, Andrews D, Kelly B. First-episode psychosis in rural, coastal and remote Australian communities. *Australas Psychiatry*. 2008;16(2):119-124. doi:10.1080/10398560701802177

27. Qin P, Xu H, Laursen TM, Vestergaard M, Mortensen PB. Risk for schizophrenia and schizophrenia-like psychosis among patients with epilepsy: population based cohort study. *BMJ*. 2005;331(7507):23. doi:10.1136/bmj.38488.462037.8F

28. Juvonen H, Reunanen A, Haukka J, et al. Incidence of schizophrenia in a nationwide cohort of patients with type 1 diabetes mellitus. *Arch Gen Psychiatry*. 2007;64(8):894-899. doi:10.1001/archpsyc.64.8.894

29. Okkels N, Trabjerg B, Arendt M, Pedersen CB. Traumatic Stress Disorders and Risk of Subsequent Schizophrenia Spectrum Disorder or Bipolar Disorder: A Nationwide Cohort Study. *Schizophr Bull*. 2017;43(1):180-186. doi:10.1093/schbul/sbw082

30. Wang W-C, Lu M-L, Chen VC-H, et al. Asthma, corticosteroid use and schizophrenia: A nationwide population-based study in Taiwan. *PLoS One*. 2017;12(3). doi:10.1371/journal.pone.0173063

31. Benros ME, Nielsen PR, Nordentoft M, Eaton WW, Dalton SO, Mortensen PB. Autoimmune diseases and severe infections as risk factors for schizophrenia: A 30-year population-based register study. *Am J Psychiatry*. 2011;168(12):1303-1310. doi:10.1176/appi.ajp.2011.11030516

32. Hung Y-P, Liu C-J, Tsai C-F, et al. Incidence and risk of mood disorders in patients with breast cancers in Taiwan: a nationwide population-based study. *Psychooncology*. 2013;22(10):n/a-n/a. doi:10.1002/pon.3277

33. Chen PM, Chen SC, Liu CJ, et al. The association between prostate cancer and mood disorders: a nationwide population-based study in Taiwan. *Int Psychogeriatr*. 2015;27(3):481-490. doi:10.1017/s104161021400218x

34. Tsai P-J, Liao Y-T, Lee CT-C, et al. Risk of bipolar disorder in patients with COPD: a population-based cohort study. *Gen Hosp Psychiatry*. 2016;41:6-12. doi:10.1016/J.GENHOSPPSYCH.2016.04.004

35. Lin C-Y, Chang F-W, Yang J-J, et al. Increased risk of bipolar disorder in patients with scabies: A nationwide population-based matched-cohort study. *Psychiatry Res*. 2017;257:14-20. doi:10.1016/J.PSYCHRES.2017.07.013

36. Marrie RA, Walld R, Bolton JM, et al. Increased incidence of psychiatric disorders in immune-mediated inflammatory disease. *J Psychosom Res*. 2017;101:17-23. doi:10.1016/j.jpsychores.2017.07.015

37. Marrie RA, Walld R, Bolton JM, et al. Rising incidence of psychiatric disorders before diagnosis of immune-mediated inflammatory disease. *Epidemiol Psychiatr Sci*. November 2017:1-10. doi:10.1017/S2045796017000579

38. Nielsen SM, Toftdahl NG, Nordentoft M, Hjorthøj C. Association between alcohol, cannabis, and other illicit substance abuse and risk of developing schizophrenia: a nationwide population based register study. *Psychol Med*. 2017;47(09):1668-1677. doi:10.1017/S0033291717000162

39. Goodman GP, DeZee KJ, Burks R, Waterman BR, Belmont PJ. Epidemiology of psychiatric disorders sustained by a U.S. Army brigade combat team during the Iraq War. *Gen Hosp Psychiatry*. 2011;33(1):51-57. doi:10.1016/j.genhosppsych.2010.10.007

40. Cowan DN, Weber NS, Fisher JA, Bedno SA, Niebuhr DW. Incidence of adult onset schizophrenic disorders in the US Military: Patterns by sex, race and age. *Schizophr Res*. 2011;127(1-3):235-240. doi:10.1016/j.schres.2010.12.005

41. Manrique-Garcia E, Zammit S, Dalman C, Hemmingsson T, Andreasson S, Allebeck P. Cannabis, schizophrenia and other non-affective psychoses: 35 years of follow-up of a population-based cohort. *Psychol Med*. 2012;42(06):1321-1328. doi:10.1017/S0033291711002078

42. Latvala A, Kuja-Halkola R, Rück C, et al. Association of Resting Heart Rate and Blood Pressure in Late Adolescence With Subsequent Mental Disorders. *JAMA Psychiatry*. 2016;73(12):1268. doi:10.1001/jamapsychiatry.2016.2717

43. Turner MA, Finch PJC, McKechanie AG, et al. Psychosis in the British army: a 2-year follow-up study. *Mil Med*. 2006;171(12):1215-1219.

44. Nyberg J, Henriksson M, Åberg MAI, et al. Cardiovascular fitness in late adolescent males and later risk of serious non-affective mental disorders: a prospective, population-based study. *Psychol Med*. 2018;48(03):416-425. doi:10.1017/S0033291717001763

45. Ramsey C, Dziura J, Justice AC, et al. Incidence of Mental Health Diagnoses in Veterans of Operations Iraqi Freedom, Enduring Freedom, and New Dawn. *Am J Public Heal*. 2017;107(10):329-335. doi:10.2105/AJPH.2016.303574

46. Weiser M, Werbeloff N, Vishna T, et al. Elaboration on immigration and risk for schizophrenia. *Psychol Med*. 2008;38(08):1113-1119. doi:10.1017/S003329170700205X

47. Valdimarsdottir U, Hultman CM, Harlow B, Cnattingius S, Sparen P. Psychotic illness in first-time mothers with no previous psychiatric hospitalizations: A population-based study. *PLoS Med*. 2009;6(2):0194-0201. doi:10.1371/journal.pmed.1000013

48. Nager A, Johansson LM, Sundquist K. Are sociodemographic factors and year of delivery associated with hospital admission for postpartum psychosis? A study of 500 000 first-time mothers. *Acta Psychiatr Scand*. 2005;112(1):47-53. doi:10.1111/j.1600-0447.2005.00525.x

49. Munk-Olsen T, Laursen TM, Pedersen CB, Mors O, Mortensen PB. New Parents and Mental Disorders. *Jama*. 2006;296(21):2582-2589. doi:10.1001/jama.296.21.2582

50. Munk-Olsen T, Bech BH, Vestergaard M, Li J, Olsen J, Laursen TM. Psychiatric disorders following fetal death: a population-based cohort study. *BMJ Open*. 2014:1-6. doi:10.1136/bmjopen-2014-005187

51. Vikström J, Josefsson A, Hammar M, Bladh M, Sydsjö G. Risk of postpartum psychosis after IVF treatment: a nationwide case-control study. *Hum Reprod*. 2016;32(1):139-146. doi:10.1093/humrep/dew302

52. Sailas ES, Feodoroff B, Virkkunen M, Wahlbeck K. Mental disorders in prison populations aged 15-21: national register study of two cohorts. *BMJ*. 2005;330(April):1354-1364.

doi:10.1136/bmj.38415.633762.F7

53. World Bank. World Bank Country and Lending Groups. https://datahelpdesk.worldbank.org/knowledgebase/articles/906519-world-bank-country-and-lending-groups. Published 2018. Accessed March 5, 2019.

54. Tortelli A, Morgan C, Szöke A, et al. Different rates of first admissions for psychosis in migrant groups in Paris. *Soc Psychiatry Psychiatr Epidemiol*. 2014;49(7):1109-1109. doi:10.1007/s00127-013-0795-7

55. Veling W, Selten JP, Veen N, Laan W, Blom JD, Hoek HW. Incidence of schizophrenia among ethnic minorities in the Netherlands: A four-year first-contact study. *Schizophr Res*. 2006;86(1-3):189-193. doi:10.1016/j.schres.2006.06.010

56. Hogerzeil SJ, van Hemert AM, Veling W, Hoek HW. Incidence of schizophrenia among migrants in the Netherlands: a direct comparison of first contact longitudinal register approaches. *Soc Psychiatry Psychiatr Epidemiol*. 2017;52(2):147-154. doi:10.1007/s00127-016-1310-8

57. Cantor-Graae E, Pedersen C. Risk of schizophrenia in second-generation immigrants: a Danish population-based cohort study. *Psychol Med*. 2007;37(04):485. doi:10.1017/S0033291706009652
